# Identification of microRNAs in the Toxigenic Dinoflagellate *Alexandrium catenella* by High-Throughput Illumina Sequencing and Bioinformatic Analysis

**DOI:** 10.1371/journal.pone.0138709

**Published:** 2015-09-23

**Authors:** Huili Geng, Zhenghong Sui, Shu Zhang, Qingwei Du, Yuanyuan Ren, Yuan Liu, Fanna Kong, Jie Zhong, Qingxia Ma

**Affiliations:** Key Laboratory of Marine Genetics and Breeding of Ministry of Education, Ocean University of China, Qingdao, 266003, China; Huazhong University of Science and Technology, CHINA

## Abstract

Micro-ribonucleic acids (miRNAs) are a large group of endogenous, tiny, non-coding RNAs consisting of 19–25 nucleotides that regulate gene expression at either the transcriptional or post-transcriptional level by mediating gene silencing in eukaryotes. They are considered to be important regulators that affect growth, development, and response to various stresses in plants. *Alexandrium catenella* is an important marine toxic phytoplankton species that can cause harmful algal blooms (HABs). To date, identification and function analysis of miRNAs in *A*. *catenella* remain largely unexamined. In this study, high-throughput sequencing was performed on *A*. *catenella* to identify and quantitatively profile the repertoire of small RNAs from two different growth phases. A total of 38,092,056 and 32,969,156 raw reads were obtained from the two small RNA libraries, respectively. In total, 88 mature miRNAs belonging to 32 miRNA families were identified. Significant differences were found in the member number, expression level of various families, and expression abundance of each member within a family. A total of 15 potentially novel miRNAs were identified. Comparative profiling showed that 12 known miRNAs exhibited differential expression between the lag phase and the logarithmic phase. Real-time quantitative RT-PCR (qPCR) was performed to confirm the expression of two differentially expressed miRNAs that were one up-regulated novel miRNA (aca-miR-3p-456915), and one down-regulated conserved miRNA (tae-miR159a). The expression trend of the qPCR assay was generally consistent with the deep sequencing result. Target predictions of the 12 differentially expressed miRNAs resulted in 1813target genes. Gene ontology (GO) analysis and the Kyoto Encyclopedia of Genes and Genomes pathway database (KEGG) annotations revealed that some miRNAs were associated with growth and developmental processes of the alga. These results provide insights into the roles that miRNAs play in the growth of *A*. *catenella*, and they provide the basis for further studies of the molecular mechanisms that underlie bloom growth in red tides species.

## Introduction

As a class of biflagellated unicellular microorganisms, the free-swimming dinoflagellates play an important role in the primary production of the marine ecosystem[1]. Inthe past two decades, it has been suggested that marine dinoflagellates, which were previously deemed to be exclusively autotrophic,are actually mixotrophic (i.e., they carry out photosynthesis and ingest prey) [2–4]. Importantly, many dinoflagellate species can produce toxins[5], and they account for 75% of all harmful algal bloom species(HABs).Blooms of toxic dinoflagellates have detrimental effects on the fishing industry, marine environments, and public health[6–8].

Within the dinoflagellate genus *Alexandrium*, many species can produce neurotoxins called paralytic shellfish toxins (PSTs), which can bring about paralytic shellfish poisoning. In the last few decades, the frequency and intensity of HABs caused by this genus have increased, as has their geographic extent [9]. *Alexandrium catenella* is widely distributed all over the world and has the ability to produce PSTs [10]. Previous studies of *A*. *catenella* focused on transcriptome sequencing and analysis[11], proteomic analysis[12], characterization of genes related to proliferating cells[13], and gene expression in the decline phase of HABs[14] and the effect of different environmental conditions[15], which aimed to elucidate the molecular mechanisms responsible for blooming. However, molecular mechanisms related to the regulation of cell growth during the process of HAB development remain unclear[12, 13]. Moreover, no investigation of micro-ribonucleic acids (miRNAs) and their expression profiles in *A*. *catenella* have been reported to date.

miRNAs are a large class of evolutionarily conserved, endogenous, non-coding, single-stranded, tiny (18–25 nucleotide (nt)) RNAs that are ubiquitous in eukaryotes[16]. They play many crucial roles in various biological and cellular processes, such as cell proliferation, differentiation[17], signal transduction[18], and responses to stresses[19] in plants. Mature miRNAs are loaded into the RNA-induced silencing complex, where they can generally guide direct transcript cleavage to regulate gene expression[20]. As a class of master regulators of gene expression, considerable efforts have been made to discover and profile miRNAs from various plant species. In recent decades, Sanger sequencing and computational prediction methods were initially used to identify miRNAs[21, 22].However, some species-specific or specific-expression miRNAs with low abundance are not conservative[23, 24], and traditional methods may not be able to detect these miRNAs.

Next-generation sequencing is not only a rapid and powerful approach to investigating genomes, but it also is a tool that can provide unprecedented coverage depth, which is advantageous for discovering low abundance or novel miRNAs[25]. For many higher organisms, such as *Arachishypogaea* [26], *Oryza sativa*[27],and *Medicagotruncatula* [28], high-throughput sequencing was successfully used after first being used in the model organism *Arabidopsis*[29]. Recently, miRNAs were found to be expressed in macroalgae (*Ectocarpussiliculosus* [30], *Ulva prolifera* [31], and *Pyropiayezoensis* [32]) and a limited number of unicellular algae (*Chlamydomonasreinhardtii*[33], *Phaeodactylumtricornutum*[34], and *Symbiodiniummicroadriaticum*[35]).At present, a comprehensive profile of the endogenous miRNAs and their regulatory roles in *Alexandrium* obtained by high-throughput sequencing does not exist,although18 potential miRNAs were screened from expressed sequence tags in *Alexandriumtamarense* using a computational approach[36].

In this study, two small RNA libraries from different growth stages of *A*. *catenella* were constructed and sequenced using an Illumina high-throughput platform. The sequence data were analyzed using an automated, proprietary, bioinformatics analysis pipeline. The aim of this study was to profile differential expression of miRNAs between the two growth phases and to determine their probable roles in the growth of *A*. *catenella*. qPCR was conducted to validate the differential expression of identified miRNAs. To our knowledge, this is the first comprehensive report of identification and profiling of miRNAs from *Alexandrium* by high-throughput Illumina sequencing. Results of this study will provide new insights into the roles of miRNAs in the growth of *Alexandrium* species and lay the foundation for further study of the molecular regulatory mechanisms involved in HAB development.

## Materials and Methods

### Sample culture and collection

*A*. *catenella* was obtained from the Key Laboratory of Marine Genetics and Breeding, Ministry of Education of China, College of Marine Life Sciences. The stock algal cells were maintained at 20±1°C in f/2 medium(without silicate) with a 12 h/12 h light/dark photocycle[37]. Cool white fluorescent lights with a photon flux density of 30–35 μmol m^–2^ s^–l^ provided the light. To construct separate small RNA libraries, the cultures were grown into logarithmic phase in f/2 medium (without silicate)and placed in the dark for 48 h[38].Subsequently, the synchronized cells were inoculated into new f/2 medium with an initial concentration of 2×10^6^ cellsL^–l^. Algal cells at two different growth stages were sampled at 12:00. One sample was the lag phase sample (cells cultured for 2 dinf/2 medium), and the other was the logarithmic phase sample(a mixture of cells cultured for 7 d and 12 d, respectively).Both samples contained 5×10^6^ cells. Because many reports have noted that high concentrations of nitrogen (N), phosphorus (P), and manganese (Mn) promote the growth of *A*. *catenella*[39, 40],three nutrient-enhanced cultures(i.e., induced logarithmic phases)with high N, high P, and high Mn were generated and used for the quantitative PCR (qPCR) experiment. The f/2 medium in these cultures was supplied with NaNO_3_,NaH_2_PO_4_⋅2H_2_OorMnCl_2_⋅4H_2_Oat final concentrations of 2.304mmolL^–l^, 0.144mmolL^–l^, and 2.730μmol/L^–l^[11], respectively. Cells (5×10^6^) were collected from each of the induced logarithmic phases of the three nutrient-enhanced cultures. To minimize bacterial contamination, methods described in previous studies were used [41, 42]. Briefly, a 10μm pore size bolting-silk was used to filter every 100mLof the cultures, which was rinsed with 300mL of sterile f/2 medium.

The *A*. *catenella* cultures were subjected to the following treatments: Washed cells were suspended in 50 mL of sterile f/2 medium containing 0.05% Tween-80 and 0.01 M EDTA (at 20°C for 30 min), followed by ultrasonication for 1 min (50 w; ultrasonic treatment time of 5 s with an interval of 5 s). Sequentially, lysozyme (0.5 mgmL^–l^, 20°C for 10 min) and sodium dodecyl sulfonate(SDS) (0.25%, 20°C for 10 min) were added. The algal cells then were washed three times with sterile f/2 medium to remove these reagents. Finally, the cultures were detected using epifluorescence microscopy (Nikon ECLIPSE 80i, Japan) after acridine orange staining (0.01%) for 1 or 2 min[42].Only axenic cultures were used for the following experiments. The 5×10^6^cells of different growth stages were collected by centrifugation for 6 min at 4000g.Thecell pellets in 1.5mL Eppendorf tubes were frozen immediately in liquid nitrogen and stored at –80°C prior to RNA extraction. All samples were collected in triplicate.

### Small RNA library construction for Illumina sequencing

Trizolreagent (Invitrogen, Carlsbad, CA,USA) was used to extract total RNA from the lag phase and logarithmic phase samples according to the manufacturer’s instructions, and subsequently DNA was digested with DNase I (TaKaRa, Japan).The quantity and purity of total RNA was analyzed using the NanoDrop 2000 device (Thermo, USA). Only high-quality RNA was selected for subsequent experiments. Approximately 1 μg of total RNA was used to prepare the small RNA(sRNA) library according to the protocol of the TruSeq ^TM^ Small RNA Sample Preparation Kit (Illumina).Briefly, fragments of 15–40ntsRNAs were separated by 15% denaturing polyacrylamide gel electrophoresis (PAGE) using aTrackIt^TM^ 10 base pair (bp) DNA Ladder (Invitrogen).Synthesis of cDNA using SuperScriptIIReverse Transcriptase (Life Technologies) was carried out after ligating 3’ and 5’RNA adapters with T4 RNA ligase. Subsequently, the sRNAs were subjected to PCR amplification. Final amplification products of approximately 147bp were further purified by 6% PAGE. Finally, an Illumina Hiseq2000 instrument was used to sequence the constructed libraries directly. Sequencing reads could be obtained from the image files generated by the Illumina instrument analyzer.

### Bioinformatic processing of Illumina sequencing data

The raw reads were cleaned by masking the low-quality data, adaptor sequence, polyAsequence, and sequences outside of 18–25nt fraction range. The clean reads were processed for miRNA identification with the proprietary in-house software package ACGT101-miR v4.2 (LC Sciences, Houston, TX, USA).Briefly, the filtered reads of 18–25nt were aligned with the Rfam11.0 (ftp.sanger.ac.uk/pub/databases/Rfam)andRepbase 18.02(http://www.girinst.org/) database entries to discard a variety of non-coding sRNA (such as rRNA, tRNA, snRNA, and snoRNA)and repeat sequences (repetitive sequence elements). Subsequently, BLAST searches were used to detect known miRNAs against miRBase 21.0 (http://www.mirbase.org/) with only one mismatch permitted. The priority of the mapped species is listed in S1 Table. To identify potential novel miRNAs, the remaining sequences were further aligned with the transcriptome data to determine their locations. For every sequence mapped to the transcriptome, the sequence of the candidate miRNAs precursor were predicted by RNA fold (http://rna.tbi.univie.ac.at/cgi-bin/RNAfold.cgi) from the adjacent upstream and downstream 60ntsequences in both directions. The following were the default parameters of the RNA fold software: (1) the number of nucleotides in one bulge in the stem was ≤12;(2) the number of bp in the stem region of the predicted hairpin was ≥16;(3) the free energy was ≤ –15 kCalmol^–1^; (4) the length of the hairpin (up and down stems + terminal loop) was ≥ 50; (5) the length of the hairpin loop was ≤200; (6) the number of nucleotides in one bulge in the mature region was ≤ 4; (7) the number of allowed biased errors in one bulge in the mature region was ≤ 4; (8) the number of allowed biased bulges in the mature region was ≤ 2; (9)the number of allowed errors was ≤ 2; (10)the number of bp in the mature region of the predicted hairpin was ≥12; and (11)the percentage of miRNA in the stem was≥ 80%.A sequence was considered to be a candidate miRNA precursor if it satisfied the criteria according to Meyers[43]. Finally, Mfold software(http://mfold.rna.albany.edu/) was used to fold the secondary structures of miRNA[44].To profile the differential expression of identified miRNAs, the raw copies of miRNAs in the two small RNA libraries were normalized[45]. The Fisher exact test and chi-square test with a Bonferroni correction were used for multiple comparisons between the two libraries. The significance threshold was set at 0.05. The fold-change between the two different growth phases was calculated as:

$$\text{fold-change}={\text{log}}_{2}\left(\text{lag phase}/\text{logarithmic phase}\right).$$

If an miRNA satisfied |log_2_fold-change|>1 and *P*< 0.05, it was considered to be a differentially expressed miRNA. It was considered to be significant expression when *P*<0.01 and |log_2_fold-change|>1[46].

### Target prediction for differentially expressed miRNAs and function annotation

To further understand the function of the differentially expressed miRNAs, computational target prediction algorithms (TargetScan5.0 and miRanda 3.3a) were used to predict the miRNA binding sites (potential target genes) [47]. All target gene candidates were mapped to gene ontology (GO) terms in Gene Ontology (http://www.geneontology.org/), and the gene numbers in each term were calculated. To identify significantly enriched GO terms, a hypergeometric test was utilized to compare the target gene candidates with the reference gene background following the formula for *P*-value[48]:

$$P=1\text{-}\sum_{i=0}^{m\text{-}1}\frac{\left(\begin{array}{c} M\\ i \end{array}\right)\left(\begin{array}{c} M\;\text{-}\;N\\ n\;\text{-}\;i \end{array}\right)}{\left(\begin{array}{c} N\\ n \end{array}\right)}$$

N represents the number of all genes with GO annotation, n is the number of differentially expressed target gene candidates in N, M is the number of all genes annotated to a certain GO term, and m is the number of potential target genes in M. Fisher’s exact test also was used to determine the *P*-value. GO terms with a *P*-valueless than the threshold value0.05 were considered to be significantly enriched. The Kyoto Encyclopedia of Genes and Genomes (KEGG, http://www.genome.jp/kegg) was used to analyze metabolic pathway assignments. The formula and threshold value for estimating significantly enriched metabolic pathways and signal transduction pathways were the same as those used in the GO analysis. However, the meanings of the letters in the formula for KEGG analysis were as follows: N represents the number of all genes with KEGG annotation; n is the number of differentially expressed target gene candidates in N, M is the number of all genes annotated to a certain pathway, and m is the number of target gene candidates in M.

### Quantitative real-time PCR of miRNA

To validate the existence of *A*. *catenella* miRNAs identified by the high-throughput sequencing and their expression trends, two miRNAs were selected. Poly(A)-tailed qPCR [49] was conducted using an Applied Biosystems 7500 Real-Time PCR System (Applied Biosystems Ltd, USA).In addition to the lag phase and logarithmic phase samples inf/2 medium, the three induced logarithmic phases (high N, high P, and high Mn cultures)were also analyzed byqPCR. Algal cells of different growth stages and conditions were collected. Each sample contained 5×10^6^cells. The small RNAs (<200 nt) were extracted from the samples following the manufacturer's instructions for the RNAiso for Small RNA kit(TaKaRa).The integrity and quality of sRNAs was evaluated by 3% agarose gel electrophoresis and the NanoDrop 2000 (Thermo, USA).Equal amounts of high-quality sRNAs (OD 260/280 ranging from 1.9 to 2.1) were reverse transcribed to cDNA. Each reverse transcriptase assay consisted of 10 μL of 2× miRNA Reaction Buffer Mix, 2μL of 0.1%BSA, 2μLof miRNA PrimeScript® RT Enzyme Mix, approximately 1μg of total RNA, and 5μL of RNase-free ddH2O.The resulting cDNA products were stored at −20°C. The cDNA was subsequently used for qPCR with miRNA-specific forward primers, which were designed based on mature miRNA sequences, and universal reverse primer, which was provided in the kit. Appropriate adjustments were made by adding several guanine (G)or cytosine(C)residues to the 5’ terminus of the miRNA sequence to obtain mature miRNATm values of about 60°C.All primers used are listed in S2 Table. The qPCR reaction mixture consisted of SYBR® Premix Ex Taq™II(10 μl), 10mM specific forward primer(0.4 μl), 10mMuniversal reverse primer(0.4 μl), RNase-free H2O(7.2 μl), and cDNA (approximately 2 μl) according to the manufacturer's instructions of the SYBR® PrimeScriptx™miRNA RT-PCR Kit (TaKaRa, Japan). The 5.8s RNA gene of *A*. *catenella* was chosen to normalize the expression levels of miRNAs. Reactions for three technical replicates and three biological replicates were prepared. The program for the miRNA qPCR was 95°C for 30s and 40 cycles of 95°C for 5 s, 60°C for 34 s. The comparative threshold (2^–ΔΔCt^) method was used to assess the relative expression levels. Data for mean Ct value and means ±SE (standard error) were calculated for each miRNA. A paired t-test was performed to identify statistically significant differences. *P*<0.05 was deemed to be statistically significant.

## Results

### Construction of small RNA libraries and high-throughput sequencing data analysis

**Fig 1:**
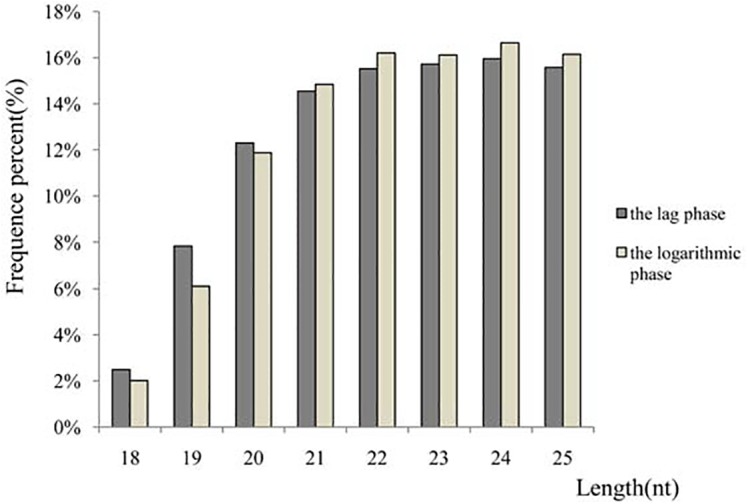
Length distribution and abundance of sequenced small RNA sequences in *A*. *catenella* libraries. Among the small RNAs in the range of 18–25 nt, the most abundant size was 24 nt, followed by 22 nt and 25 nt.

To identify miRNAs involved in the growth of the *A*. *catenella*, Illumina deep sequencing technology was used on two small RNA libraries that were constructed from two different developmental life stages, namely the lag phase(cultured for 2d) and the logarithmic phase (mix of cultures grown for 7 d and 12 d, respectively). A total of 38,092,056(representing 9,311,258 unique reads) and 32,969,156(representing 7,713,621 unique reads) raw reads (SRP055721) were obtained from the lag phase and the logarithmic phase libraries, respectively. Subsequently, the sequence data were further refined to16,527,710 (43.39% of the raw reads) and 13,324,372(40.41% of the raw reads)clean reads of 18–25ntfor the lag phase and logarithmic phase libraries (Table 1). Among the unique reads,3,230,369(34.69% of the unique reads in the lag phase library) and 2,411,293 (31.26% of the unique reads in the logarithmic phase)were found to be similar to miRNAs. The unique read ratios of the miRNA candidates in the respective libraries were 34.69% and 31.26%.Several other types of non-coding RNAs, including tRNA, rRNA, snRNAs, and snoRNAs, were identified by alignment with the Rfam database. The number and proportion of different categories of small RNAs are given in Table 2.When the length distribution of 18–25nt silencing small RNAs in *A*. *catenella* was investigated (Fig 1),the most abundant reads fell into the 24nt fraction, which accounted for 15.96% and16.65%of two libraries, respectively, followed by the 22nt (15.52%, 16.21%), 25 nt(15.58%, 16.15%), and 23nt(15.72% and 16.12%)fractions.

**Table 1:** Data statistics of small RNAs produced by Illumina deep sequencing.

Category	The lag phase		The logarithmic phase	
	Total small RNAs	Unique small RNAs	Total small RNAs	Unique small RNAs
**Raw reads**	38,092,056	9,311,258	32,969,156	7,713,621
**3ADT&length filter**	19,081,207	5,670,799	17,749,062	4,952,497
**Junk reads**	205,372	61,494	213,241	52,070
**Rfam**	2,203,115	284,387	1,623,385	246,854
**Repeats**	106,331	93,061	84,599	74,144
**Clean reads**	16,527,710	3,230,369	13,324,372	2,411,293

**Table 2:** Distribution of small RNAs among different RNA categories in *A*. *catenella* by mapping to Rfam database.

Category	The lag phase		The logarithmic phase	
	Total small RNAs	Unique small RNAs	Total small RNAs	Unique small RNAs
**rRNA**	1,617,108(4.25℅)	135,768(0.36℅)	1,197,675(3.63℅)	123,465(0.37℅)
**tRNA**	70,911(0.19℅)	23,706(0.06℅)	58,754(0.18℅)	24,125(0.07℅)
**snoRNA**	42,572(0.11℅)	30,220(0.08℅)	31,274(0.09℅)	23,939(0.07℅)
**snRNA**	59,056(0.16℅)	23,287(0.06℅)	46,852(0.14℅)	18,316(0.06℅)
**other Rfam RNA** ^**1**^	413,468(1.09℅)	71,406(0.19℅)	288,830(0.88℅)	57,009(0.17℅)

^1^snoRNA:small nucleolar RNA; snRNA: small nuclear RNA; other Rfam RNA: riboswitch, ribozyme, splicing and so on.

### Identification and analysis of known miRNAs in *A*. *catenella*

**Fig 2:**
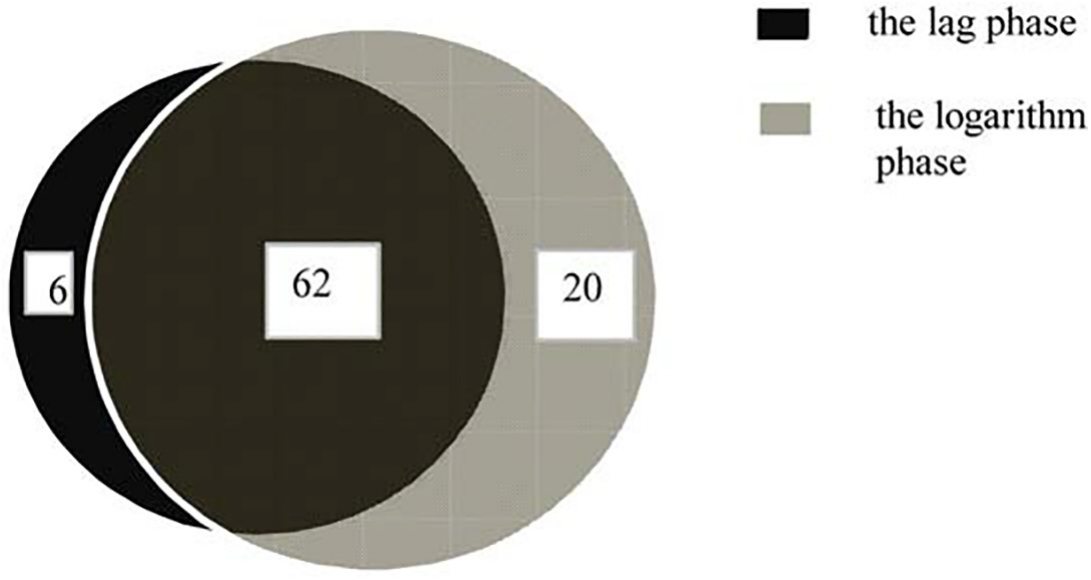
Venn diagram of identified known miRNAs in the two libraries. The diagram not only shows the numbers of miRNA that were expressed in the lag phase and the logarithmic phase preferentially but also the co-expressed miRNAs in both phases.

To identify the expression of known miRNAs in *A*. *catenella*,3,230,369 unique sRNA sequences in the lag phase and 2,411,293 unique sRNA sequences in the logarithmic phase were aligned against known miRNAs and miRNA precursors of selected species(S1 Table) in miRBase (ftp://mirbase.org/pub/mirbase/CURRENT/21.0), with a maximum of one mismatch allowed. Based on the sequence similarity,88 mature miRNAs belonging to 32 miRNA families were identified in the two libraries. Among them, 62 known co-expressed miRNAs were present, and 83 miRNA precursors were also detected. he Venn diagram in Fig 2 displays the distribution of the 88known mature miRNAs between the two libraries.

**Fig 3:**
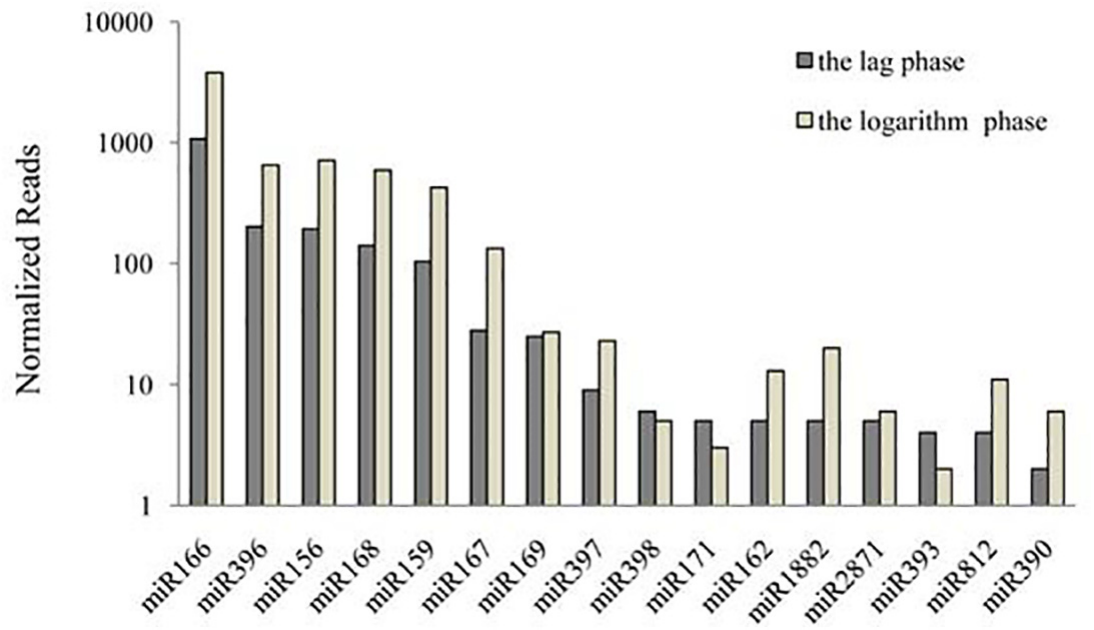
Expression abundance of miRNA families in two growth phases in *A*. *catenella*. X-axis represents the known miRNA families, Y-axis represents the normalized expression level of each miRNA family.

The sequences obtained by high-throughput sequencing made it possible to compare not only the relative abundance of various miRNA families but also that of different members within an miRNA family. Fig 3 illustrates the expression level of miRNA families with more than two copies. Among them, miR166 exhibited the highest abundance in both libraries, with 1076 and 3738 copies respectively. The expression of miR396 ranked second in the lag phase with 202copies, and the expression level of miR156 ranked second with 713 copies in the logarithmic phase. Some families (miR171, miR393, and miR390) had an expression level lower than 10 copies in both phases, whereas each of the following five miRNAs were expressed abundantly(>100 copies) in both phases: miR166, miR396, miR156, miR168, and miR159. These dominantly expressed miRNAs may play important roles in cell growth duringthe two different stages. Overall, these results indicate that the expression level of different families varied greatly.

Within a given miRNA family, the relative abundance of different members varied widely. For example, miR166a exhibited extremely high expression (1851 copies), whereas miR166m had only 3copies in the log phase library. Similarly, in the miR396 family, miR396a expressed 650 copiesbutmiR396e expressed only 3copies. This result implies that largely divergent functions may exist within one miRNA family, which was previously reported in chicken [50].

**Fig 4:**
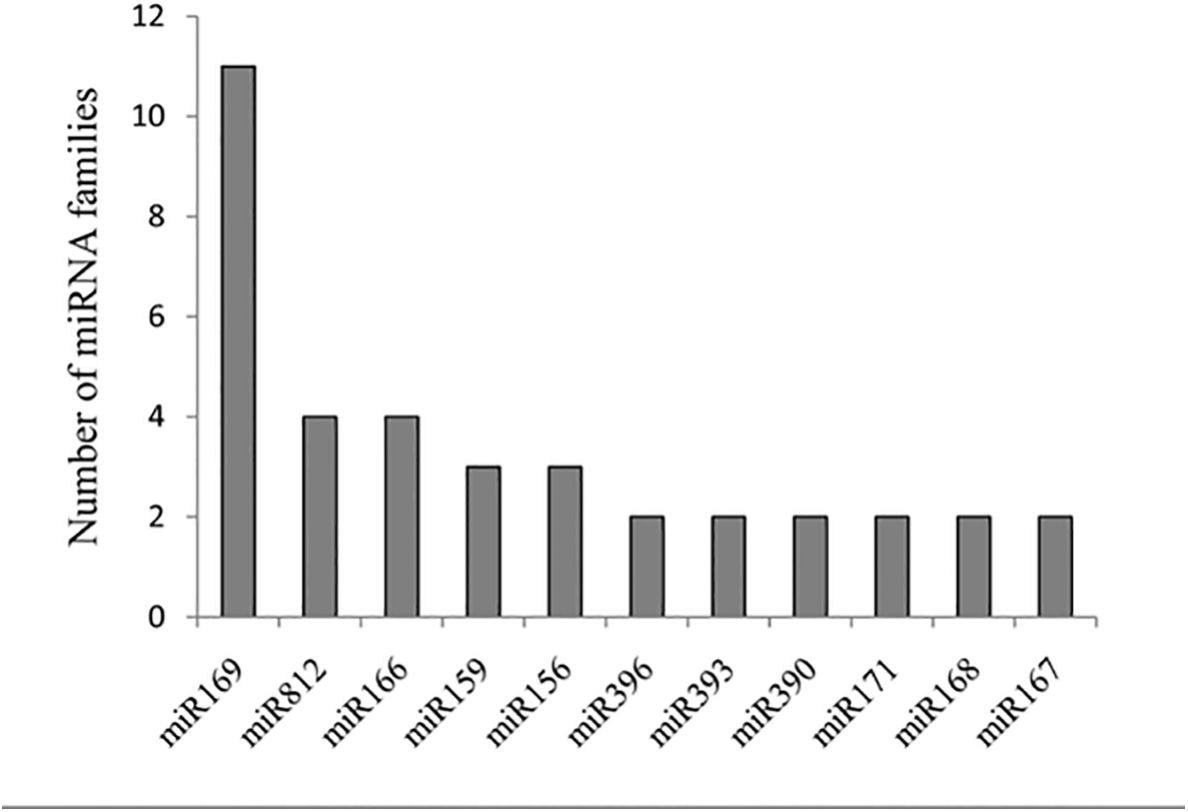
miRNA families with more than two members.

The number of members present in different miRNA families may be indicative of the diversity of *A*. *catenella* miRNA families. Fig 4 shows all of the miRNA families that contained more than two members. Among them, miR169 had the most members (11), miR182 and miR166 ranked second, whereas many families only had one member. Similar results were found in *Arabidopsis thaliana* and *Caragana intermedia*; these studies suggested that the size of miRNA families might be indicative of their function[51, 52].

**Fig 5:**
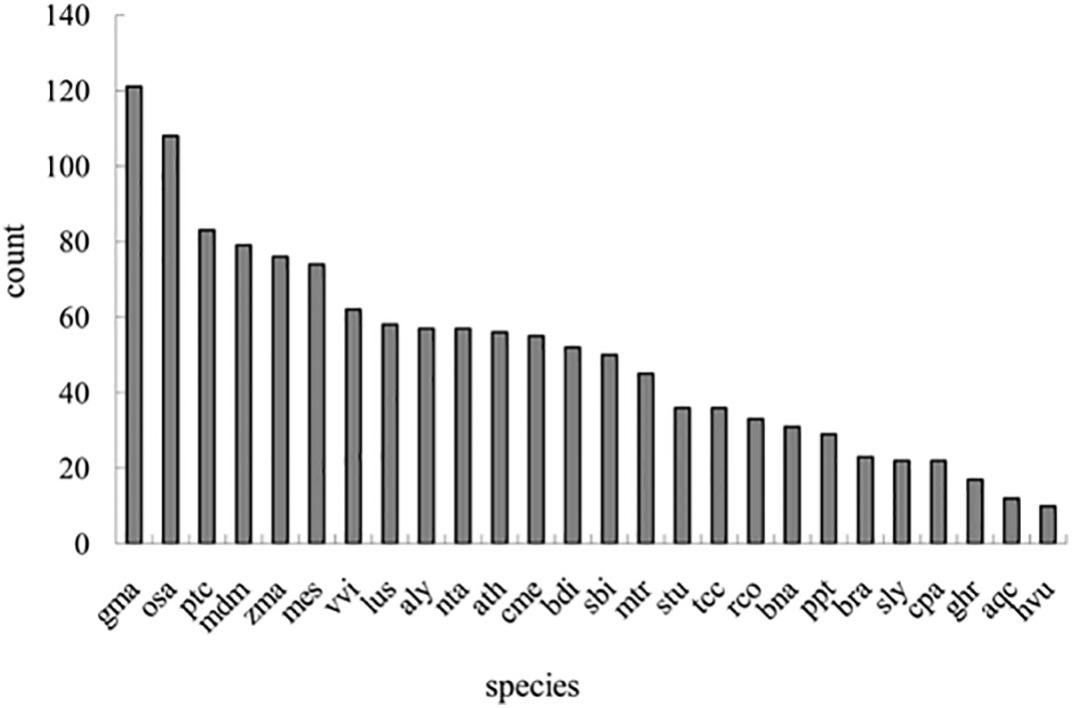
The occurrence of *A*. *catenella* miRNAs that appeared also in other selected species. Gma: *Glycine max*, osa: *Oryza sativa*, ptc: *Populus trichocarpa*, mdm: *Malus domestica*, zma: *Zea mays*, mes: *Manihot esculenta*, vvi: *Vitis vinifera*, lus: *Linum usitatissimum*, aly: *Arabidopsis lyrata*, nta: *Nicotiana tabacum*, ath: *Arabidopsis thaliana*, cme: *Cucumis melo*, bdi: *Brachypodium distachyon*, sbi: *Sorghum bicolor*, mtr: *Medicago truncatula*, stu: *Solanum tuberosum*, tcc: *Theobroma cacao*, rco: *Ricinus communis*, bna: *Brassica napus*, ppt: *Physcomitrella patens*, bra: *Brachypodium distachyon*, sly: *Solanum lycopersicum*, cpa: *Carica papaya*, ghr: *Gossypium hirsutum*, aqc: *Aquilegia caerulea*, hvu: *Hordeum vulgare*, Gma: *Glycine max*, osa: *Oryza sativa*, ptc: *Populus trichocarpa*, mdm: *Malus domestica*, zma: *Zea mays*, mes: *Manihot esculenta*, vvi: *Vitis vinifera*, lus: *Linum usitatissimum*, aly: *Arabidopsis lyrata*, nta: *Nicotiana tabacum*, ath: *Arabidopsis thaliana*, cme: *Cucumis melo*, bdi: *Brachypodium distachyon*, sbi: *Sorghum bicolor*, mtr: *Medicago truncatula*, stu: *Solanum tuberosum*, tcc: *Theobroma cacao*, rco: *Ricinus communis*, bna: *Brassica napus*, ppt: *Physcomitrella patens*, bra: *Brachypodium distachyon*, sly: *Solanum lycopersicum*, cpa: *Carica papaya*, ghr: *Gossypium hirsutum*, aqc: *Aquilegia caerulea*, hvu: *Hordeum vulgare*.

Studies have shown that miRNAs are highly conserved among various organisms. The identified miRNAs in the *A*. *catenella* libraries were further compared with those of other selected species, and the frequency of occurrence in those species was determined (Fig 5). As expected, miRNAs in *A*. *catenella* were identified in many higher plants, including *Glycine max*, *O*. *sativa*, *Populustrichocarpa*, and *Malusdomestica*. Only miR1158 and miR1160 were shared by *C*. *reinhardtii*, which might be attributable to the limited miRNA information for algal organisms in miRBase. These results indicate that miRNAs are highly conserved across species.

**Fig 6:**
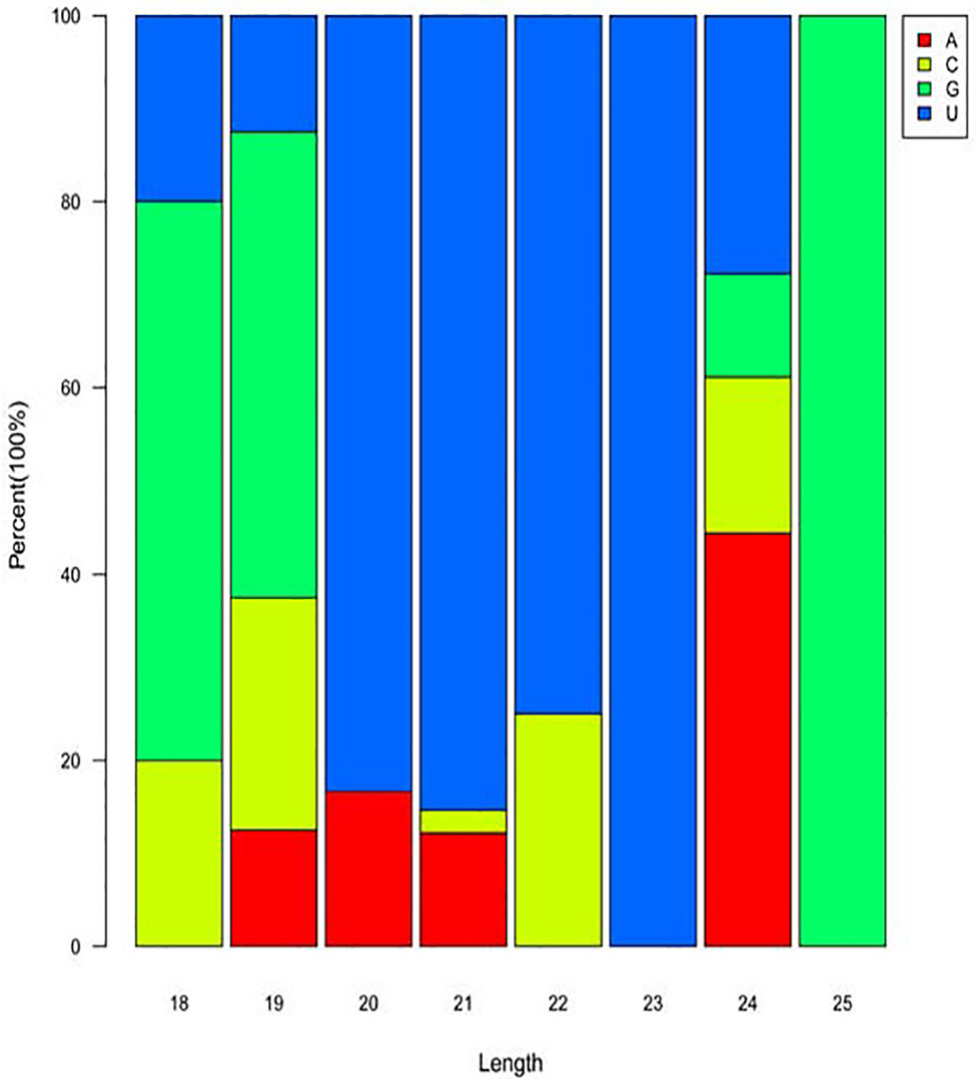
First nucleotide bias of known miRNAs in *A*. *catenella*. The figure indicates the first nucleotide of 18–25 nt miRNAs. U had the greatest frequency among miRNAs of 20–23 nt.

It has been reported that the first residue at the 5’terminus of the vast majority of 20–23 nt small RNAs is predominantly uridine (U) in the plant kingdom[53]. Studies of *Arabidopsis* have indicated that the incorporation of sRNAs into Argonaute proteins(located in silencing complexes) mainly relies on a 5' terminal U[35]. Accordingly, the 5’nucleotides of the 88 identified known miRNAs were analyzed (Fig 6). The first residue of the 20 nt small RNA was either U and adenine (A). The 22 nt small RNAs had a bias for U and C. Small RNAs of 21 nt started with U, C, or A. The base at the first position of all 23 nt small RNAs was U exclusively. Thus, U was dominant in the first position of 20–23 nt small RNAs, which is consistent with previous studies of other organisms, such as *S*.*microadriaticum*, *Physcomitrella patensprotonema*, *Arabidopsis*, and *Triticumaestivum* L [35, 54–56].These results provide support for the existence of miRNA functionality in *A*. *catenella*[55].

### Prediction of novel *A*. *catenella*-specific miRNAs

Following the identification of conserved miRNAs, sequences without similarity to known miRNAs in miRBase were further examined to uncover additional specific miRNAs in *A*. *catenella*. After mapping to the transcriptome information, 15 potentially new miRNAs in the two libraries fulfilled the criteria established for identification of miRNA of higher eukaryotes in compliance with Meyers et al.[43]. These novel candidates mainly ranged from 20 to 24nt(Table 3). A primary criterion for distinguishing miRNA from siRNA and piRNA is that the flanking sequences of miRNA are capable of folding back into a hairpin structure. The secondary hairpin structures of the precursors were predicted by RNA fold (http://rna.tbi.univie.ac.at/cgi-bin/RNAfold.cgi). The precursors of all 15 candidate miRNAs could fold into the typical hairpin structure(S1 Fig–S15 Fig). The average length of precursor sequences(approximately 123nt) was shorter than the average length of known miRNAs (163nt). The minimum free folding energies of these predicted novel miRNA precursors ranged from −46kcalmol^–1^ to −132 kcalmol^–1^(average,−78.32kcalmol^–1^)according to the RNAfold analysis. These results are in line with the values of other miRNA precursors reported previously(−68.2kcalmol^–1^ in *S*.*microadriaticum*, −72.4kcalmol^–1^ in *T*.*aestivum*, and −70kcalmol^–1^ in *O*.*sativa*[35, 56, 57]),but they are lower than the free folding energies of other non-coding RNAs such as tRNAs or rRNAs[58].

**Table 3:** Novel miRNAs predicted in *A*. *catenella*.

Name	Sequence	length	Precursor length	GC%	dG
**aca-miR43924-5p**	GCGGAGGAAAAGAAACTAAC	20	96	52.50	-93.20
**aca- miR4542336-3p**	GTTCCGCTTTCTCGTCGC	18	96	52.50	-90.30
**aca- miR35336-5p**	CGGACTTGCCATTCCTAGCCT	21	187	42.10	-98.10
**aca- miR311414-3p**	AAAGGCTAGGAATTGCAATTC	21	187	42.10	-98.10
**aca- miR129519-3p-1**	CCATCAGCTGTTGCCATGTCGT	22	123	53.50	-73.90
**aca- miR129519-3p-2**	CCATCAGCTGTTGCCATGTCGT	22	175	52.80	-132.30
**aca- miR312912-5p**	AAAAGGCTGGGAATTGCAATTC	22	122	55.70	-73.20
**aca- miR5257085-3p**	GAAGGGCTGGAGATTGCAACT	21	122	55.70	-73.20
**aca- miR456915-3p**	CAAAATGGGCGGCAAGAAAGGCT	23	103	55.70	-67.80
**aca- miR342562-5p**	TTTTTTAAAAGGCTGGGAAT	20	56	38.70	-33.30
**aca- miR126213-3p**	CAACGAAAGGTTATCTTCCTGGAT	24	119	37.70	-59.30
**aca- miR879241-3p**	CGGAGGAAAAGAAACTAACA	20	96	42.40	-46
**aca- miR254217-3p**	CTCAGTCCTTGGGCTGGTTGCTCT	24	129	57.50	-101.20
**aca- miR5619838-5p**	GCCGCTGATAGAGGGCCTGCAACCT	25	146	65.10	-98.30
**aca- miR190576-3p**	CGAGCCATTCAAAGTCTGGACTTTG	25	95	46.90	-51.60

### Differentially expressed miRNAs in two different life stages of *A*. *catenella*

To identify miRNAs related to the growth of *A*. *catenella*, the normalized expression of miRNAs in the two libraries were compared. Twelve known miRNAs were expressed differently in the two phases (Table 4). Among them osa-miR2876-3p_R+1 was preferentially expressed in the lag phase and zma-miR529-5p was only expressed in the logarithmic phase. The other 10 miRNAs were expressed in both libraries. Compared with the lag phase cultures, four miRNAs were significantly down-regulated and eight miRNAs were up-regulated in the logarithmic phase algae. Of the12 sequences, the majority ranged from a 1- to 2-fold difference, and only four miRNAs showed greater than 2-fold differences between the two growth stages.

**Table 4:** Twelve differentially expressed miRNAs in two different growth phases in *A*. *catenella*.

miRNA name	log_2_ (fold change)	*P*value (chi square_2x2)	up/down
**osa-miR168a-5p**	-2.06	0.0044	down
**tae-miR159a**	-2.05	0.0202	down
**aca- miR43924-5p**	-1.11	0.0000	down
**zma-miR529-5p**	-∞	0.0452	down
**osa-miR2876-3p_R+1**	+∞	0.0027	up
**rgl-miR5139**	1.12	0.0060	up
**aca- miR456915-3p**	1.21	0.0003	up
**sbi-miR169c**	1.23	0.0294	up
**stu-miR169a-5p_R+1**	1.23	0.0294	up
**zma-miR169f-5p**	1.23	0.0294	up
**stu-miR171b-3p**	1.38	0.0248	up
**bdi-miR7732-3p_L-1_1ss11GC**	1.79	0.0055	up

log_2_ (fold change):log_2_ (the normalized expression in the lag phase /the normalized expression in the logarithmic phase); R+1 means the miRNA_seq (detected) is one base more than known rep_miRSeq on the right side; L-1 means the miRNA_seq (detected) is one base less than known rep_miRSeq on the left side; 1ss11GC means one substitution (ss), where G was substituted by C at position 11.

Some of the miRNA families were expressed solely in the lag phase or the logarithmic phase. For example, miR1862 (with one copy), miR1507, and miR947 were expressed exclusively in the lag stage, whereas miR535, miR820, and miR529 were only expressed in the logarithmic stage. These significant result ssuggested that these families may have potentially important effects on the initial and exponential growth stages, respectively.

**Fig 7:**
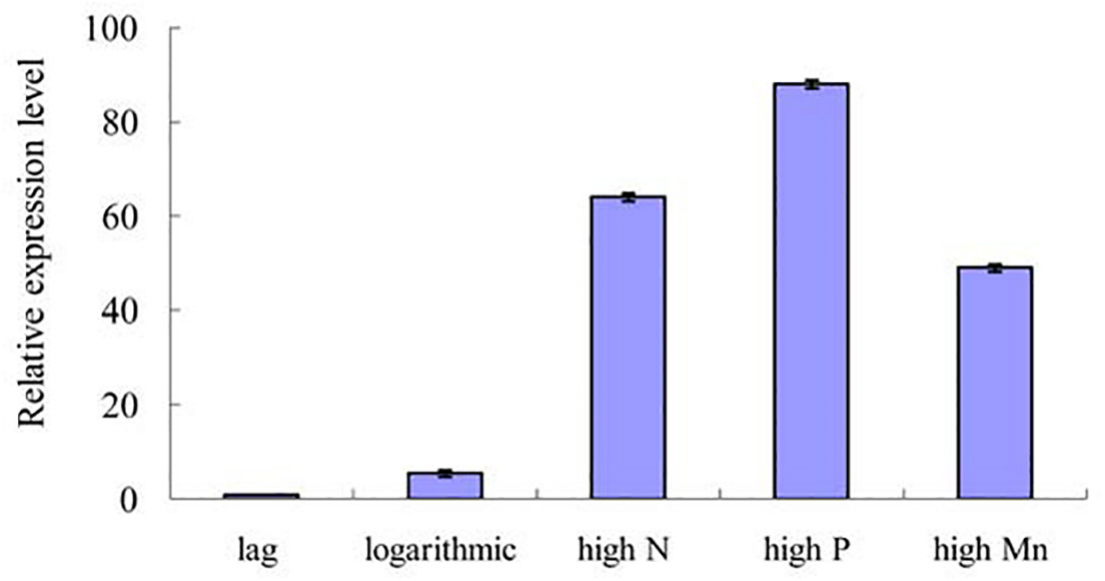
qPCR validation of the differentially expressedtae-miR159a.

**Fig 8:**
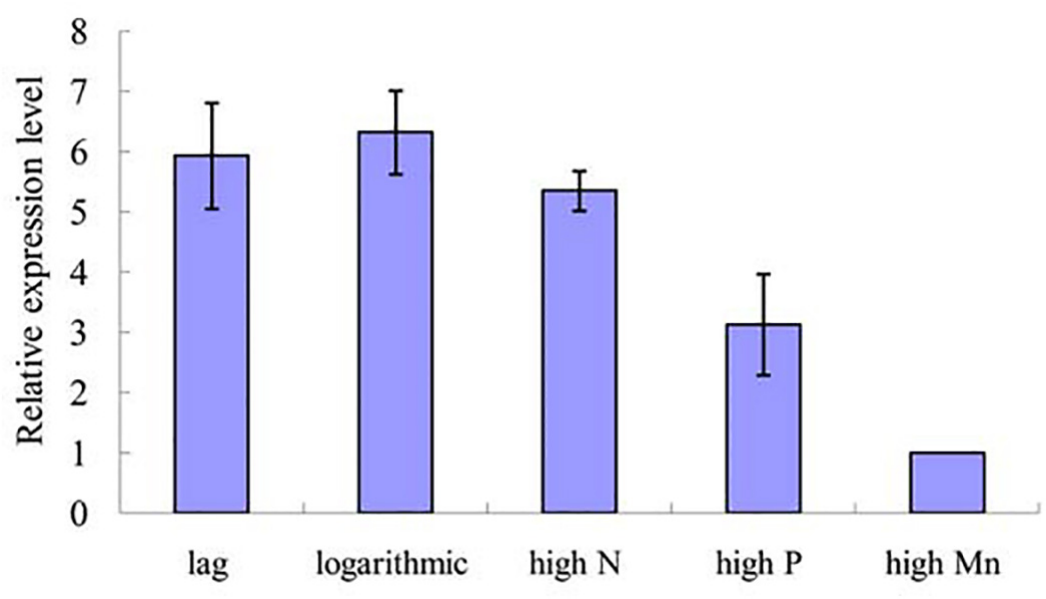
qPCR validation of the differentially expressedaca-miR-3p-456915. Normalized expression level (2^–ΔΔCT^) of tae-miR159a and aca-miR-3p-456915 under different growth phases and conditions.

qPCR was performed to confirm the expression of the miRNAs identified by the high-throughput sequence approach. The two selected miRNAs included one up-regulated novel miRNA(aca-miR-3p-456915) and one down-regulated conserved miRNA (tae-miR159a).Fig 7 illustrates the relative expression levels oftae-miR159a. Its expression in the logarithmic phase and in the logarithmic phases of the three nutrient-enhanced cultures were all significantly higher than those in the lag phase (*P*<0.01). The |log_2_fold change|oftae-miR159awas 2.05 as determined by high-throughput sequencing. The expression trend of the qPCR assay was generally consistent with the deep sequencing results. For aca-miR-3p-456915,expressionsin the high P and high Mn induced logarithmic phases were both significantly lower than those in the lag phase (*P*<0.05), whereas its expression in the logarithmic phase was slightly higher than that in the lag phase (Fig 8).

### Prediction of potential targets for differentially expressed miRNAs and function annotation

**Fig 9:**
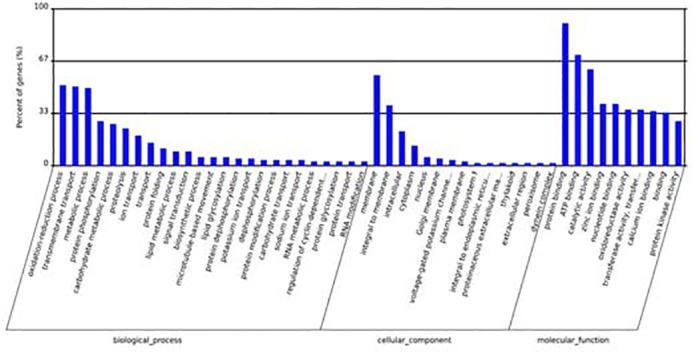
Partial gene ontology (GO) classification annotated for predicted target genes of 12 differentially expressed miRNAs. The figure shows partial GO enrichment of the 1813 predicted target genes in three GO ontologies: biological processes, cellular component and molecular function.

To understand the specific functions of the 12 differentially expressed miRNAs during the growth of *A*. *catenella*, potential target genes were predicted by integrating two software programs (TargetScan5.0 and miRanda3.3a), and1813 potential target genes were identified (S3 Table).GO enrichment and KEGG pathway annotation were then applied to these targets. On the basis of three GO ontologies, 656target genes were primarily classified into 412 enriched GO terms. Among these, 38 GO terms were significantly enriched. The following GO terms were the most significantly enriched:protein binding, ATP binding, catalytic activity, membrane in cellular component, and the oxidation-reduction process (Fig 9).

KEGG pathway analysis of all predicted potential targets was then performed to reveal the enriched metabolic pathways that were regulated by differentially expressed miRNAs in *A*. *catenella*. In total,175 KEGG pathways were identified, with 13 pathways significantly enriched (Table 5). Biosynthesis of unsaturated fatty acids was enriched extremely significantly (*P*<0.01). Other very significantly enriched pathways were the TGF-beta signaling pathway, phenylalanine, tyrosine, and tryptophan biosynthesis, the Ca^2+^ signaling pathway, and the Wnt signaling pathway. These results suggest that the genes targeted by differentially expressed miRNAs might be involved in the growth processes of *A*. *catenella*.

**Table 5:** The significantly enriched KEGG pathways of target genes for 12 differentially expressed miRNAs.

Pathway ID	Pathway	target gene number	*P*-value of Fisher's Exact Test
ko01040	Biosynthesis of unsaturated fatty acids	5	0.0037
ko04350	TGF-beta signaling pathway	4	0.0155
ko00400	Phenylalanine, tyrosine and tryptophan biosynthesis	4	0.0169
ko04020	Calcium signaling pathway	6	0.0225
ko04310	Wnt signaling pathway	6	0.0247
ko00592	alpha-Linolenic acid metabolism	2	0.0255
ko02040	Flagellar assembly	1	0.0255
ko04111	Cell cycle—yeast	8	0.0356
ko00260	Glycine, serine and threonine metabolism	7	0.0364
ko00380	Tryptophan metabolism	7	0.0378
ko05210	Colorectal cancer	3	0.0437
ko00630	Glyoxylate and dicarboxylate metabolism	6	0.0460
ko04910	Insulin signaling pathway	10	0.0486

## Discussion

### Sequence of small RNA libraries of *A*. *catenella*

The high-throughput Illumina deep sequencing platform offers a powerful approach for profiling small RNAs, and it is efficient for discovering miRNAs in various organisms. In the present study, two smallRNA cDNA libraries were constructed, and detailed miRNA profiles were obtained for the lag phase library and the logarithmic phase library using this method. Altogether, 38,092,056 and 32,969,156 unfiltered reads, respectively, representing 9,311,258 and 7,713,621unique reads were acquired. These results confirmed the existence of miRNAs in this unicellular dinoflagellate. These miRNAs also exhibited an unexpected complexity in the small RNA regulation mechanism of *A*. *catenella*.

To the best of our knowledge, this is the first report of comprehensive identification and expression profiling of miRNAs of *A*. *catenella* in different developmental life stages. The 24nt small RNA sequences were dominant, which is in accordance with data for typical higher plants such as *A*. *thaliana*[23],*A*. *hypogaea*[26],and *O*. *sativa*[27]. The 22nt sequences were next in dominance, followed by the25 and 23nt sequences. In *P*.*tricornutum*, small RNAs of 22nt were the most common[34], and the same was found for aquatic animals such as *Paralichthysolivaceus*[59]and *Cynoglossussemilaevis*[44]. In *S*.*microadriaticum*(a dinoflagellate symbiont of reef-building corals) [35]and *U*.*prolifera*[31], the most frequent read was 25nt;however, the 21nt sequences ranked first in the unicellular green alga *C*.*reinhardtii*[33]and the marine red alga *P*.*yezoensis*[32]. It has been reported that small RNAs of 24ntwereabsent in *C*.*reinhardtii*[33] and the plant *P*.*patens*[54],which is different from the scenario in many higher plants [23, 26, 27]. These reports suggested that small RNAs have multiple distribution patterns in various algal species.

The kinds of enzymes that participated in sample processing were responsible for the length of small RNAs. For instance, 24nt molecules can be generated by Dicer-like protein (DCL) DCL3 and 21nt small RNAs can be processed by DCL1, whereas in animals the 22nt fraction require the RNase III endonuclease Drosha [60–62]. In plants, the most abundant size are always 24 nt, while in animal kingdoms, that are 22 nt. In *A*. *catenella*, 24 nt size is the most abundant, followed by 22 nt. Therefore, these typical length distributions of sRNAs that found in the plant and animal kingdoms were unsuitable for *A*. *catenella*. The characteristic length distribution of small RNAs in *A*. *catenella* suggests that lower level unicellular algae might have different kinds of RNA processing enzymes when compared to the plants and animals. It is likely that both the typical DCL as found in plants and the classic Dicer as in animals are used to process miRNA in *A*. *catenella*, which is consistent with the dual animal and plant behavior of this alga [63].Previous studies have shown that the Dicer-like enzymes in *U*.*prolifera* have a sequence similar to that found in higher organisms [31]. Thus, studies are needed to investigate whether the enzymes in *A*. *catenella* are Dicer, DCL, or other enzymes that have sequence homology with them. Additionally more work is necessary to elucidate their exact roles.

### The roles of identified miRNAs in different growth stages

Eighty-eight miRNAs belonging to 32 miRNA families were identified and their expression profiles were further analyzed. Our results showed that the member number, the expression level of various families, and the expression level of each member within one family were significantly different. Two highly conserved miRNA families,miR167 and miR396, were abundant in both libraries.miR167 exerts a key role in roots and shoots by controlling the transcriptional response to auxin signals, including growth, division, and differentiation of cells of the plant [17]. A study of *A*.*thaliana* indicated that miR396 targeted transcription factor AtGRF, which participates in the regulation of cell division and differentiation in leaf development[26].Based on these results, we propose that highly expressed miR167 and miR396 may be related to the rapid growth of the algal cell.

### Targets of differentially expressed miRNAs

After prediction of the target genes of differentially expressed miRNAs, KEGG annotation of these potential targets was conducted to gain information about gene regulation during different growth periods in *A*. *catenella*. The differentially expressed miRNAs are likely related to growth of the unicellular alga.miR159appears to target MYB (v-myb avian myeloblastosis viral oncogene homolog) transcription factors, which have important roles in responding to hormones; this premise has been validated experimentally in previous study[64]. Another report on *Arabidopsis* indicated that the expression level of miR159can be enhanced by gibberellins [65].In the tomato plant, researchers reported that miR159 targeted 1-amino cyclopropane carboxylic acid (ACC) synthase, which plays crucial roles in fruit ripening[66]. In our study, miR159a targeted many functional enzymes that are crucial in multiple metabolic pathways, such as fatty acid synthase subunit beta, cysteine synthase A, cytochrome c oxidase cbb3-type subunit I, and various dehydrogenases and transferases. Due to the lack of MYB transcription factor genes and ACC synthase in the transcriptome that was referenced during target prediction, our results are not identical with those of other reports. Therefore, we suggested that159a might target different genes to ultimately regulate the growth of *A*. *catenella*. qPCR results indicated that the expression of miR159a was dramatically up-regulated in the exponential growth phase and the exponential phases of the three nutrient-enhanced cultures which were consistent with the deep sequencing results. (Fig 7). These results also provide evidence that high-throughput sequencing is a reliable method to identifying differentially expressed miRNAs indifferent growth phases of *A*. *catenella*. The expression of target genes is regulated negatively by miRNA. Therefore, we propose that miR159with higher expression levels in the logarithmic phase might play an important part in the rapid growth of *A*. *catenella* during red tide blooms.

Our results indicated that replication licensing factor (RLF) and the cell division cycle gene (cdc15)in the cell cycle were the target genes of miR171b. As a critical initiation factor, RLF combines with chromatin when the karyotheca ruptures at M phase so that the cell cycle can progress to S phase [67, 68].Previous research showed that RLF is involved in the precise replication during every single cell cycle by holding back re-replication of chromosomal DNA[69]. Meanwhile, the cdc gene codes a cyclin-dependent protein that directly takes part in cell division and regulation of the cell cycle[70].In our study, miR171b was up-regulated in the logarithmic phase; thus, we suggest that miR171b might carry out an important regulatory function in the rapid growth of *A*. *catenella* by controlling precise replication during the cell cycle and cell division.

In addition, miR169 (miR169a, miR169c, and miR169f) targeted calmodulin-dependent protein kinase II (CaMKII), which is an important member of the ubiquitous calcium ion(Ca^2+^) signaling pathway. Many reports have noted that CaMKII might be related to the cell cycle and rapid cellular metabolism[71]. To date, CaMK homologs have been successfully isolated from *O*.*sativa*, *Maluspumila*, *Liliumbrownie*and *Nicotianatabacum* [72–75]. In maize, two CaMK homologs of *MCK1*and *MCK2* were identified and validated experimentally to be accumulated, particularly in plant parts undergoing rapid growth such as the apical meristem, root cap, and flower primordium [76]. Similar results have been found in *O*. *sativa* [72].In animals, researchers have found that CaMKII can promote the proliferation and growth of the cell by facilitating the replication of the centriole. At the same time, G2 in the cell cycle can be arrested by the use of CaMKII inhibitor[77,71]. Therefore, we speculated that CaMKII might play a major role in regulating the cell cycle and the rapid growth of the logarithmic phase in single-celled dinoflagellates. In *A*. *catenella*, CaM was shown to activate CaMKII, which provided evidence of its regulatory function [78]. These results suggest that miR169, which was found to be up-regulated in the logarithmic phase, might have a significant role in the blooming growth of *A*. *catenella*. However, much more research is needed tov alidate the target genes of CaMKII and to elucidate how miR169exerts its effect on growth in dinoflagellates.

In this study,aca-miR-3p-456915 was one of the predicted potentially novel miRNAs that expressed differentially in the two different growth phases (down-regulated in the exponential phase), and this finding was further validated by qPCR (Fig 8). KEGG pathway annotation was conducted on the target genes of aca-miR-3p-456915. These target genes were mainly enriched in the metabolic processes related to DNA replication and cell division, such as biosynthesis of unsaturated fatty acids, mismatch repair, biosynthesis of purine and pyrimidine, metabolism of various amino acids, and oxidative phosphorylation. Reports have shown that the proteins that take part in DNA replication and tRNA synthesis had a higher expression level in the logarithmic phase compared to the lag phase in *A*. *catenella*[12]. Thus,aca-miR-3p-456915might affect the rapid growth of algal cells during the logarithmic phase by providing the materials and energy necessary for speedy proliferation of the cell.

## Conclusions

The present report is the first to identify and profile the expression pattern of miRNA in *A*. *catenella* through high-throughput sequencing. Eighty-eight conserved miRNAs belonging to 32 families and 15 novel miRNAs were found. Twelve miRNAs were identified that were differentially expressed in the lag phase and the logarithmic phase. Two of the differentially expressed miRNAs were verified by qPCR. Target predictions of the 12 differentially expressed miRNAs resulted in 1813 target genes. GO and KEGG annotation revealed highly enriched GO terms and metabolic pathways that were related to the growth of toxic *A*. *catenella*. miRNAs may play important roles in the rapid growth of *A*. *catenella* by the regulation of DNA replication and the cell cycle. These findings provide new insights into understanding the molecular mechanisms involved in regulation of algal cell growth.

## Supporting Information

S1 Fig Secondary structures of aca-miR43924-5p. (TIF)

S2 Fig Secondary structures of aca- miR4542336-3p. (TIF)

S3 Fig Secondary structures of aca- miR35336-5p. (TIF)

S4 Fig Secondary structures of aca- miR311414-3p. (TIF)

S5 Fig Secondary structures of aca- miR129519-3p-1. (TIF)

S6 Fig Secondary structures of aca- miR129519-3p-2. (TIF)

S7 Fig Secondary structures of aca- miR312912-5p. (TIF)

S8 Fig Secondary structures of aca- miR5257085-3p. (TIF)

S9 Fig Secondary structures of aca- miR456915-3p. (TIF)

S10 Fig Secondary structures of aca- miR342562-5p. (TIF)

S11 Fig Secondary structures of aca- miR126213-3p. (TIF)

S12 Fig Secondary structures of aca- miR879241-3p. (TIF)

S13 Fig Secondary structures of aca- miR254217-3p. (TIF)

S14 Fig Secondary structures of aca- miR5619838-5p. (TIF)

S15 Fig Secondary structures of aca- miR190576-3p. (TIF)

S1 Table Species range and species priority information. All the aligned species in the miRBase and their priority are listed. *Chlamydomonas reinhardii* (cre) was the preferentially aligned species.
(DOCX)

S2 Table Specific forward primers used in this experiment. The specific forward primersof 5.8s, aca-miR-3p-456915 and tae-miR159a are listed.
(DOCX)

S3 Table The results of predicted target genesof 12 differentially expressed miRNAsin *A*. *catenella*. In total, 1813 target genes were predicted.
(DOCX)
