# Supplementary material for: Identification of microRNAs in the Toxigenic Dinoflagellate Alexandrium catenella by High-Throughput Illumina Sequencing and Bioinformatic Analysis
Source: PLoS One. 2015 Sep 23;10(9):e0138709. doi: 10.1371/journal.pone.0138709 (PMC4580472; doi:10.1371/journal.pone.0138709)
Supplement: S1 Table — All the aligned species in the miRBase and their priority are listed. Chlamydomonas reinhardii (cre) was the preferentially aligned species. (DOCX) [file pone.0138709.s016.docx]

**S1 Table Species range and species priority information**

| **Phylum** | **Class** | **Organism** | **Common name** | **3 letter name** | **Species Priority** |
| --- | --- | --- | --- | --- | --- |
| Chlorophyta | Chlorophyceae | *Chlamydomonas reinhardtii* | Algae | cre | preferential species |
| Bacillariophyta | pennates | *Phaeodactylum tricornutum* | Diatom | pti | other selected species |
| Magnoliophyta | eudicotyledons | *Arabidopsis lyrata* | Arabidopsis lyrata | aly | other selected species |
| Magnoliophyta | eudicotyledons | *Arabidopsis thaliana* | Arabidopsis thaliana | ath | other selected species |
| Magnoliophyta | monocotyledons | *Oryza sativa* | Rice | osa | other selected species |
| Magnoliophyta | eudicotyledons | *Glycine max* | Soybean | gma | other selected species |
| Magnoliophyta | monocotyledons | *Zea mays* | Corn | zma | other selected species |
| Magnoliophyta | monocotyledons | *Hordeum vulgare* | Common barley | hvu | other selected species |
| Magnoliophyta | monocotyledons | *Triticum aestivum* | Common wheat | tae | other selected species |
| Magnoliophyta | eudicotyledons | *Glycine soja* | Wild soybean | gso | other selected species |
| Embryophyta | bryopsida | *Physcomitrella patens* | Physcomitrella patens | ppt | other selected species |
| Embryophyta | lycopodiatae | *Selaginella moellendorffii* | S. moellendorffii | smo | other selected species |
| Magnoliophyta | eudicotyledons | *Medicago truncatula* | M. truncatula | mtr | other selected species |
| Magnoliophyta | eudicotyledons | *Populus trichocarpa* | Cottonwood | ptc | other selected species |
| Magnoliophyta | monocotyledons | *Brachypodium distachyon* | Purple false brome | bdi | other selected species |
| Magnoliophyta | eudicotyledons | *Solanum tuberosum* | Irish potato | stu | other selected species |
| Magnoliophyta | eudicotyledons | *Malus domestica* | Apple | mdm | other selected species |
| Magnoliophyta | monocotyledons | *Saccharum officinarum* | Saccharum officinarum | sof | other selected species |
| Magnoliophyta | eudicotyledons | *Vitis vinifera* | Grape | vvi | other selected species |
| Magnoliophyta | eudicotyledons | *Nicotiana tabacum* | Cultivated tobacco | nta | other selected species |
| Magnoliophyta | eudicotyledons | *Manihot esculenta* | Cassava | mes | other selected species |
| Magnoliophyta | eudicotyledons | *Linum usitatissimum* | Common flax | lus | other selected species |
| Magnoliophyta | eudicotyledons | *Cucumis melo* | Cucumis melo | cme | other selected species |
| Magnoliophyta | eudicotyledons | *Brassica napus* | Oilseed rape | bna | other selected species |
| Magnoliophyta | eudicotyledons | *Theobroma cacao* | cocoa tree | tcc | other selected species |
| Magnoliophyta | eudicotyledons | *Carica papaya* | Carica papaya | cpa | other selected species |
| Magnoliophyta | eudicotyledons | *Gossypium hirsutum* | Mexican cotton | ghr | other selected species |
| Magnoliophyta | eudicotyledons | *Ricinus communis* | Castor oil plant | rco | other selected species |
| Magnoliophyta | eudicotyledons | *Lotus japonicus* | Lotus japonicus | lja | other selected species |
| Magnoliophyta | eudicotyledons | *Cynara cardunculus* | Cynara cardunculus | cca | other selected species |
| Magnoliophyta | eudicotyledons | *Solanum lycopersicum* | Tomato | sly | other selected species |
| Magnoliophyta | eudicotyledons | *Aquilegia caerulea* | Aquilegia coerulea | aqc | other selected species |
| Coniferophyta | pinopsida | *Picea abies* | Picea abies | pab | other selected species |
| Magnoliophyta | eudicotyledons | *Brassica rapa* | Turnip | bra | other selected species |
| Coniferophyta | pinopsida | *Pinus taeda* | Loblolly pine | pta | other selected species |
| Coniferophyta | pinopsida | *Pinus densata* | Sikang Pine | pde | other selected species |
| Magnoliophyta | monocotyledons | *Sorghum bicolor* | Sorghum | sbi | other selected species |
| Magnoliophyta | eudicotyledons | *Vigna unguiculata* | Vigna unguiculata | vun | other selected species |
| Magnoliophyta | eudicotyledons | *Salvia sclarea* | Salvia sclarea | ssl | other selected species |
| Magnoliophyta | eudicotyledons | *Helianthus tuberosus* | Jerusalem artichoke | htu | other selected species |
| Magnoliophyta | monocotyledons | *Saccharum ssp.* | Saccharum spp. | ssp | other selected species |
| Magnoliophyta | monocotyledons | *Festuca arundinacea* | Tall fescue | far | other selected species |
| Magnoliophyta | eudicotyledons | *Digitalis purpurea* | common foxglove | dpr | other selected species |
| Magnoliophyta | eudicotyledons | *Rehmannia glutinosa* | Rehmannia glutinosa | rgl | other selected species |
| Magnoliophyta | eudicotyledons | *Phaseolus vulgaris* | Common bean | pvu | other selected species |
| Magnoliophyta | eudicotyledons | *Helianthus annuus* | Sunflower | han | other selected species |
