# Supplementary material for: Identification of microRNAs in the Toxigenic Dinoflagellate Alexandrium catenella by High-Throughput Illumina Sequencing and Bioinformatic Analysis
Source: PLoS One. 2015 Sep 23;10(9):e0138709. doi: 10.1371/journal.pone.0138709 (PMC4580472; doi:10.1371/journal.pone.0138709)
Supplement: S2 Table — The specific forward primersof 5.8s, aca-miR-3p-456915 and tae-miR159a are listed. (DOCX) [file pone.0138709.s017.docx]

**S2 Table Specific forward primer used in this experiment**

| Name | Sequence 5’→3’ | Application | Length(bp) |
| --- | --- | --- | --- |
| 5.8srRNA | GCTAATGATATTGTGGGCGGTGTA | 5.8srRNA cloning and qPCR  miR456915 cloning and qPCR | 24 bp  23bp |
| aca-miR-3p-456915 | CAAAATGGGCGGCAAGAAAGGCT |  |  |
| tae-miR159a | TTTGGATTGAAGGGAGCTCTG | miR159a cloning and qPCR | 21 bp |
