# Supplementary material for: Identification of microRNAs in the Toxigenic Dinoflagellate Alexandrium catenella by High-Throughput Illumina Sequencing and Bioinformatic Analysis
Source: PLoS One. 2015 Sep 23;10(9):e0138709. doi: 10.1371/journal.pone.0138709 (PMC4580472; doi:10.1371/journal.pone.0138709)
Supplement: S3 Table — In total, 1813 target genes were predicted. (DOCX) [file pone.0138709.s018.docx]

|  | **S3 Table The results of predicted target genes of 12 differentially expressed miRNAs in *A. catenella.*** |  |
| --- | --- | --- |
| Transcript ID | Gene Annoation | miRNA ID |
| Algae_064-1_Unigene_BMK.10015 | Vegetative cell wall protein gp1 OS=Chlamydomonas reinhardtii GN=GP1 PE=2 SV=1 | stu-miR171b-3p |
| Algae_064-1_Unigene_BMK.10030 |  | aca-miR-3p-456915 |
| Algae_064-1_Unigene_BMK.10126 | Eukaryotic peptide chain release factor subunit 1 OS=Drosophila melanogaster GN=eRF1 PE=1 SV=2 | zma-miR529-5p |
| Algae_064-1_Unigene_BMK.10275 | Mitogen-activated protein kinase 6 OS=Arabidopsis thaliana GN=MPK6 PE=1 SV=1 | aca-miR-3p-456915 |
| Algae_064-1_Unigene_BMK.10518 |  | osa-miR168a-5p |
| Algae_064-1_Unigene_BMK.10582 | Translation initiation factor IF-2 OS=Synechococcus sp. (strain CC9902) GN=infB PE=3 SV=1 | sbi-miR169c |
| Algae_064-1_Unigene_BMK.10634 | Beta-galactoside alpha-2,6-sialyltransferase 2 OS=Bos taurus GN=ST6GAL2 PE=2 SV=1 | osa-miR2876-3p_R+1 |
| Algae_064-1_Unigene_BMK.10647 |  | osa-miR168a-5p |
| Algae_064-1_Unigene_BMK.10665 |  | sbi-miR169c |
| Algae_064-1_Unigene_BMK.10753 | Putative sulfur deprivation response regulator OS=Chlamydomonas reinhardtii GN=SAC1 PE=2 SV=1 | aca-miR-5p-43924 |
| Algae_064-1_Unigene_BMK.10759 | Renin OS=Callithrix jacchus GN=REN PE=2 SV=1 | tae-miR159a |
| Algae_064-1_Unigene_BMK.10835 | Mucin-19 OS=Mus musculus GN=Muc19 PE=2 SV=2 | zma-miR529-5p |
| Algae_064-1_Unigene_BMK.10920 | Aflatoxin B1 aldehyde reductase member 2 OS=Mus musculus GN=Akr7a2 PE=1 SV=2 | sbi-miR169c |
| Algae_064-1_Unigene_BMK.10927 | Putative protein TPRXL OS=Homo sapiens GN=TPRXL PE=5 SV=2 | stu-miR171b-3p |
| Algae_064-1_Unigene_BMK.11258 | Protein phosphatase 1G OS=Homo sapiens GN=PPM1G PE=1 SV=1 | osa-miR168a-5p |
| Algae_064-1_Unigene_BMK.11271 | Carbonic anhydrase, chloroplastic OS=Hordeum vulgare PE=2 SV=1 | aca-miR-3p-456915 |
| Algae_064-1_Unigene_BMK.11279 | Translation initiation factor IF-2 OS=Frankia sp. (strain EAN1pec) GN=infB PE=3 SV=1 | sbi-miR169c |
| Algae_064-1_Unigene_BMK.11338 | Serine/arginine repetitive matrix protein 2 OS=Mus musculus GN=Srrm2 PE=1 SV=2 | zma-miR529-5p |
| Algae_064-1_Unigene_BMK.11393 | Uncharacterized protein LOC284861 OS=Homo sapiens PE=2 SV=1 | osa-miR168a-5p |
| Algae_064-1_Unigene_BMK.11403 | Dapper homolog 3 OS=Mus musculus GN=Dact3 PE=2 SV=1 | aca-miR-3p-456915 |
| Algae_064-1_Unigene_BMK.11471 | DNA replication licensing factor mcm6 OS=Schizosaccharomyces pombe GN=mcm6 PE=1 SV=2 | stu-miR171b-3p |
| Algae_064-1_Unigene_BMK.11502 | FKBP-type peptidyl-prolyl cis-trans isomerase OS=Methanococcus thermolithotrophicus PE=1 SV=1 | zma-miR529-5p |
| Algae_064-1_Unigene_BMK.11625 | Probable glucan 1,3-beta-glucosidase A OS=Neosartorya fumigata (strain ATCC MYA-4609 / Af293 / CBS 101355 / FGSC A1100) GN=exgA PE=3 SV=1 | stu-miR171b-3p |
| Algae_064-1_Unigene_BMK.11707 | Serine/arginine repetitive matrix protein 2 OS=Mus musculus GN=Srrm2 PE=1 SV=2 | zma-miR529-5p |
| Algae_064-1_Unigene_BMK.11751 | Werner Syndrome-like exonuclease OS=Arabidopsis thaliana GN=WEX PE=1 SV=1 | aca-miR-3p-456915 |
| Algae_064-1_Unigene_BMK.11967 | Putative protein TPRXL OS=Homo sapiens GN=TPRXL PE=5 SV=2 | aca-miR-5p-43924 |
| Algae_064-1_Unigene_BMK.11974 | Crt homolog 1 OS=Dictyostelium discoideum GN=crtp1 PE=2 SV=1 | aca-miR-5p-43924 |
| Algae_064-1_Unigene_BMK.1200 | ATP-binding cassette sub-family G member 1 OS=Homo sapiens GN=ABCG1 PE=2 SV=3 | osa-miR168a-5p |
| Algae_064-1_Unigene_BMK.12065 | Leucine-rich repeat-containing protein 1 OS=Homo sapiens GN=LRRC1 PE=1 SV=1 | bdi-miR7732-3p_L-1_1ss11GC |
| Algae_064-1_Unigene_BMK.12178 | DNL-type zinc finger protein OS=Danio rerio GN=dnlz PE=2 SV=1 | osa-miR168a-5p |
| Algae_064-1_Unigene_BMK.12575 |  | aca-miR-5p-43924 |
| Algae_064-1_Unigene_BMK.12600 | Putative protein TPRXL OS=Homo sapiens GN=TPRXL PE=5 SV=2 | tae-miR159a |
| Algae_064-1_Unigene_BMK.12611 | Serine/arginine repetitive matrix protein 2 OS=Mus musculus GN=Srrm2 PE=1 SV=2 | sbi-miR169c |
| Algae_064-1_Unigene_BMK.12631 | Squalene synthase OS=Candida albicans GN=ERG9 PE=3 SV=1 | aca-miR-5p-43924 |
| Algae_064-1_Unigene_BMK.12640 | Uncharacterized protein C24B11.05 OS=Schizosaccharomyces pombe GN=SPAC24B11.05 PE=2 SV=1 | sbi-miR169c |
| Algae_064-1_Unigene_BMK.12698 | NAD(P) transhydrogenase, mitochondrial OS=Mus musculus GN=Nnt PE=1 SV=2 | stu-miR171b-3p |
| Algae_064-1_Unigene_BMK.12832 | Basic proline-rich protein OS=Sus scrofa PE=1 SV=2 | osa-miR168a-5p |
| Algae_064-1_Unigene_BMK.1288 |  | zma-miR529-5p |
| Algae_064-1_Unigene_BMK.12905 | Arsenite methyltransferase OS=Mus musculus GN=As3mt PE=2 SV=1 | sbi-miR169c |
| Algae_064-1_Unigene_BMK.12932 | Neural Wiskott-Aldrich syndrome protein OS=Homo sapiens GN=WASL PE=1 SV=2 | sbi-miR169c |
| Algae_064-1_Unigene_BMK.13037 | Uncharacterized protein BHLF1 OS=Epstein-Barr virus (strain B95-8) GN=BHLF1 PE=4 SV=1 | sbi-miR169c |
| Algae_064-1_Unigene_BMK.13075 | Tryptophanyl-tRNA synthetase OS=Thermosynechococcus elongatus (strain BP-1) GN=trpS PE=3 SV=1 | aca-miR-5p-43924 |
| Algae_064-1_Unigene_BMK.13112 | Translation initiation factor IF-2 OS=Kineococcus radiotolerans (strain ATCC BAA-149 / DSM 14245 / SRS30216) GN=infB PE=3 SV=1 | bdi-miR7732-3p_L-1_1ss11GC |
| Algae_064-1_Unigene_BMK.13451 |  | aca-miR-5p-43924 |
| Algae_064-1_Unigene_BMK.13554 | Neurotrypsin OS=Pan troglodytes GN=PRSS12 PE=3 SV=1 | sbi-miR169c |
| Algae_064-1_Unigene_BMK.13686 | Uncharacterized serine/threonine-rich protein PB15E9.01c OS=Schizosaccharomyces pombe GN=SPAPB15E9.01c PE=2 SV=2 | sbi-miR169c |
| Algae_064-1_Unigene_BMK.13749 |  | aca-miR-3p-456915 |
| Algae_064-1_Unigene_BMK.13758 |  | aca-miR-5p-43924 |
| Algae_064-1_Unigene_BMK.14038 | Serine/arginine repetitive matrix protein 2 OS=Homo sapiens GN=SRRM2 PE=1 SV=2 | zma-miR529-5p |
| Algae_064-1_Unigene_BMK.14126 |  | sbi-miR169c |
| Algae_064-1_Unigene_BMK.14308 |  | aca-miR-3p-456915 |
| Algae_064-1_Unigene_BMK.14309 | Axoneme-associated protein mst101(2) OS=Drosophila hydei GN=mst101(2) PE=1 SV=1 | stu-miR171b-3p |
| Algae_064-1_Unigene_BMK.14342 | Isocitrate lyase OS=Cucurbita maxima PE=2 SV=1 | aca-miR-5p-43924 |
| Algae_064-1_Unigene_BMK.14414 | Viral protein TPX OS=Thermoproteus tenax virus 1 (strain VT3) PE=4 SV=1 | sbi-miR169c |
| Algae_064-1_Unigene_BMK.14417 | Kynureninase OS=Monosiga brevicollis GN=kynu PE=3 SV=1 | aca-miR-5p-43924 |
| Algae_064-1_Unigene_BMK.14444 | Mucin-19 OS=Mus musculus GN=Muc19 PE=2 SV=2 | tae-miR159a |
| Algae_064-1_Unigene_BMK.14481 |  | sbi-miR169c |
| Algae_064-1_Unigene_BMK.14576 | Uncharacterized protein DDB_G0271670 OS=Dictyostelium discoideum GN=DDB_G0271670 PE=4 SV=1 | bdi-miR7732-3p_L-1_1ss11GC |
| Algae_064-1_Unigene_BMK.14579 |  | zma-miR529-5p |
| Algae_064-1_Unigene_BMK.14634 | Translation initiation factor IF-2 OS=Rhodococcus opacus (strain B4) GN=infB PE=3 SV=1 | sbi-miR169c |
| Algae_064-1_Unigene_BMK.14816 | Alanyl-tRNA synthetase OS=Pyrococcus kodakaraensis (strain ATCC BAA-918 / JCM 12380 / KOD1) GN=alaS PE=3 SV=1 | sbi-miR169c |
| Algae_064-1_Unigene_BMK.14972 | UPF0317 protein KRH_21160 OS=Kocuria rhizophila (strain ATCC 9341 / DSM 348 / NBRC 103217 / DC2201) GN=KRH_21160 PE=3 SV=1 | osa-miR2876-3p_R+1 |
| Algae_064-1_Unigene_BMK.1508 | Uncharacterized protein DDB_G0271670 OS=Dictyostelium discoideum GN=DDB_G0271670 PE=4 SV=1 | bdi-miR7732-3p_L-1_1ss11GC |
| Algae_064-1_Unigene_BMK.15111 | Sodium channel protein type 11 subunit alpha OS=Mus musculus GN=Scn11a PE=1 SV=1 | sbi-miR169c |
| Algae_064-1_Unigene_BMK.15135 | Voltage-dependent T-type calcium channel subunit alpha-1G OS=Rattus norvegicus GN=Cacna1g PE=2 SV=2 | aca-miR-5p-43924 |
| Algae_064-1_Unigene_BMK.15138 | Uncharacterized protein BHLF1 OS=Epstein-Barr virus (strain B95-8) GN=BHLF1 PE=4 SV=1 | rgl-miR5139_L+3 |
| Algae_064-1_Unigene_BMK.15202 | Translation initiation factor IF-2 OS=Frankia sp. (strain CcI3) GN=infB PE=3 SV=1 | aca-miR-5p-43924 |
| Algae_064-1_Unigene_BMK.15563 | Uncharacterized protein BHLF1 OS=Epstein-Barr virus (strain B95-8) GN=BHLF1 PE=4 SV=1 | aca-miR-3p-456915 |
| Algae_064-1_Unigene_BMK.15586 | Immediate-early protein IE180 OS=Suid herpesvirus 1 (strain Kaplan) GN=IE PE=3 SV=1 | sbi-miR169c |
| Algae_064-1_Unigene_BMK.15859 | Putative sugar phosphate isomerase ywlF OS=Bacillus subtilis GN=ywlF PE=2 SV=1 | zma-miR529-5p |
| Algae_064-1_Unigene_BMK.16265 |  | bdi-miR7732-3p_L-1_1ss11GC |
| Algae_064-1_Unigene_BMK.1644 | Uncharacterized oxidoreductase YtbE OS=Bacillus subtilis GN=ytbE PE=1 SV=1 | stu-miR171b-3p |
| Algae_064-1_Unigene_BMK.16591 | Translation initiation factor IF-2 OS=Frankia sp. (strain CcI3) GN=infB PE=3 SV=1 | bdi-miR7732-3p_L-1_1ss11GC |
| Algae_064-1_Unigene_BMK.16636 | Formin-like protein 5 OS=Oryza sativa subsp. japonica GN=FH5 PE=2 SV=2 | zma-miR529-5p |
| Algae_064-1_Unigene_BMK.167 |  | stu-miR171b-3p |
| Algae_064-1_Unigene_BMK.16771 | Spore coat protein SP96 OS=Dictyostelium discoideum GN=cotA PE=4 SV=2 | aca-miR-5p-43924 |
| Algae_064-1_Unigene_BMK.16787 | Alpha-ketoglutarate-dependent dioxygenase AlkB homolog OS=Caulobacter crescentus GN=alkB PE=3 SV=1 | aca-miR-3p-456915 |
| Algae_064-1_Unigene_BMK.16815 | Geranylgeranyl diphosphate reductase, chloroplastic OS=Oryza sativa subsp. japonica GN=CHLP PE=2 SV=1 | osa-miR168a-5p |
| Algae_064-1_Unigene_BMK.16924 | Basic proline-rich protein OS=Sus scrofa PE=1 SV=2 | aca-miR-3p-456915 |
| Algae_064-1_Unigene_BMK.17012 | Serine/threonine-protein kinase SRK2F OS=Arabidopsis thaliana GN=SRK2F PE=1 SV=1 | aca-miR-3p-456915 |
| Algae_064-1_Unigene_BMK.17065 |  | bdi-miR7732-3p_L-1_1ss11GC |
| Algae_064-1_Unigene_BMK.1717 | Spore coat protein SP96 OS=Dictyostelium discoideum GN=cotA PE=4 SV=2 | zma-miR529-5p |
| Algae_064-1_Unigene_BMK.17350 | Translation initiation factor IF-2 OS=Streptomyces avermitilis GN=infB PE=3 SV=1 | aca-miR-5p-43924 |
| Algae_064-1_Unigene_BMK.17460 |  | sbi-miR169c |
| Algae_064-1_Unigene_BMK.176 | Uncharacterized protein DKFZp434B061 OS=Homo sapiens PE=2 SV=2 | rgl-miR5139_L+3 |
| Algae_064-1_Unigene_BMK.1774 | Serine/threonine-protein kinase Nek1 OS=Homo sapiens GN=NEK1 PE=1 SV=2 | osa-miR2876-3p_R+1 |
| Algae_064-1_Unigene_BMK.17878 |  | zma-miR529-5p |
| Algae_064-1_Unigene_BMK.17899 | Troponin C, skeletal muscle OS=Anguilla anguilla PE=1 SV=1 | sbi-miR169c |
| Algae_064-1_Unigene_BMK.17921 |  | sbi-miR169c |
| Algae_064-1_Unigene_BMK.17934 |  | zma-miR529-5p |
| Algae_064-1_Unigene_BMK.1794 | Serine/arginine repetitive matrix protein 2 OS=Mus musculus GN=Srrm2 PE=1 SV=2 | stu-miR171b-3p |
| Algae_064-1_Unigene_BMK.18008 | GTP-binding protein TypA/BipA homolog OS=Synechocystis sp. (strain ATCC 27184 / PCC 6803 / N-1) GN=typA PE=3 SV=1 | aca-miR-3p-456915 |
| Algae_064-1_Unigene_BMK.18021 | Dapper homolog 3 OS=Mus musculus GN=Dact3 PE=2 SV=1 | sbi-miR169c |
| Algae_064-1_Unigene_BMK.18395 | Serine/arginine repetitive matrix protein 1 OS=Homo sapiens GN=SRRM1 PE=1 SV=2 | aca-miR-5p-43924 |
| Algae_064-1_Unigene_BMK.18460 | Formin-like protein 18 OS=Oryza sativa subsp. japonica GN=FH18 PE=2 SV=1 | aca-miR-5p-43924 |
| Algae_064-1_Unigene_BMK.18476 |  | aca-miR-5p-43924 |
| Algae_064-1_Unigene_BMK.18601 | 40S ribosomal protein S12 OS=Cyanophora paradoxa GN=RPS12 PE=2 SV=1 | osa-miR168a-5p |
| Algae_064-1_Unigene_BMK.18754 | Retinoid isomerohydrolase OS=Homo sapiens GN=RPE65 PE=1 SV=3 | sbi-miR169c |
| Algae_064-1_Unigene_BMK.1880 | cGMP-dependent protein kinase 1 OS=Oryctolagus cuniculus GN=PRKG1 PE=2 SV=3 | aca-miR-3p-456915 |
| Algae_064-1_Unigene_BMK.19095 | Nuclear pore complex protein Nup98-Nup96 OS=Dictyostelium discoideum GN=nup98 PE=3 SV=1 | tae-miR159a |
| Algae_064-1_Unigene_BMK.1910 | Macrolide glycosyltransferase OS=Streptomyces lividans GN=mgt PE=3 SV=1 | osa-miR168a-5p |
| Algae_064-1_Unigene_BMK.19186 | Ubiquitin-conjugating enzyme E2 2 OS=Caenorhabditis elegans GN=let-70 PE=1 SV=1 | sbi-miR169c |
| Algae_064-1_Unigene_BMK.19278 | Translation initiation factor IF-2 OS=Frankia alni (strain ACN14a) GN=infB PE=3 SV=1 | sbi-miR169c |
| Algae_064-1_Unigene_BMK.19358 | Eukaryotic translation initiation factor 4E OS=Saccharomyces cerevisiae GN=CDC33 PE=1 SV=1 | osa-miR168a-5p |
| Algae_064-1_Unigene_BMK.1966 | Uncharacterized protein LOC284861 OS=Homo sapiens PE=2 SV=1 | aca-miR-5p-43924 |
| Algae_064-1_Unigene_BMK.19666 | Translation initiation factor IF-2 OS=Frankia alni (strain ACN14a) GN=infB PE=3 SV=1 | sbi-miR169c |
| Algae_064-1_Unigene_BMK.19801 | Guanine deaminase OS=Bacillus subtilis GN=guaD PE=1 SV=1 | zma-miR529-5p |
| Algae_064-1_Unigene_BMK.20043 | UPF0544 protein C5orf45 OS=Homo sapiens GN=C5orf45 PE=2 SV=2 | osa-miR168a-5p |
| Algae_064-1_Unigene_BMK.20051 | Serine/arginine repetitive matrix protein 2 OS=Homo sapiens GN=SRRM2 PE=1 SV=2 | sbi-miR169c |
| Algae_064-1_Unigene_BMK.2014 | Protein dodo OS=Drosophila melanogaster GN=dod PE=1 SV=3 | stu-miR171b-3p |
| Algae_064-1_Unigene_BMK.2026 |  | osa-miR2876-3p_R+1 |
| Algae_064-1_Unigene_BMK.2046 | Heme oxygenase 1 OS=Nostoc sp. (strain PCC 7120 / UTEX 2576) GN=pbsA1 PE=3 SV=1 | aca-miR-3p-456915 |
| Algae_064-1_Unigene_BMK.20584 |  | rgl-miR5139_L+3 |
| Algae_064-1_Unigene_BMK.20610 | Translation initiation factor IF-2 OS=Streptomyces griseus subsp. griseus (strain JCM 4626 / NBRC 13350) GN=infB PE=3 SV=1 | stu-miR171b-3p |
| Algae_064-1_Unigene_BMK.20625 | Serine/arginine repetitive matrix protein 2 OS=Homo sapiens GN=SRRM2 PE=1 SV=2 | aca-miR-3p-456915 |
| Algae_064-1_Unigene_BMK.20788 | Basic proline-rich protein OS=Sus scrofa PE=1 SV=2 | aca-miR-3p-456915 |
| Algae_064-1_Unigene_BMK.20801 | Ras-related protein Rab-8A OS=Gallus gallus GN=RAB8A PE=2 SV=1 | osa-miR168a-5p |
| Algae_064-1_Unigene_BMK.20851 | ATP-dependent zinc metalloprotease FtsH OS=Porphyra purpurea GN=ftsH PE=3 SV=1 | aca-miR-5p-43924 |
| Algae_064-1_Unigene_BMK.20970 |  | stu-miR171b-3p |
| Algae_064-1_Unigene_BMK.21343 |  | sbi-miR169c |
| Algae_064-1_Unigene_BMK.21519 | La-related protein OS=Drosophila melanogaster GN=larp PE=1 SV=4 | aca-miR-5p-43924 |
| Algae_064-1_Unigene_BMK.21747 | Peridinin-chlorophyll a-binding protein, chloroplastic OS=Symbiodinium sp. PE=1 SV=1 | sbi-miR169c |
| Algae_064-1_Unigene_BMK.21755 |  | aca-miR-3p-456915 |
| Algae_064-1_Unigene_BMK.21844 | TVP38/TMEM64 family membrane protein slr0305 OS=Synechocystis sp. (strain ATCC 27184 / PCC 6803 / N-1) GN=slr0305 PE=3 SV=1 | aca-miR-5p-43924 |
| Algae_064-1_Unigene_BMK.22005 | SH3 domain-binding protein 5-like OS=Pongo abelii GN=SH3BP5L PE=2 SV=1 | aca-miR-3p-456915 |
| Algae_064-1_Unigene_BMK.22102 | Vesicle-associated membrane protein-associated protein B OS=Bos taurus GN=VAPB PE=2 SV=1 | aca-miR-5p-43924 |
| Algae_064-1_Unigene_BMK.22129 |  | zma-miR529-5p |
| Algae_064-1_Unigene_BMK.22466 |  | aca-miR-5p-43924 |
| Algae_064-1_Unigene_BMK.22483 | Tetracycline resistance protein, class A OS=Escherichia coli GN=tetA PE=3 SV=2 | zma-miR529-5p |
| Algae_064-1_Unigene_BMK.22871 | Serine/arginine repetitive matrix protein 2 OS=Homo sapiens GN=SRRM2 PE=1 SV=2 | aca-miR-5p-43924 |
| Algae_064-1_Unigene_BMK.22890 |  | aca-miR-3p-456915 |
| Algae_064-1_Unigene_BMK.22939 |  | aca-miR-5p-43924 |
| Algae_064-1_Unigene_BMK.2321 |  | osa-miR2876-3p_R+1 |
| Algae_064-1_Unigene_BMK.23383 |  | aca-miR-5p-43924 |
| Algae_064-1_Unigene_BMK.23785 | Ribokinase OS=Homo sapiens GN=RBKS PE=1 SV=1 | aca-miR-3p-456915 |
| Algae_064-1_Unigene_BMK.23797 |  | zma-miR529-5p |
| Algae_064-1_Unigene_BMK.23894 | Uncharacterized protein LOC284861 OS=Homo sapiens PE=2 SV=1 | osa-miR168a-5p |
| Algae_064-1_Unigene_BMK.2391 | Extended synaptotagmin-3 OS=Xenopus tropicalis GN=esyt3 PE=2 SV=1 | aca-miR-5p-43924 |
| Algae_064-1_Unigene_BMK.24006 |  | bdi-miR7732-3p_L-1_1ss11GC |
| Algae_064-1_Unigene_BMK.24017 | Translation initiation factor IF-2 OS=Synechococcus sp. (strain CC9605) GN=infB PE=3 SV=1 | osa-miR168a-5p |
| Algae_064-1_Unigene_BMK.24161 | ATP-binding cassette sub-family B member 9 OS=Mus musculus GN=Abcb9 PE=2 SV=1 | rgl-miR5139_L+3 |
| Algae_064-1_Unigene_BMK.24382 | Putative protein TPRXL OS=Homo sapiens GN=TPRXL PE=5 SV=2 | aca-miR-5p-43924 |
| Algae_064-1_Unigene_BMK.24456 |  | stu-miR171b-3p |
| Algae_064-1_Unigene_BMK.24525 | NAD(P)H azoreductase OS=Xenophilus azovorans GN=azoB PE=1 SV=2 | sbi-miR169c |
| Algae_064-1_Unigene_BMK.24526 | Trithorax group protein osa OS=Drosophila melanogaster GN=osa PE=1 SV=1 | stu-miR171b-3p |
| Algae_064-1_Unigene_BMK.2454 |  | aca-miR-5p-43924 |
| Algae_064-1_Unigene_BMK.24658 | Voltage-dependent N-type calcium channel subunit alpha-1B OS=Rattus norvegicus GN=Cacna1b PE=1 SV=1 | aca-miR-5p-43924 |
| Algae_064-1_Unigene_BMK.24667 | UPF0301 protein SRU_0495 OS=Salinibacter ruber (strain DSM 13855 / M31) GN=SRU_0495 PE=3 SV=1 | aca-miR-5p-43924 |
| Algae_064-1_Unigene_BMK.24804 | F-box/WD repeat-containing protein 7 OS=Mus musculus GN=Fbxw7 PE=1 SV=1 | bdi-miR7732-3p_L-1_1ss11GC |
| Algae_064-1_Unigene_BMK.24821 | Eukaryotic translation initiation factor 6 OS=Toxoplasma gondii GN=EIF6 PE=3 SV=2 | tae-miR159a |
| Algae_064-1_Unigene_BMK.24972 | Sodium channel protein type 10 subunit alpha OS=Rattus norvegicus GN=Scn10a PE=1 SV=1 | bdi-miR7732-3p_L-1_1ss11GC |
| Algae_064-1_Unigene_BMK.2506 |  | sbi-miR169c |
| Algae_064-1_Unigene_BMK.25196 |  | bdi-miR7732-3p_L-1_1ss11GC |
| Algae_064-1_Unigene_BMK.25265 | Peptidyl-prolyl cis-trans isomerase OS=Acinetobacter sp. (strain ADP1) GN=rotA PE=3 SV=1 | osa-miR168a-5p |
| Algae_064-1_Unigene_BMK.25539 | Uncharacterized protein DDB_G0271670 OS=Dictyostelium discoideum GN=DDB_G0271670 PE=4 SV=1 | aca-miR-5p-43924 |
| Algae_064-1_Unigene_BMK.25542 | Glucose-6-phosphate/phosphate translocator 1, chloroplastic OS=Arabidopsis thaliana GN=GPT1 PE=2 SV=1 | sbi-miR169c |
| Algae_064-1_Unigene_BMK.2560 | Pumilio homolog 1 OS=Pongo abelii GN=PUM1 PE=2 SV=1 | rgl-miR5139_L+3 |
| Algae_064-1_Unigene_BMK.2574 | Side tail fiber protein homolog from lambdoid prophage Rac OS=Escherichia coli (strain K12) GN=stfR PE=3 SV=2 | aca-miR-3p-456915 |
| Algae_064-1_Unigene_BMK.25763 | TNF receptor-associated factor 6-B OS=Xenopus laevis GN=traf6-b PE=2 SV=1 | sbi-miR169c |
| Algae_064-1_Unigene_BMK.25832 | Glycoprotein gp2 OS=Equine herpesvirus 1 (strain Ab4p) GN=EUs4 PE=4 SV=1 | zma-miR529-5p |
| Algae_064-1_Unigene_BMK.2588 | Vegetative incompatibility protein HET-E-1 OS=Podospora anserina GN=HET-E1 PE=4 SV=1 | stu-miR171b-3p |
| Algae_064-1_Unigene_BMK.25931 | [NU+] prion formation protein 1 OS=Saccharomyces cerevisiae GN=NEW1 PE=1 SV=1 | sbi-miR169c |
| Algae_064-1_Unigene_BMK.2627 | Cyclic nucleotide-gated channel cone photoreceptor subunit alpha OS=Gallus gallus PE=2 SV=1 | osa-miR168a-5p |
| Algae_064-1_Unigene_BMK.263 | Serine/arginine repetitive matrix protein 2 OS=Mus musculus GN=Srrm2 PE=1 SV=2 | tae-miR159a |
| Algae_064-1_Unigene_BMK.26378 |  | zma-miR529-5p |
| Algae_064-1_Unigene_BMK.26772 |  | sbi-miR169c |
| Algae_064-1_Unigene_BMK.26777 | Serine/arginine repetitive matrix protein 2 OS=Mus musculus GN=Srrm2 PE=1 SV=2 | aca-miR-5p-43924 |
| Algae_064-1_Unigene_BMK.26778 |  | osa-miR2876-3p_R+1 |
| Algae_064-1_Unigene_BMK.26813 | Uncharacterized WD repeat-containing protein alr3466 OS=Nostoc sp. (strain PCC 7120 / UTEX 2576) GN=alr3466 PE=4 SV=1 | aca-miR-3p-456915 |
| Algae_064-1_Unigene_BMK.26876 |  | bdi-miR7732-3p_L-1_1ss11GC |
| Algae_064-1_Unigene_BMK.26955 | Uncharacterized protein DDB_G0271670 OS=Dictyostelium discoideum GN=DDB_G0271670 PE=4 SV=1 | sbi-miR169c |
| Algae_064-1_Unigene_BMK.2708 | DTW domain-containing protein yfiP OS=Escherichia coli (strain K12) GN=yfiP PE=3 SV=2 | aca-miR-3p-456915 |
| Algae_064-1_Unigene_BMK.27176 | Uncharacterized protein DDB_G0271670 OS=Dictyostelium discoideum GN=DDB_G0271670 PE=4 SV=1 | stu-miR171b-3p |
| Algae_064-1_Unigene_BMK.27749 | Argininosuccinate lyase 2 OS=Rhizobium loti GN=argH2 PE=3 SV=1 | aca-miR-3p-456915 |
| Algae_064-1_Unigene_BMK.27987 | UPF0317 protein KRH_21160 OS=Kocuria rhizophila (strain ATCC 9341 / DSM 348 / NBRC 103217 / DC2201) GN=KRH_21160 PE=3 SV=1 | tae-miR159a |
| Algae_064-1_Unigene_BMK.2809 | Protein disulfide-isomerase-like protein EhSep2 OS=Emiliania huxleyi GN=SEP2 PE=1 SV=2 | aca-miR-5p-43924 |
| Algae_064-1_Unigene_BMK.28112 | Basic proline-rich protein OS=Sus scrofa PE=1 SV=2 | aca-miR-5p-43924 |
| Algae_064-1_Unigene_BMK.2829 | Systemin receptor SR160 OS=Solanum peruvianum PE=1 SV=1 | sbi-miR169c |
| Algae_064-1_Unigene_BMK.28382 | Putative protein TPRXL OS=Homo sapiens GN=TPRXL PE=5 SV=2 | bdi-miR7732-3p_L-1_1ss11GC |
| Algae_064-1_Unigene_BMK.28545 | Uncharacterized serine/threonine-rich protein PB15E9.01c OS=Schizosaccharomyces pombe GN=SPAPB15E9.01c PE=2 SV=2 | osa-miR2876-3p_R+1 |
| Algae_064-1_Unigene_BMK.28564 | 6-phosphofructo-2-kinase/fructose-2,6-biphosphatase OS=Gallus gallus PE=2 SV=2 | stu-miR171b-3p |
| Algae_064-1_Unigene_BMK.2870 | Translation initiation factor IF-2 OS=Streptomyces griseus subsp. griseus (strain JCM 4626 / NBRC 13350) GN=infB PE=3 SV=1 | osa-miR168a-5p |
| Algae_064-1_Unigene_BMK.28753 | Pre-mRNA polyadenylation factor FIP1 OS=Debaryomyces hansenii GN=FIP1 PE=3 SV=2 | osa-miR168a-5p |
| Algae_064-1_Unigene_BMK.29020 | Basic proline-rich protein OS=Sus scrofa PE=1 SV=2 | zma-miR529-5p |
| Algae_064-1_Unigene_BMK.29414 | Translation initiation factor IF-2 OS=Rhodococcus opacus (strain B4) GN=infB PE=3 SV=1 | osa-miR168a-5p |
| Algae_064-1_Unigene_BMK.29445 | Uncharacterized protein BHLF1 OS=Epstein-Barr virus (strain B95-8) GN=BHLF1 PE=4 SV=1 | stu-miR171b-3p |
| Algae_064-1_Unigene_BMK.29685 | Serine/arginine repetitive matrix protein 2 OS=Homo sapiens GN=SRRM2 PE=1 SV=2 | bdi-miR7732-3p_L-1_1ss11GC |
| Algae_064-1_Unigene_BMK.29710 | Putative monooxygenase Rv1533 OS=Mycobacterium tuberculosis GN=Rv1533 PE=3 SV=1 | zma-miR529-5p |
| Algae_064-1_Unigene_BMK.29797 | DNA mismatch repair protein Mlh1 OS=Mus musculus GN=Mlh1 PE=2 SV=1 | zma-miR529-5p |
| Algae_064-1_Unigene_BMK.29827 | Formin-2 OS=Homo sapiens GN=FMN2 PE=1 SV=4 | tae-miR159a |
| Algae_064-1_Unigene_BMK.29883 | Fucoxanthin-chlorophyll a-c binding protein A, chloroplastic OS=Macrocystis pyrifera GN=FCPA PE=2 SV=1 | sbi-miR169c |
| Algae_064-1_Unigene_BMK.30087 | Acyl-coenzyme A oxidase OS=Kluyveromyces lactis GN=POX1 PE=3 SV=1 | zma-miR529-5p |
| Algae_064-1_Unigene_BMK.30149 | Serine/arginine repetitive matrix protein 2 OS=Mus musculus GN=Srrm2 PE=1 SV=2 | aca-miR-5p-43924 |
| Algae_064-1_Unigene_BMK.30427 | Putative diflavin flavoprotein A 3 OS=Nostoc sp. (strain PCC 7120 / UTEX 2576) GN=dfa3 PE=1 SV=1 | tae-miR159a |
| Algae_064-1_Unigene_BMK.30503 |  | osa-miR168a-5p |
| Algae_064-1_Unigene_BMK.30538 | Gramicidin S biosynthesis protein GrsT OS=Aneurinibacillus migulanus GN=grsT PE=3 SV=1 | rgl-miR5139_L+3 |
| Algae_064-1_Unigene_BMK.30557 |  | stu-miR171b-3p |
| Algae_064-1_Unigene_BMK.30972 |  | sbi-miR169c |
| Algae_064-1_Unigene_BMK.31185 | Charged multivesicular body protein 3 OS=Pongo abelii GN=VPS24 PE=2 SV=3 | sbi-miR169c |
| Algae_064-1_Unigene_BMK.31204 | Latency-related protein 1 OS=Human herpesvirus 1 (strain F) PE=4 SV=1 | sbi-miR169c |
| Algae_064-1_Unigene_BMK.3139 | Serine/arginine repetitive matrix protein 1 OS=Gallus gallus GN=SRRM1 PE=2 SV=1 | rgl-miR5139_L+3 |
| Algae_064-1_Unigene_BMK.31399 |  | aca-miR-5p-43924 |
| Algae_064-1_Unigene_BMK.31637 | NAD-dependent deacetylase 2 OS=Pseudomonas aeruginosa GN=cobB2 PE=3 SV=1 | stu-miR171b-3p |
| Algae_064-1_Unigene_BMK.31665 |  | osa-miR168a-5p |
| Algae_064-1_Unigene_BMK.3174 | Myc-associated zinc finger protein (Fragment) OS=Mesocricetus auratus GN=MAZ PE=2 SV=1 | sbi-miR169c |
| Algae_064-1_Unigene_BMK.31832 | 60S ribosomal protein L27 OS=Candida albicans GN=RPL27 PE=2 SV=1 | sbi-miR169c |
| Algae_064-1_Unigene_BMK.3206 | Replication factor A protein 2 OS=Schizosaccharomyces pombe GN=ssb2 PE=1 SV=1 | sbi-miR169c |
| Algae_064-1_Unigene_BMK.32121 | Mucin-19 OS=Mus musculus GN=Muc19 PE=2 SV=2 | aca-miR-5p-43924 |
| Algae_064-1_Unigene_BMK.32217 | Putative mediator of RNA polymerase II transcription subunit 26 OS=Dictyostelium discoideum GN=med26 PE=3 SV=2 | rgl-miR5139_L+3 |
| Algae_064-1_Unigene_BMK.32656 |  | zma-miR529-5p |
| Algae_064-1_Unigene_BMK.328 | Serine/arginine repetitive matrix protein 1 OS=Mus musculus GN=Srrm1 PE=1 SV=1 | aca-miR-5p-43924 |
| Algae_064-1_Unigene_BMK.32821 | Tetratricopeptide repeat protein 28 OS=Homo sapiens GN=TTC28 PE=1 SV=4 | sbi-miR169c |
| Algae_064-1_Unigene_BMK.3350 | Translation initiation factor IF-2 OS=Synechococcus sp. (strain CC9902) GN=infB PE=3 SV=1 | sbi-miR169c |
| Algae_064-1_Unigene_BMK.33695 |  | rgl-miR5139_L+3 |
| Algae_064-1_Unigene_BMK.3389 | Uncharacterized protein BHLF1 OS=Epstein-Barr virus (strain B95-8) GN=BHLF1 PE=4 SV=1 | rgl-miR5139_L+3 |
| Algae_064-1_Unigene_BMK.33913 | Putative protein TPRXL OS=Homo sapiens GN=TPRXL PE=5 SV=2 | sbi-miR169c |
| Algae_064-1_Unigene_BMK.3392 | DNA-directed RNA polymerase II subunit rpb1 OS=Schizosaccharomyces pombe GN=rpb1 PE=1 SV=1 | aca-miR-3p-456915 |
| Algae_064-1_Unigene_BMK.33931 |  | aca-miR-5p-43924 |
| Algae_064-1_Unigene_BMK.3399 | Putative zinc finger CCCH domain-containing protein 48 OS=Oryza sativa subsp. japonica GN=Os07g0139000 PE=4 SV=1 | aca-miR-5p-43924 |
| Algae_064-1_Unigene_BMK.34260 | Beta-glucosidase OS=Rhizobium radiobacter GN=cbg-1 PE=3 SV=1 | sbi-miR169c |
| Algae_064-1_Unigene_BMK.34269 |  | sbi-miR169c |
| Algae_064-1_Unigene_BMK.34506 | Anthocyanidin 3-O-glucosyltransferase 2 (Fragment) OS=Manihot esculenta GN=GT2 PE=2 SV=1 | sbi-miR169c |
| Algae_064-1_Unigene_BMK.34574 |  | osa-miR168a-5p |
| Algae_064-1_Unigene_BMK.34662 | Choline dehydrogenase OS=Agrobacterium tumefaciens (strain C58 / ATCC 33970) GN=betA PE=3 SV=2 | rgl-miR5139_L+3 |
| Algae_064-1_Unigene_BMK.34896 | Uncharacterized protein BHLF1 OS=Epstein-Barr virus (strain B95-8) GN=BHLF1 PE=4 SV=1 | sbi-miR169c |
| Algae_064-1_Unigene_BMK.35076 | Chaperone protein dnaJ OS=Thermotoga neapolitana (strain ATCC 49049 / DSM 4359 / NS-E) GN=dnaJ PE=3 SV=1 | osa-miR168a-5p |
| Algae_064-1_Unigene_BMK.3533 | Serine/arginine repetitive matrix protein 2 OS=Mus musculus GN=Srrm2 PE=1 SV=2 | zma-miR529-5p |
| Algae_064-1_Unigene_BMK.3545 | Stromal 70 kDa heat shock-related protein, chloroplastic OS=Pisum sativum GN=HSP70 PE=2 SV=1 | osa-miR168a-5p |
| Algae_064-1_Unigene_BMK.35604 | 3-hydroxyacyl-CoA dehydrogenase type-2 OS=Homo sapiens GN=HSD17B10 PE=1 SV=3 | stu-miR171b-3p |
| Algae_064-1_Unigene_BMK.3565 | Putative ankyrin-containing lipoprotein Lxx09580 OS=Leifsonia xyli subsp. xyli GN=Lxx09580 PE=4 SV=2 | aca-miR-3p-456915 |
| Algae_064-1_Unigene_BMK.35861 | Serine/arginine repetitive matrix protein 2 OS=Homo sapiens GN=SRRM2 PE=1 SV=2 | aca-miR-3p-456915 |
| Algae_064-1_Unigene_BMK.3618 |  | tae-miR159a |
| Algae_064-1_Unigene_BMK.36248 | Protein Mpv17 OS=Bos taurus GN=MPV17 PE=2 SV=1 | osa-miR168a-5p |
| Algae_064-1_Unigene_BMK.36384 | Canalicular multispecific organic anion transporter 2 OS=Rattus norvegicus GN=Abcc3 PE=2 SV=1 | aca-miR-5p-43924 |
| Algae_064-1_Unigene_BMK.36392 | Uroporphyrinogen decarboxylase OS=Salinibacter ruber (strain DSM 13855 / M31) GN=hemE PE=3 SV=1 | aca-miR-3p-456915 |
| Algae_064-1_Unigene_BMK.36582 |  | aca-miR-5p-43924 |
| Algae_064-1_Unigene_BMK.36794 |  | osa-miR168a-5p |
| Algae_064-1_Unigene_BMK.36802 |  | osa-miR2876-3p_R+1 |
| Algae_064-1_Unigene_BMK.36815 | Mucin-5AC (Fragments) OS=Homo sapiens GN=MUC5AC PE=1 SV=3 | aca-miR-3p-456915 |
| Algae_064-1_Unigene_BMK.369 | Mannose-6-phosphate isomerase OS=Macaca fascicularis GN=MPI PE=2 SV=3 | sbi-miR169c |
| Algae_064-1_Unigene_BMK.36901 | UPF0016 protein 4 OS=Arabidopsis thaliana GN=At1g25520 PE=2 SV=1 | osa-miR2876-3p_R+1 |
| Algae_064-1_Unigene_BMK.37105 | Translation initiation factor IF-2 OS=Frankia sp. (strain CcI3) GN=infB PE=3 SV=1 | rgl-miR5139_L+3 |
| Algae_064-1_Unigene_BMK.37134 |  | stu-miR171b-3p |
| Algae_064-1_Unigene_BMK.37290 | ABC transporter G family member 5 OS=Dictyostelium discoideum GN=abcG5 PE=3 SV=1 | stu-miR171b-3p |
| Algae_064-1_Unigene_BMK.3737 |  | rgl-miR5139_L+3 |
| Algae_064-1_Unigene_BMK.3752 | Nitronate monooxygenase OS=Pseudomonas aeruginosa GN=PA1024 PE=1 SV=1 | stu-miR171b-3p |
| Algae_064-1_Unigene_BMK.37751 | Chromosome-associated kinesin KIF4B OS=Homo sapiens GN=KIF4B PE=1 SV=2 | aca-miR-3p-456915 |
| Algae_064-1_Unigene_BMK.3790 | Peptidyl-prolyl cis-trans isomerase-like 1 OS=Aspergillus oryzae (strain ATCC 42149 / RIB 40) GN=cyp1 PE=3 SV=1 | osa-miR168a-5p |
| Algae_064-1_Unigene_BMK.37921 | Transmembrane and TPR repeat-containing protein 4 OS=Mus musculus GN=Tmtc4 PE=2 SV=1 | aca-miR-5p-43924 |
| Algae_064-1_Unigene_BMK.38206 | Splicing regulatory glutamine/lysine-rich protein 1 OS=Rattus norvegicus GN=Srek1 PE=1 SV=1 | sbi-miR169c |
| Algae_064-1_Unigene_BMK.38299 |  | bdi-miR7732-3p_L-1_1ss11GC |
| Algae_064-1_Unigene_BMK.38418 | Translation initiation factor IF-2 OS=Kineococcus radiotolerans (strain ATCC BAA-149 / DSM 14245 / SRS30216) GN=infB PE=3 SV=1 | aca-miR-3p-456915 |
| Algae_064-1_Unigene_BMK.3860 | ATP synthase subunit b, chloroplastic OS=Ochrosphaera neapolitana GN=atpF PE=3 SV=1 | tae-miR159a |
| Algae_064-1_Unigene_BMK.38941 | Troponin C, skeletal muscle OS=Mus musculus GN=Tnnc2 PE=1 SV=2 | aca-miR-5p-43924 |
| Algae_064-1_Unigene_BMK.39077 |  | aca-miR-5p-43924 |
| Algae_064-1_Unigene_BMK.39105 | Zinc finger protein 828 OS=Homo sapiens GN=ZNF828 PE=1 SV=2 | rgl-miR5139_L+3 |
| Algae_064-1_Unigene_BMK.3917 |  | aca-miR-3p-456915 |
| Algae_064-1_Unigene_BMK.39303 | Serine/threonine/tyrosine-interacting protein B OS=Xenopus laevis GN=styx-b PE=2 SV=1 | osa-miR168a-5p |
| Algae_064-1_Unigene_BMK.39445 | Eukaryotic translation initiation factor 3 subunit A OS=Neosartorya fischeri (strain ATCC 1020 / DSM 3700 / FGSC A1164 / NRRL 181) GN=tif32 PE=3 SV=1 | bdi-miR7732-3p_L-1_1ss11GC |
| Algae_064-1_Unigene_BMK.39706 |  | rgl-miR5139_L+3 |
| Algae_064-1_Unigene_BMK.40 |  | aca-miR-5p-43924 |
| Algae_064-1_Unigene_BMK.4000 | Serine carboxypeptidase-like 48 OS=Arabidopsis thaliana GN=SCPL48 PE=2 SV=2 | aca-miR-5p-43924 |
| Algae_064-1_Unigene_BMK.40280 | Serine/arginine repetitive matrix protein 2 OS=Homo sapiens GN=SRRM2 PE=1 SV=2 | stu-miR171b-3p |
| Algae_064-1_Unigene_BMK.40393 | Diacylglycerol O-acyltransferase 2B OS=Umbelopsis ramanniana GN=DGAT2B PE=1 SV=1 | sbi-miR169c |
| Algae_064-1_Unigene_BMK.40481 | Protein SpAN OS=Strongylocentrotus purpuratus GN=SPAN PE=2 SV=1 | aca-miR-5p-43924 |
| Algae_064-1_Unigene_BMK.40775 | 29 kDa ribonucleoprotein, chloroplastic OS=Arabidopsis thaliana GN=RBP29 PE=1 SV=2 | tae-miR159a |
| Algae_064-1_Unigene_BMK.41144 | Peroxisomal acyl-coenzyme A oxidase 1 OS=Bos taurus GN=ACOX1 PE=2 SV=1 | aca-miR-5p-43924 |
| Algae_064-1_Unigene_BMK.41887 |  | aca-miR-5p-43924 |
| Algae_064-1_Unigene_BMK.4206 | Glyceraldehyde-3-phosphate dehydrogenase, chloroplastic OS=Guillardia theta GN=GAPC1 PE=2 SV=1 | stu-miR171b-3p |
| Algae_064-1_Unigene_BMK.42067 |  | zma-miR529-5p |
| Algae_064-1_Unigene_BMK.42109 | Glycoprotein gp2 OS=Equine herpesvirus 1 (strain V592) GN=71 PE=3 SV=1 | tae-miR159a |
| Algae_064-1_Unigene_BMK.42131 | Uncharacterized protein At4g17910 OS=Arabidopsis thaliana GN=At4g17910 PE=2 SV=1 | stu-miR171b-3p |
| Algae_064-1_Unigene_BMK.42135 | Triose phosphate/phosphate translocator TPT, chloroplastic OS=Oryza sativa subsp. japonica GN=TPT PE=2 SV=1 | sbi-miR169c |
| Algae_064-1_Unigene_BMK.42219 | SET domain-containing protein 5 OS=Schizosaccharomyces pombe GN=set5 PE=1 SV=1 | rgl-miR5139_L+3 |
| Algae_064-1_Unigene_BMK.42227 | Serine/arginine repetitive matrix protein 2 OS=Homo sapiens GN=SRRM2 PE=1 SV=2 | aca-miR-5p-43924 |
| Algae_064-1_Unigene_BMK.4235 | Multidrug resistance protein 3 OS=Bacillus subtilis GN=bmr3 PE=1 SV=1 | aca-miR-5p-43924 |
| Algae_064-1_Unigene_BMK.42435 |  | sbi-miR169c |
| Algae_064-1_Unigene_BMK.4268 | Uncharacterized protein C10orf95 OS=Homo sapiens GN=C10orf95 PE=2 SV=1 | zma-miR529-5p |
| Algae_064-1_Unigene_BMK.42706 | Gibberellin 2-beta-dioxygenase 2 OS=Arabidopsis thaliana GN=GA2OX2 PE=2 SV=1 | stu-miR171b-3p |
| Algae_064-1_Unigene_BMK.4272 | Translation initiation factor IF-2 OS=Synechococcus sp. (strain CC9311) GN=infB PE=3 SV=1 | osa-miR168a-5p |
| Algae_064-1_Unigene_BMK.43010 | Probable leucine-rich repeat receptor-like protein kinase At1g35710 OS=Arabidopsis thaliana GN=At1g35710 PE=2 SV=1 | aca-miR-5p-43924 |
| Algae_064-1_Unigene_BMK.43149 | Dual specificity protein phosphatase OS=Chlamydomonas moewusii GN=VH-PTP13 PE=1 SV=1 | rgl-miR5139_L+3 |
| Algae_064-1_Unigene_BMK.43451 | Protein phosphatase PTC7 homolog OS=Danio rerio GN=pptc7 PE=2 SV=1 | aca-miR-5p-43924 |
| Algae_064-1_Unigene_BMK.43527 | Enoyl-CoA hydratase domain-containing protein 3, mitochondrial OS=Danio rerio GN=echdc3 PE=2 SV=2 | tae-miR159a |
| Algae_064-1_Unigene_BMK.43571 | Calphotin OS=Drosophila melanogaster GN=Cpn PE=1 SV=2 | osa-miR168a-5p |
| Algae_064-1_Unigene_BMK.44038 |  | zma-miR529-5p |
| Algae_064-1_Unigene_BMK.44204 | Mucin-19 OS=Mus musculus GN=Muc19 PE=2 SV=2 | sbi-miR169c |
| Algae_064-1_Unigene_BMK.44590 | Renalase OS=Rattus norvegicus GN=Rnls PE=2 SV=1 | sbi-miR169c |
| Algae_064-1_Unigene_BMK.44865 | Argininosuccinate lyase 2 OS=Rhizobium loti GN=argH2 PE=3 SV=1 | zma-miR529-5p |
| Algae_064-1_Unigene_BMK.44895 |  | stu-miR171b-3p |
| Algae_064-1_Unigene_BMK.45276 | Translation initiation factor IF-2 OS=Rhodococcus erythropolis (strain PR4 / NBRC 100887) GN=infB PE=3 SV=1 | sbi-miR169c |
| Algae_064-1_Unigene_BMK.4528 | Probable dipeptidase B OS=Streptococcus pyogenes serotype M1 GN=pepDB PE=3 SV=1 | aca-miR-5p-43924 |
| Algae_064-1_Unigene_BMK.4542 |  | tae-miR159a |
| Algae_064-1_Unigene_BMK.45434 | Vegetative cell wall protein gp1 OS=Chlamydomonas reinhardtii GN=GP1 PE=2 SV=1 | sbi-miR169c |
| Algae_064-1_Unigene_BMK.455 | Putative protein TPRXL OS=Homo sapiens GN=TPRXL PE=5 SV=2 | aca-miR-3p-456915 |
| Algae_064-1_Unigene_BMK.45607 |  | zma-miR529-5p |
| Algae_064-1_Unigene_BMK.45651 | Putative protein TPRXL OS=Homo sapiens GN=TPRXL PE=5 SV=2 | stu-miR171b-3p |
| Algae_064-1_Unigene_BMK.45688 | Extended synaptotagmin-1 OS=Pongo abelii GN=ESYT1 PE=2 SV=2 | aca-miR-5p-43924 |
| Algae_064-1_Unigene_BMK.45952 | Pepsin B OS=Canis familiaris GN=PGB PE=1 SV=1 | sbi-miR169c |
| Algae_064-1_Unigene_BMK.46218 | Uncharacterized protein DDB_G0271670 OS=Dictyostelium discoideum GN=DDB_G0271670 PE=4 SV=1 | stu-miR171b-3p |
| Algae_064-1_Unigene_BMK.46248 | Probable 1,4-beta-D-glucan cellobiohydrolase B OS=Aspergillus terreus (strain NIH 2624 / FGSC A1156) GN=cbhB PE=3 SV=1 | osa-miR168a-5p |
| Algae_064-1_Unigene_BMK.46421 | Ankyrin repeat, PH and SEC7 domain containing protein secG OS=Dictyostelium discoideum GN=secG PE=2 SV=1 | sbi-miR169c |
| Algae_064-1_Unigene_BMK.4647 | Mitogen-activated protein kinase kinase 1 OS=Arabidopsis thaliana GN=MKK1 PE=1 SV=2 | osa-miR168a-5p |
| Algae_064-1_Unigene_BMK.47201 | Serine/arginine repetitive matrix protein 2 OS=Homo sapiens GN=SRRM2 PE=1 SV=2 | aca-miR-5p-43924 |
| Algae_064-1_Unigene_BMK.47247 | Potassium/sodium hyperpolarization-activated cyclic nucleotide-gated channel 3 OS=Homo sapiens GN=HCN3 PE=2 SV=2 | tae-miR159a |
| Algae_064-1_Unigene_BMK.4729 | Kinesin-like protein KIF2A OS=Xenopus laevis GN=kif2a PE=1 SV=2 | zma-miR529-5p |
| Algae_064-1_Unigene_BMK.474 | Degreening-related gene dee76 protein OS=Chlorella protothecoides GN=DEE76 PE=2 SV=1 | aca-miR-5p-43924 |
| Algae_064-1_Unigene_BMK.47608 | Serine/arginine repetitive matrix protein 1 OS=Gallus gallus GN=SRRM1 PE=2 SV=1 | aca-miR-3p-456915 |
| Algae_064-1_Unigene_BMK.47653 |  | zma-miR529-5p |
| Algae_064-1_Unigene_BMK.47689 | Mucin-19 OS=Mus musculus GN=Muc19 PE=2 SV=2 | osa-miR168a-5p |
| Algae_064-1_Unigene_BMK.48021 | Ubiquitin-conjugating enzyme E2 27 OS=Arabidopsis thaliana GN=UBC27 PE=2 SV=1 | sbi-miR169c |
| Algae_064-1_Unigene_BMK.48709 | UDP-glucuronosyltransferase 2B17 OS=Mus musculus GN=Ugt2b17 PE=2 SV=1 | aca-miR-5p-43924 |
| Algae_064-1_Unigene_BMK.48953 | Golgi to ER traffic protein 4 homolog OS=Taeniopygia guttata GN=GET4 PE=2 SV=1 | zma-miR529-5p |
| Algae_064-1_Unigene_BMK.4943 | Putative protein TPRXL OS=Homo sapiens GN=TPRXL PE=5 SV=2 | aca-miR-5p-43924 |
| Algae_064-1_Unigene_BMK.4961 | Phosphatidylinositol-4-phosphate 5-kinase 8 OS=Arabidopsis thaliana GN=PIP5K8 PE=2 SV=1 | zma-miR529-5p |
| Algae_064-1_Unigene_BMK.49865 |  | osa-miR168a-5p |
| Algae_064-1_Unigene_BMK.5003 | Uncharacterized protein DDB_G0271670 OS=Dictyostelium discoideum GN=DDB_G0271670 PE=4 SV=1 | stu-miR171b-3p |
| Algae_064-1_Unigene_BMK.50119 | Protein ETHE1, mitochondrial OS=Homo sapiens GN=ETHE1 PE=1 SV=2 | aca-miR-3p-456915 |
| Algae_064-1_Unigene_BMK.5017 | Uncharacterized protein LOC284861 OS=Homo sapiens PE=2 SV=1 | aca-miR-3p-456915 |
| Algae_064-1_Unigene_BMK.50656 | Mucin-5AC (Fragments) OS=Homo sapiens GN=MUC5AC PE=1 SV=3 | osa-miR168a-5p |
| Algae_064-1_Unigene_BMK.50714 |  | aca-miR-5p-43924 |
| Algae_064-1_Unigene_BMK.50976 | ATP-dependent zinc metalloprotease FtsH OS=Guillardia theta GN=ftsH PE=3 SV=1 | aca-miR-5p-43924 |
| Algae_064-1_Unigene_BMK.51046 |  | bdi-miR7732-3p_L-1_1ss11GC |
| Algae_064-1_Unigene_BMK.5114 | Translation initiation factor IF-2 OS=Thermobifida fusca (strain YX) GN=infB PE=3 SV=1 | stu-miR171b-3p |
| Algae_064-1_Unigene_BMK.51361 | Violaxanthin de-epoxidase, chloroplastic OS=Lactuca sativa GN=VDE1 PE=1 SV=1 | bdi-miR7732-3p_L-1_1ss11GC |
| Algae_064-1_Unigene_BMK.51383 | Serine/arginine repetitive matrix protein 2 OS=Homo sapiens GN=SRRM2 PE=1 SV=2 | zma-miR529-5p |
| Algae_064-1_Unigene_BMK.51421 | Nucleolar protein 58 OS=Lodderomyces elongisporus GN=NOP58 PE=3 SV=1 | osa-miR168a-5p |
| Algae_064-1_Unigene_BMK.51491 | Calcium-dependent protein kinase 2 OS=Plasmodium falciparum (isolate K1 / Thailand) GN=CPK2 PE=1 SV=3 | aca-miR-3p-456915 |
| Algae_064-1_Unigene_BMK.51532 | Influenza virus NS1A-binding protein homolog OS=Mus musculus GN=Ivns1abp PE=1 SV=2 | sbi-miR169c |
| Algae_064-1_Unigene_BMK.51580 | Putative protein TPRXL OS=Homo sapiens GN=TPRXL PE=5 SV=2 | sbi-miR169c |
| Algae_064-1_Unigene_BMK.51710 | Mucin-5AC (Fragments) OS=Homo sapiens GN=MUC5AC PE=1 SV=3 | sbi-miR169c |
| Algae_064-1_Unigene_BMK.52146 | Serine/arginine repetitive matrix protein 1 OS=Gallus gallus GN=SRRM1 PE=2 SV=1 | aca-miR-3p-456915 |
| Algae_064-1_Unigene_BMK.52218 | Uncharacterized symporter yidK OS=Escherichia coli (strain K12) GN=yidK PE=1 SV=1 | sbi-miR169c |
| Algae_064-1_Unigene_BMK.525 | Heparan-alpha-glucosaminide N-acetyltransferase OS=Mus musculus GN=Hgsnat PE=1 SV=1 | sbi-miR169c |
| Algae_064-1_Unigene_BMK.52784 |  | aca-miR-3p-456915 |
| Algae_064-1_Unigene_BMK.5316 |  | osa-miR2876-3p_R+1 |
| Algae_064-1_Unigene_BMK.53610 | Protein NLRC3 OS=Mus musculus GN=Nlrc3 PE=2 SV=2 | aca-miR-5p-43924 |
| Algae_064-1_Unigene_BMK.53855 | Uncharacterized protein y4nG OS=Rhizobium sp. (strain NGR234) GN=NGR_a02350 PE=4 SV=1 | stu-miR171b-3p |
| Algae_064-1_Unigene_BMK.54171 |  | rgl-miR5139_L+3 |
| Algae_064-1_Unigene_BMK.5462 |  | zma-miR529-5p |
| Algae_064-1_Unigene_BMK.54658 | Putative protein TPRXL OS=Homo sapiens GN=TPRXL PE=5 SV=2 | stu-miR171b-3p |
| Algae_064-1_Unigene_BMK.54778 | Serine/threonine-protein phosphatase PP1-gamma catalytic subunit OS=Xenopus tropicalis GN=ppp1cc PE=2 SV=1 | stu-miR171b-3p |
| Algae_064-1_Unigene_BMK.54874 |  | aca-miR-5p-43924 |
| Algae_064-1_Unigene_BMK.55612 | Putative branched-chain-amino-acid aminotransferase OS=Methanobacterium thermoautotrophicum GN=ilvE PE=3 SV=2 | aca-miR-5p-43924 |
| Algae_064-1_Unigene_BMK.55850 | Uncharacterized serine/threonine-rich protein PB15E9.01c OS=Schizosaccharomyces pombe GN=SPAPB15E9.01c PE=2 SV=2 | aca-miR-5p-43924 |
| Algae_064-1_Unigene_BMK.55854 |  | bdi-miR7732-3p_L-1_1ss11GC |
| Algae_064-1_Unigene_BMK.56014 | Immediate-early protein IE180 OS=Suid herpesvirus 1 (strain Indiana-Funkhauser / Becker) GN=IE PE=3 SV=2 | rgl-miR5139_L+3 |
| Algae_064-1_Unigene_BMK.5604 | Chloride channel protein CLC-d OS=Arabidopsis thaliana GN=CLC-D PE=1 SV=2 | tae-miR159a |
| Algae_064-1_Unigene_BMK.56058 | Kelch-like protein 17 OS=Homo sapiens GN=KLHL17 PE=2 SV=1 | osa-miR2876-3p_R+1 |
| Algae_064-1_Unigene_BMK.56517 | Endoglycoceramidase OS=Hydra vulgaris PE=1 SV=1 | aca-miR-3p-456915 |
| Algae_064-1_Unigene_BMK.56914 | Probable galacturonosyltransferase-like 3 OS=Arabidopsis thaliana GN=GATL3 PE=2 SV=1 | sbi-miR169c |
| Algae_064-1_Unigene_BMK.5703 | Hypersensitive-induced response protein 3 OS=Arabidopsis thaliana GN=HIR3 PE=1 SV=1 | aca-miR-5p-43924 |
| Algae_064-1_Unigene_BMK.57122 | Protamine OS=Osilinus turbinatus PE=1 SV=1 | aca-miR-5p-43924 |
| Algae_064-1_Unigene_BMK.57255 | Ribonucleoside-diphosphate reductase large chain OS=Schizosaccharomyces pombe GN=cdc22 PE=1 SV=2 | zma-miR529-5p |
| Algae_064-1_Unigene_BMK.57641 |  | bdi-miR7732-3p_L-1_1ss11GC |
| Algae_064-1_Unigene_BMK.5799 | Ribosomal RNA large subunit methyltransferase N OS=Rhodopirellula baltica GN=rlmN PE=3 SV=1 | aca-miR-5p-43924 |
| Algae_064-1_Unigene_BMK.58388 | Probable serine/threonine-protein kinase drkC OS=Dictyostelium discoideum GN=drkC PE=3 SV=1 | osa-miR168a-5p |
| Algae_064-1_Unigene_BMK.58399 | Protein terminal ear1 OS=Zea mays GN=TE1 PE=2 SV=1 | sbi-miR169c |
| Algae_064-1_Unigene_BMK.58654 | Potential protein lysine methyltransferase SET5 OS=Vanderwaltozyma polyspora (strain ATCC 22028 / DSM 70294) GN=SET5 PE=3 SV=1 | sbi-miR169c |
| Algae_064-1_Unigene_BMK.58777 | Uncharacterized aminotransferase YhxA OS=Bacillus subtilis GN=yhxA PE=3 SV=4 | stu-miR171b-3p |
| Algae_064-1_Unigene_BMK.58831 | Immediate-early protein IE180 OS=Suid herpesvirus 1 (strain Indiana-Funkhauser / Becker) GN=IE PE=3 SV=2 | aca-miR-5p-43924 |
| Algae_064-1_Unigene_BMK.5887 | Caffeoyl-CoA O-methyltransferase 5 OS=Nicotiana tabacum GN=CCOAOMT5 PE=2 SV=1 | sbi-miR169c |
| Algae_064-1_Unigene_BMK.58895 | Uncharacterized protein BHLF1 OS=Epstein-Barr virus (strain B95-8) GN=BHLF1 PE=4 SV=1 | aca-miR-5p-43924 |
| Algae_064-1_Unigene_BMK.5924 | Aliphatic amidase OS=Pseudomonas fluorescens (strain SBW25) GN=amiE PE=3 SV=1 | bdi-miR7732-3p_L-1_1ss11GC |
| Algae_064-1_Unigene_BMK.59320 | Phosphoribulokinase, chloroplastic OS=Chlamydomonas reinhardtii GN=PRKA PE=1 SV=1 | zma-miR529-5p |
| Algae_064-1_Unigene_BMK.5962 | Proline-rich protein 2 OS=Mus musculus GN=Prp2 PE=2 SV=2 | sbi-miR169c |
| Algae_064-1_Unigene_BMK.5993 |  | aca-miR-5p-43924 |
| Algae_064-1_Unigene_BMK.60157 |  | aca-miR-3p-456915 |
| Algae_064-1_Unigene_BMK.6017 | Metacaspase-1 OS=Ashbya gossypii (strain ATCC 10895 / CBS 109.51 / FGSC 9923 / NRRL Y-1056) GN=MCA1 PE=3 SV=1 | aca-miR-3p-456915 |
| Algae_064-1_Unigene_BMK.60401 |  | rgl-miR5139_L+3 |
| Algae_064-1_Unigene_BMK.6045 | Receptor-interacting serine/threonine-protein kinase 3 OS=Rattus norvegicus GN=Ripk3 PE=2 SV=2 | stu-miR171b-3p |
| Algae_064-1_Unigene_BMK.6067 | Proline-rich protein 2 OS=Mus musculus GN=Prp2 PE=2 SV=2 | stu-miR171b-3p |
| Algae_064-1_Unigene_BMK.6089 |  | zma-miR529-5p |
| Algae_064-1_Unigene_BMK.60955 | Cyclin-U4-1 OS=Arabidopsis thaliana GN=CYCU4-1 PE=1 SV=1 | tae-miR159a |
| Algae_064-1_Unigene_BMK.6123 | Serine/arginine repetitive matrix protein 2 OS=Homo sapiens GN=SRRM2 PE=1 SV=2 | bdi-miR7732-3p_L-1_1ss11GC |
| Algae_064-1_Unigene_BMK.61409 | Tetracycline resistance protein, class C OS=Escherichia coli GN=tetA PE=1 SV=1 | aca-miR-3p-456915 |
| Algae_064-1_Unigene_BMK.61549 | Radial spoke head 10 homolog B OS=Bos taurus GN=RSPH10B PE=2 SV=2 | sbi-miR169c |
| Algae_064-1_Unigene_BMK.6199 | Protein terminal ear1 homolog OS=Oryza sativa subsp. indica GN=PLA2 PE=2 SV=1 | zma-miR529-5p |
| Algae_064-1_Unigene_BMK.62017 | Endoglucanase EG-1 OS=Trichoderma longibrachiatum GN=egl1 PE=3 SV=1 | aca-miR-3p-456915 |
| Algae_064-1_Unigene_BMK.62505 | Synapsin-1 OS=Bos taurus GN=SYN1 PE=1 SV=2 | osa-miR168a-5p |
| Algae_064-1_Unigene_BMK.62658 | Phthiocerol synthesis polyketide synthase type I PpsA OS=Mycobacterium tuberculosis GN=ppsA PE=3 SV=2 | sbi-miR169c |
| Algae_064-1_Unigene_BMK.62670 | Serine/arginine repetitive matrix protein 2 OS=Homo sapiens GN=SRRM2 PE=1 SV=2 | osa-miR168a-5p |
| Algae_064-1_Unigene_BMK.631 | Sterol 3-beta-glucosyltransferase OS=Aspergillus oryzae (strain ATCC 42149 / RIB 40) GN=atg26 PE=3 SV=2 | aca-miR-5p-43924 |
| Algae_064-1_Unigene_BMK.63360 | Uncharacterized protein Rv1367c/MT1414 OS=Mycobacterium tuberculosis GN=Rv1367c PE=4 SV=2 | aca-miR-5p-43924 |
| Algae_064-1_Unigene_BMK.63446 | Proline-rich protein 2 OS=Mus musculus GN=Prp2 PE=2 SV=2 | zma-miR529-5p |
| Algae_064-1_Unigene_BMK.63866 |  | sbi-miR169c |
| Algae_064-1_Unigene_BMK.63981 | Pyrimidine-specific ribonucleoside hydrolase rihA OS=Salmonella typhimurium GN=rihA PE=3 SV=1 | zma-miR529-5p |
| Algae_064-1_Unigene_BMK.64131 | Spore coat polysaccharide biosynthesis protein spsC OS=Bacillus subtilis GN=spsC PE=3 SV=1 | osa-miR168a-5p |
| Algae_064-1_Unigene_BMK.64784 |  | osa-miR168a-5p |
| Algae_064-1_Unigene_BMK.6501 |  | rgl-miR5139_L+3 |
| Algae_064-1_Unigene_BMK.6505 | Type-3 glutamine synthetase OS=Dictyostelium discoideum GN=glnA3 PE=1 SV=1 | aca-miR-3p-456915 |
| Algae_064-1_Unigene_BMK.65343 | Corepressor interacting with RBPJ 1 OS=Mus musculus GN=Cir1 PE=1 SV=2 | stu-miR171b-3p |
| Algae_064-1_Unigene_BMK.65527 |  | aca-miR-5p-43924 |
| Algae_064-1_Unigene_BMK.65601 | Putative protein TPRXL OS=Homo sapiens GN=TPRXL PE=5 SV=2 | aca-miR-5p-43924 |
| Algae_064-1_Unigene_BMK.66031 | Beta,beta-carotene 9',10'-oxygenase OS=Pongo abelii GN=BCO2 PE=2 SV=1 | stu-miR171b-3p |
| Algae_064-1_Unigene_BMK.66403 | MOSC domain-containing protein 2, mitochondrial OS=Rattus norvegicus GN=Mosc2 PE=2 SV=1 | aca-miR-5p-43924 |
| Algae_064-1_Unigene_BMK.6679 | Cyclin-dependent kinase E-1 OS=Arabidopsis thaliana GN=CDKE-1 PE=1 SV=2 | aca-miR-3p-456915 |
| Algae_064-1_Unigene_BMK.66870 | Transmembrane protease serine 13 OS=Homo sapiens GN=TMPRSS13 PE=2 SV=3 | sbi-miR169c |
| Algae_064-1_Unigene_BMK.67555 | Uncharacterized ATP-dependent helicase C144.05 OS=Schizosaccharomyces pombe GN=SPAC144.05 PE=2 SV=1 | bdi-miR7732-3p_L-1_1ss11GC |
| Algae_064-1_Unigene_BMK.67666 | Phenylacetone monooxygenase OS=Thermobifida fusca (strain YX) GN=pamO PE=1 SV=1 | rgl-miR5139_L+3 |
| Algae_064-1_Unigene_BMK.67995 | G-type lectin S-receptor-like serine/threonine-protein kinase At1g11410 OS=Arabidopsis thaliana GN=At1g11410 PE=3 SV=3 | stu-miR171b-3p |
| Algae_064-1_Unigene_BMK.68025 | Translation initiation factor IF-2 OS=Arthrobacter chlorophenolicus (strain A6 / ATCC 700700 / DSM 12829 / JCM 12360) GN=infB PE=3 SV=1 | aca-miR-5p-43924 |
| Algae_064-1_Unigene_BMK.68508 | Putative protein TPRXL OS=Homo sapiens GN=TPRXL PE=5 SV=2 | aca-miR-3p-456915 |
| Algae_064-1_Unigene_BMK.68581 |  | sbi-miR169c |
| Algae_064-1_Unigene_BMK.6868 | Putative uncharacterized protein ENSP00000383309 OS=Homo sapiens PE=5 SV=3 | sbi-miR169c |
| Algae_064-1_Unigene_BMK.68681 |  | bdi-miR7732-3p_L-1_1ss11GC |
| Algae_064-1_Unigene_BMK.68865 | Golgi apparatus protein 1 OS=Gallus gallus GN=GLG1 PE=1 SV=1 | stu-miR171b-3p |
| Algae_064-1_Unigene_BMK.68979 | Putative protein TPRXL OS=Homo sapiens GN=TPRXL PE=5 SV=2 | zma-miR529-5p |
| Algae_064-1_Unigene_BMK.6903 |  | zma-miR529-5p |
| Algae_064-1_Unigene_BMK.692 | Sodium channel protein type 8 subunit alpha OS=Rattus norvegicus GN=Scn8a PE=2 SV=1 | aca-miR-3p-456915 |
| Algae_064-1_Unigene_BMK.6949 | Vegetative incompatibility protein HET-E-1 OS=Podospora anserina GN=HET-E1 PE=4 SV=1 | aca-miR-5p-43924 |
| Algae_064-1_Unigene_BMK.69573 |  | bdi-miR7732-3p_L-1_1ss11GC |
| Algae_064-1_Unigene_BMK.69700 | Putative protein TPRXL OS=Homo sapiens GN=TPRXL PE=5 SV=2 | sbi-miR169c |
| Algae_064-1_Unigene_BMK.70088 | Nuclear pore complex protein Nup98-Nup96 OS=Rattus norvegicus GN=Nup98 PE=1 SV=2 | aca-miR-5p-43924 |
| Algae_064-1_Unigene_BMK.7017 | Major facilitator superfamily domain-containing protein 7 OS=Danio rerio GN=mfsd7 PE=2 SV=1 | bdi-miR7732-3p_L-1_1ss11GC |
| Algae_064-1_Unigene_BMK.70321 | Serine/threonine-protein phosphatase 1 regulatory subunit 10 OS=Pan troglodytes GN=PPP1R10 PE=3 SV=1 | osa-miR168a-5p |
| Algae_064-1_Unigene_BMK.7053 | Malate dehydrogenase 1, mitochondrial OS=Arabidopsis thaliana GN=At1g53240 PE=1 SV=1 | sbi-miR169c |
| Algae_064-1_Unigene_BMK.7065 | Probable enoyl-CoA hydratase echA6 OS=Mycobacterium tuberculosis GN=echA6 PE=1 SV=1 | aca-miR-5p-43924 |
| Algae_064-1_Unigene_BMK.70839 | Major facilitator superfamily domain-containing protein 5 OS=Xenopus tropicalis GN=mfsd5 PE=2 SV=1 | osa-miR168a-5p |
| Algae_064-1_Unigene_BMK.71248 | Formin-like protein 6 OS=Oryza sativa subsp. japonica GN=FH6 PE=2 SV=2 | stu-miR171b-3p |
| Algae_064-1_Unigene_BMK.72072 | Translation initiation factor IF-2 OS=Corynebacterium glutamicum GN=infB PE=3 SV=1 | aca-miR-5p-43924 |
| Algae_064-1_Unigene_BMK.72158 |  | bdi-miR7732-3p_L-1_1ss11GC |
| Algae_064-1_Unigene_BMK.72199 | Immediate-early protein OS=Saimiriine herpesvirus 2 (strain 11) GN=73 PE=3 SV=1 | osa-miR168a-5p |
| Algae_064-1_Unigene_BMK.72235 |  | bdi-miR7732-3p_L-1_1ss11GC |
| Algae_064-1_Unigene_BMK.72285 | Putative tyrosinase-like protein tyr-3 OS=Caenorhabditis elegans GN=tyr-3 PE=2 SV=5 | sbi-miR169c |
| Algae_064-1_Unigene_BMK.7264 | Transmembrane protein C2orf18 homolog OS=Pongo abelii PE=2 SV=1 | sbi-miR169c |
| Algae_064-1_Unigene_BMK.73124 |  | zma-miR529-5p |
| Algae_064-1_Unigene_BMK.73626 | Uncharacterized protein DDB_G0271670 OS=Dictyostelium discoideum GN=DDB_G0271670 PE=4 SV=1 | aca-miR-3p-456915 |
| Algae_064-1_Unigene_BMK.73810 | Carboxylesterase OS=Thermobifida fusca (strain YX) GN=Tfu_2427 PE=3 SV=1 | stu-miR171b-3p |
| Algae_064-1_Unigene_BMK.73952 |  | sbi-miR169c |
| Algae_064-1_Unigene_BMK.7414 |  | osa-miR168a-5p |
| Algae_064-1_Unigene_BMK.74819 |  | sbi-miR169c |
| Algae_064-1_Unigene_BMK.74973 | DNA polymerase alpha-associated DNA helicase A OS=Saccharomyces cerevisiae GN=HCS1 PE=1 SV=1 | stu-miR171b-3p |
| Algae_064-1_Unigene_BMK.75172 | Flocculation protein FLO11 OS=Saccharomyces cerevisiae GN=MUC1 PE=1 SV=2 | aca-miR-5p-43924 |
| Algae_064-1_Unigene_BMK.75346 |  | aca-miR-5p-43924 |
| Algae_064-1_Unigene_BMK.7546 | Epstein-Barr nuclear antigen 1 OS=Epstein-Barr virus (strain GD1) GN=EBNA1 PE=1 SV=1 | osa-miR168a-5p |
| Algae_064-1_Unigene_BMK.755 | Tubulin-specific chaperone B OS=Dictyostelium discoideum GN=tbcb PE=3 SV=1 | tae-miR159a |
| Algae_064-1_Unigene_BMK.75584 | Basic proline-rich protein OS=Sus scrofa PE=1 SV=2 | sbi-miR169c |
| Algae_064-1_Unigene_BMK.75761 | Pentatricopeptide repeat-containing protein At2g31400, chloroplastic OS=Arabidopsis thaliana GN=At2g31400 PE=2 SV=1 | sbi-miR169c |
| Algae_064-1_Unigene_BMK.75842 |  | aca-miR-3p-456915 |
| Algae_064-1_Unigene_BMK.76015 | Basic proline-rich protein OS=Sus scrofa PE=1 SV=2 | stu-miR171b-3p |
| Algae_064-1_Unigene_BMK.762 | CCR4-NOT transcription complex subunit 1 OS=Mus musculus GN=Cnot1 PE=1 SV=2 | osa-miR168a-5p |
| Algae_064-1_Unigene_BMK.76342 | Vegetative cell wall protein gp1 OS=Chlamydomonas reinhardtii GN=GP1 PE=2 SV=1 | aca-miR-5p-43924 |
| Algae_064-1_Unigene_BMK.76393 | Serine/arginine-rich splicing factor 2 OS=Gallus gallus GN=SRSF2 PE=2 SV=1 | aca-miR-5p-43924 |
| Algae_064-1_Unigene_BMK.7654 | Transmembrane protein 144 homolog A OS=Dictyostelium discoideum GN=tmem144A PE=3 SV=1 | aca-miR-3p-456915 |
| Algae_064-1_Unigene_BMK.766 |  | sbi-miR169c |
| Algae_064-1_Unigene_BMK.7673 | CDK5 regulatory subunit-associated protein 1-like 1 OS=Xenopus tropicalis GN=cdkal1 PE=2 SV=1 | osa-miR168a-5p |
| Algae_064-1_Unigene_BMK.77026 | Putative serine/threonine-protein kinase YPL150W OS=Saccharomyces cerevisiae GN=YPL150W PE=1 SV=1 | sbi-miR169c |
| Algae_064-1_Unigene_BMK.77093 | Ras-related protein Rab-5C OS=Gallus gallus GN=RAB5C PE=1 SV=1 | bdi-miR7732-3p_L-1_1ss11GC |
| Algae_064-1_Unigene_BMK.7719 |  | osa-miR168a-5p |
| Algae_064-1_Unigene_BMK.77362 | Long-chain fatty acid transport protein 1 OS=Rattus norvegicus GN=Slc27a1 PE=2 SV=1 | sbi-miR169c |
| Algae_064-1_Unigene_BMK.77497 |  | stu-miR171b-3p |
| Algae_064-1_Unigene_BMK.77718 | Cysteine string protein OS=Drosophila melanogaster GN=Csp PE=1 SV=1 | sbi-miR169c |
| Algae_064-1_Unigene_BMK.78527 | Abhydrolase domain-containing protein FAM108C1 OS=Danio rerio GN=fam108c1 PE=2 SV=1 | sbi-miR169c |
| Algae_064-1_Unigene_BMK.78650 | Proline-rich receptor-like protein kinase PERK13 OS=Arabidopsis thaliana GN=PERK13 PE=2 SV=1 | osa-miR168a-5p |
| Algae_064-1_Unigene_BMK.78651 |  | aca-miR-5p-43924 |
| Algae_064-1_Unigene_BMK.78682 |  | aca-miR-5p-43924 |
| Algae_064-1_Unigene_BMK.78740 |  | sbi-miR169c |
| Algae_064-1_Unigene_BMK.78917 | BEN domain-containing protein 4 OS=Mus musculus GN=Bend4 PE=3 SV=2 | osa-miR168a-5p |
| Algae_064-1_Unigene_BMK.7910 | Delta(14)-sterol reductase OS=Dictyostelium discoideum GN=erg24 PE=3 SV=1 | zma-miR529-5p |
| Algae_064-1_Unigene_BMK.79912 | Thioredoxin F, chloroplastic OS=Oryza sativa subsp. japonica GN=Os01g0913000 PE=2 SV=1 | osa-miR168a-5p |
| Algae_064-1_Unigene_BMK.8026 |  | aca-miR-5p-43924 |
| Algae_064-1_Unigene_BMK.8027 | Serine/arginine repetitive matrix protein 1 OS=Homo sapiens GN=SRRM1 PE=1 SV=2 | sbi-miR169c |
| Algae_064-1_Unigene_BMK.8030 | Polycystic kidney disease 2-like 2 protein OS=Mus musculus GN=Pkd2l2 PE=2 SV=1 | osa-miR168a-5p |
| Algae_064-1_Unigene_BMK.80616 | Deneddylase UL36 OS=Human herpesvirus 2 (strain HG52) GN=UL36 PE=3 SV=1 | aca-miR-3p-456915 |
| Algae_064-1_Unigene_BMK.8072 | Translation initiation factor IF-2 OS=Frankia sp. (strain EAN1pec) GN=infB PE=3 SV=1 | osa-miR168a-5p |
| Algae_064-1_Unigene_BMK.80887 | Glycine dehydrogenase [decarboxylating], mitochondrial OS=Solanum tuberosum GN=GDCSP PE=2 SV=1 | aca-miR-3p-456915 |
| Algae_064-1_Unigene_BMK.8107 | Salivary glue protein Sgs-3 OS=Drosophila erecta GN=Sgs3 PE=2 SV=3 | stu-miR171b-3p |
| Algae_064-1_Unigene_BMK.81442 | Translation initiation factor IF-2 OS=Mycobacterium gilvum (strain PYR-GCK) GN=infB PE=3 SV=1 | sbi-miR169c |
| Algae_064-1_Unigene_BMK.8155 | Uncharacterized protein DKFZp434B061 OS=Homo sapiens PE=2 SV=2 | sbi-miR169c |
| Algae_064-1_Unigene_BMK.81663 | Translation initiation factor IF-2 OS=Acidothermus cellulolyticus (strain ATCC 43068 / 11B) GN=infB PE=3 SV=1 | aca-miR-5p-43924 |
| Algae_064-1_Unigene_BMK.817 | Bifunctional dihydrofolate reductase-thymidylate synthase 2 OS=Arabidopsis thaliana GN=THY-2 PE=2 SV=2 | aca-miR-3p-456915 |
| Algae_064-1_Unigene_BMK.82029 | Serine/threonine-protein phosphatase 2A catalytic subunit A OS=Dictyostelium discoideum GN=pho2a PE=1 SV=1 | zma-miR529-5p |
| Algae_064-1_Unigene_BMK.8217 | Protein PROLIFERA OS=Arabidopsis thaliana GN=PRL PE=1 SV=2 | sbi-miR169c |
| Algae_064-1_Unigene_BMK.82476 | Translation initiation factor IF-2 OS=Frankia sp. (strain CcI3) GN=infB PE=3 SV=1 | stu-miR171b-3p |
| Algae_064-1_Unigene_BMK.82603 |  | sbi-miR169c |
| Algae_064-1_Unigene_BMK.82856 |  | aca-miR-5p-43924 |
| Algae_064-1_Unigene_BMK.82929 |  | zma-miR529-5p |
| Algae_064-1_Unigene_BMK.8307 | Fatty acid desaturase 2 OS=Macaca fascicularis GN=FADS2 PE=2 SV=1 | zma-miR529-5p |
| Algae_064-1_Unigene_BMK.8328 | Elongation of very long chain fatty acids protein 2 OS=Mus musculus GN=Elovl2 PE=2 SV=1 | aca-miR-3p-456915 |
| Algae_064-1_Unigene_BMK.83383 | Leucine-rich repeat-containing protein 57 OS=Danio rerio GN=lrrc57 PE=2 SV=1 | osa-miR168a-5p |
| Algae_064-1_Unigene_BMK.8375 | Caltractin ICL1e OS=Paramecium tetraurelia GN=Icl1e PE=3 SV=1 | bdi-miR7732-3p_L-1_1ss11GC |
| Algae_064-1_Unigene_BMK.84013 |  | zma-miR529-5p |
| Algae_064-1_Unigene_BMK.84110 | Acyl-coenzyme A thioesterase 10, mitochondrial OS=Mus musculus GN=Acot10 PE=2 SV=1 | osa-miR2876-3p_R+1 |
| Algae_064-1_Unigene_BMK.84239 | Serine/arginine repetitive matrix protein 2 OS=Mus musculus GN=Srrm2 PE=1 SV=2 | osa-miR168a-5p |
| Algae_064-1_Unigene_BMK.84294 |  | osa-miR168a-5p |
| Algae_064-1_Unigene_BMK.8473 | Serine/arginine repetitive matrix protein 2 OS=Homo sapiens GN=SRRM2 PE=1 SV=2 | sbi-miR169c |
| Algae_064-1_Unigene_BMK.8500 |  | osa-miR168a-5p |
| Algae_064-1_Unigene_BMK.85148 | Protein NLRC3 OS=Homo sapiens GN=NLRC3 PE=2 SV=2 | aca-miR-3p-456915 |
| Algae_064-1_Unigene_BMK.85309 | UPF0061 protein R00982 OS=Rhizobium meliloti GN=R00982 PE=3 SV=1 | aca-miR-5p-43924 |
| Algae_064-1_Unigene_BMK.8531 | Serine/arginine repetitive matrix protein 2 OS=Mus musculus GN=Srrm2 PE=1 SV=2 | aca-miR-3p-456915 |
| Algae_064-1_Unigene_BMK.8571 | Peptidyl-prolyl cis-trans isomerase CYP19-1 OS=Arabidopsis thaliana GN=CYP19-1 PE=1 SV=1 | zma-miR529-5p |
| Algae_064-1_Unigene_BMK.86283 |  | aca-miR-5p-43924 |
| Algae_064-1_Unigene_BMK.86324 | Pericentrin OS=Mus musculus GN=Pcnt PE=1 SV=2 | aca-miR-5p-43924 |
| Algae_064-1_Unigene_BMK.86367 |  | bdi-miR7732-3p_L-1_1ss11GC |
| Algae_064-1_Unigene_BMK.8673 | 60S ribosomal protein L13-2 OS=Brassica napus PE=2 SV=1 | sbi-miR169c |
| Algae_064-1_Unigene_BMK.8727 | Probable dimethyladenosine transferase OS=Caenorhabditis elegans GN=E02H1.1 PE=2 SV=2 | aca-miR-3p-456915 |
| Algae_064-1_Unigene_BMK.87292 | Kelch-like protein 18 OS=Homo sapiens GN=KLHL18 PE=2 SV=3 | sbi-miR169c |
| Algae_064-1_Unigene_BMK.8742 | Phosphorylated CTD-interacting factor 1 OS=Homo sapiens GN=PCIF1 PE=1 SV=1 | osa-miR2876-3p_R+1 |
| Algae_064-1_Unigene_BMK.87987 |  | sbi-miR169c |
| Algae_064-1_Unigene_BMK.882 | Translation initiation factor IF-2 OS=Nocardia farcinica GN=infB PE=3 SV=2 | aca-miR-3p-456915 |
| Algae_064-1_Unigene_BMK.88331 | Serine/arginine repetitive matrix protein 1 OS=Gallus gallus GN=SRRM1 PE=2 SV=1 | sbi-miR169c |
| Algae_064-1_Unigene_BMK.88921 | Uncharacterized protein DDB_G0271670 OS=Dictyostelium discoideum GN=DDB_G0271670 PE=4 SV=1 | stu-miR171b-3p |
| Algae_064-1_Unigene_BMK.8899 |  | stu-miR171b-3p |
| Algae_064-1_Unigene_BMK.8942 | Mucin-19 OS=Mus musculus GN=Muc19 PE=2 SV=2 | sbi-miR169c |
| Algae_064-1_Unigene_BMK.8949 | Bromodomain-containing protein 4 OS=Mus musculus GN=Brd4 PE=1 SV=1 | zma-miR529-5p |
| Algae_064-1_Unigene_BMK.89764 |  | osa-miR2876-3p_R+1 |
| Algae_064-1_Unigene_BMK.9 | BR serine/threonine-protein kinase 1 OS=Mus musculus GN=Brsk1 PE=1 SV=1 | sbi-miR169c |
| Algae_064-1_Unigene_BMK.90075 | Vegetative cell wall protein gp1 OS=Chlamydomonas reinhardtii GN=GP1 PE=2 SV=1 | sbi-miR169c |
| Algae_064-1_Unigene_BMK.90688 |  | aca-miR-3p-456915 |
| Algae_064-1_Unigene_BMK.9090 | AP-1 complex subunit mu-2 OS=Bos taurus GN=AP1M2 PE=1 SV=3 | osa-miR168a-5p |
| Algae_064-1_Unigene_BMK.9107 | Fatty aldehyde dehydrogenase OS=Rattus norvegicus GN=Aldh3a2 PE=1 SV=1 | osa-miR168a-5p |
| Algae_064-1_Unigene_BMK.9117 | Enoyl-[acyl-carrier-protein] reductase [NADH], chloroplastic OS=Brassica napus PE=1 SV=2 | sbi-miR169c |
| Algae_064-1_Unigene_BMK.91206 | Thymidylate synthase OS=Lactobacillus brevis (strain ATCC 367 / JCM 1170) GN=thyA PE=3 SV=1 | aca-miR-3p-456915 |
| Algae_064-1_Unigene_BMK.91223 | Probable S-acyltransferase At2g14255 OS=Arabidopsis thaliana GN=At2g14255 PE=2 SV=2 | sbi-miR169c |
| Algae_064-1_Unigene_BMK.91330 | Tellurium resistance protein TerZ OS=Serratia marcescens GN=terZ PE=3 SV=1 | rgl-miR5139_L+3 |
| Algae_064-1_Unigene_BMK.91437 | Monoglyceride lipase OS=Homo sapiens GN=MGLL PE=1 SV=2 | aca-miR-3p-456915 |
| Algae_064-1_Unigene_BMK.91485 | Bacitracin synthase 3 OS=Bacillus licheniformis GN=bacC PE=3 SV=1 | aca-miR-3p-456915 |
| Algae_064-1_Unigene_BMK.91582 | Minichromosome maintenance protein 5 OS=Saccharomyces cerevisiae GN=MCM5 PE=1 SV=1 | zma-miR529-5p |
| Algae_064-1_Unigene_BMK.9233 | Serine/arginine repetitive matrix protein 2 OS=Homo sapiens GN=SRRM2 PE=1 SV=2 | stu-miR171b-3p |
| Algae_064-1_Unigene_BMK.9312 |  | bdi-miR7732-3p_L-1_1ss11GC |
| Algae_064-1_Unigene_BMK.935 | Mucin-5AC (Fragments) OS=Homo sapiens GN=MUC5AC PE=1 SV=3 | zma-miR529-5p |
| Algae_064-1_Unigene_BMK.9809 | Mucin-19 OS=Mus musculus GN=Muc19 PE=2 SV=2 | tae-miR159a |
| Algae_064-1_Unigene_BMK.9841 | Basic proline-rich protein OS=Sus scrofa PE=1 SV=2 | osa-miR168a-5p |
| Algae_064-1_Unigene_BMK.9914 | Protein ENL OS=Homo sapiens GN=MLLT1 PE=1 SV=2 | zma-miR529-5p |
| Algae_064-2_Unigene_BMK.10008 | Kelch repeat-containing protein kel-10 OS=Caenorhabditis elegans GN=kel-10 PE=2 SV=4 | osa-miR168a-5p |
| Algae_064-2_Unigene_BMK.1018 | Omega-6 fatty acid desaturase, endoplasmic reticulum OS=Brassica juncea PE=2 SV=1 | bdi-miR7732-3p_L-1_1ss11GC |
| Algae_064-2_Unigene_BMK.1031 | Serine/threonine-protein phosphatase 6 regulatory ankyrin repeat subunit C OS=Danio rerio GN=ankrd52 PE=2 SV=1 | aca-miR-5p-43924 |
| Algae_064-2_Unigene_BMK.10379 | Coiled-coil domain-containing protein 63 OS=Bos taurus GN=CCDC63 PE=2 SV=1 | stu-miR171b-3p |
| Algae_064-2_Unigene_BMK.10385 | Mucin-19 OS=Mus musculus GN=Muc19 PE=2 SV=2 | sbi-miR169c |
| Algae_064-2_Unigene_BMK.10466 | Uncharacterized glycosyltransferase MJ1607 OS=Methanocaldococcus jannaschii GN=MJ1607 PE=3 SV=1 | aca-miR-3p-456915 |
| Algae_064-2_Unigene_BMK.1051 | Serine hydroxymethyltransferase, mitochondrial OS=Pisum sativum PE=1 SV=1 | bdi-miR7732-3p_L-1_1ss11GC |
| Algae_064-2_Unigene_BMK.1059 | Alpha,alpha-trehalose-phosphate synthase [UDP-forming] 2 OS=Caenorhabditis elegans GN=tps-2 PE=2 SV=3 | stu-miR171b-3p |
| Algae_064-2_Unigene_BMK.10674 | Apolipoprotein D OS=Mus musculus GN=Apod PE=2 SV=1 | aca-miR-3p-456915 |
| Algae_064-2_Unigene_BMK.10709 | Basic proline-rich protein OS=Sus scrofa PE=1 SV=2 | sbi-miR169c |
| Algae_064-2_Unigene_BMK.10886 | Cleavage and polyadenylation specificity factor CPSF30 OS=Arabidopsis thaliana GN=CPSF30 PE=1 SV=1 | aca-miR-5p-43924 |
| Algae_064-2_Unigene_BMK.10913 |  | bdi-miR7732-3p_L-1_1ss11GC |
| Algae_064-2_Unigene_BMK.11023 |  | aca-miR-5p-43924 |
| Algae_064-2_Unigene_BMK.11033 | Signal peptide, CUB and EGF-like domain-containing protein 1 OS=Homo sapiens GN=SCUBE1 PE=1 SV=3 | rgl-miR5139_L+3 |
| Algae_064-2_Unigene_BMK.11086 | Cyclin-dependent kinase 13 OS=Mus musculus GN=Cdk13 PE=1 SV=2 | osa-miR168a-5p |
| Algae_064-2_Unigene_BMK.11093 |  | bdi-miR7732-3p_L-1_1ss11GC |
| Algae_064-2_Unigene_BMK.11153 |  | sbi-miR169c |
| Algae_064-2_Unigene_BMK.11379 | Vegetative cell wall protein gp1 OS=Chlamydomonas reinhardtii GN=GP1 PE=2 SV=1 | sbi-miR169c |
| Algae_064-2_Unigene_BMK.11428 |  | zma-miR529-5p |
| Algae_064-2_Unigene_BMK.11538 |  | bdi-miR7732-3p_L-1_1ss11GC |
| Algae_064-2_Unigene_BMK.11622 | ABC transporter D family member 2, chloroplastic OS=Arabidopsis thaliana GN=ABCC2 PE=1 SV=1 | stu-miR171b-3p |
| Algae_064-2_Unigene_BMK.11841 | Translation initiation factor IF-2 OS=Mycobacterium gilvum (strain PYR-GCK) GN=infB PE=3 SV=1 | aca-miR-5p-43924 |
| Algae_064-2_Unigene_BMK.12003 | Uncharacterized protein LOC284861 OS=Homo sapiens PE=2 SV=1 | aca-miR-5p-43924 |
| Algae_064-2_Unigene_BMK.12020 | Uncharacterized protein ybeQ OS=Escherichia coli (strain K12) GN=ybeQ PE=4 SV=2 | aca-miR-3p-456915 |
| Algae_064-2_Unigene_BMK.12034 |  | rgl-miR5139_L+3 |
| Algae_064-2_Unigene_BMK.12072 | N amino acid transport system protein OS=Neurospora crassa GN=mtr PE=3 SV=2 | bdi-miR7732-3p_L-1_1ss11GC |
| Algae_064-2_Unigene_BMK.12371 | Probable serine/threonine-protein kinase dyrk2 OS=Dictyostelium discoideum GN=dyrk2 PE=3 SV=1 | tae-miR159a |
| Algae_064-2_Unigene_BMK.12463 |  | bdi-miR7732-3p_L-1_1ss11GC |
| Algae_064-2_Unigene_BMK.12516 | Phthiocerol synthesis polyketide synthase type I PpsA OS=Mycobacterium tuberculosis GN=ppsA PE=3 SV=2 | sbi-miR169c |
| Algae_064-2_Unigene_BMK.12598 | Inner membrane protein yhjX OS=Escherichia coli (strain K12) GN=yhjX PE=1 SV=1 | aca-miR-3p-456915 |
| Algae_064-2_Unigene_BMK.12653 | Serine/arginine repetitive matrix protein 3 OS=Homo sapiens GN=SRRM3 PE=2 SV=4 | stu-miR171b-3p |
| Algae_064-2_Unigene_BMK.12679 | Cytochrome P450 86A2 OS=Arabidopsis thaliana GN=CYP86A2 PE=2 SV=1 | zma-miR529-5p |
| Algae_064-2_Unigene_BMK.12767 | Mucin-5AC (Fragments) OS=Homo sapiens GN=MUC5AC PE=1 SV=3 | sbi-miR169c |
| Algae_064-2_Unigene_BMK.1279 |  | sbi-miR169c |
| Algae_064-2_Unigene_BMK.12869 |  | aca-miR-5p-43924 |
| Algae_064-2_Unigene_BMK.12888 | Protein terminal ear1 homolog OS=Oryza sativa subsp. japonica GN=PLA2 PE=2 SV=1 | osa-miR168a-5p |
| Algae_064-2_Unigene_BMK.12917 | Glycogen phosphorylase 2 OS=Dictyostelium discoideum GN=glpD PE=1 SV=2 | rgl-miR5139_L+3 |
| Algae_064-2_Unigene_BMK.13028 |  | stu-miR171b-3p |
| Algae_064-2_Unigene_BMK.1324 | Proline-rich proteoglycan 2 OS=Rattus norvegicus GN=Prpg2 PE=1 SV=2 | sbi-miR169c |
| Algae_064-2_Unigene_BMK.13271 | UPF0317 protein KRH_21160 OS=Kocuria rhizophila (strain ATCC 9341 / DSM 348 / NBRC 103217 / DC2201) GN=KRH_21160 PE=3 SV=1 | aca-miR-5p-43924 |
| Algae_064-2_Unigene_BMK.13420 | Aflatoxin B1 aldehyde reductase member 2 OS=Mus musculus GN=Akr7a2 PE=1 SV=2 | sbi-miR169c |
| Algae_064-2_Unigene_BMK.13469 | Calcium-dependent protein kinase 2 OS=Plasmodium falciparum (isolate K1 / Thailand) GN=CPK2 PE=1 SV=3 | rgl-miR5139_L+3 |
| Algae_064-2_Unigene_BMK.13489 | Eukaryotic translation initiation factor 4 gamma OS=Schizosaccharomyces pombe GN=tif471 PE=1 SV=1 | stu-miR171b-3p |
| Algae_064-2_Unigene_BMK.13509 | Uroporphyrinogen decarboxylase OS=Salinibacter ruber (strain DSM 13855 / M31) GN=hemE PE=3 SV=1 | aca-miR-3p-456915 |
| Algae_064-2_Unigene_BMK.13519 | Mucin-5B OS=Homo sapiens GN=MUC5B PE=1 SV=3 | bdi-miR7732-3p_L-1_1ss11GC |
| Algae_064-2_Unigene_BMK.13598 |  | osa-miR168a-5p |
| Algae_064-2_Unigene_BMK.13675 | Peroxisomal hydratase-dehydrogenase-epimerase OS=Neurospora crassa GN=fox-2 PE=1 SV=1 | zma-miR529-5p |
| Algae_064-2_Unigene_BMK.13937 | Ribulose bisphosphate carboxylase small chain A, chloroplastic OS=Oryza sativa subsp. japonica GN=RBCS-A PE=2 SV=2 | zma-miR529-5p |
| Algae_064-2_Unigene_BMK.13990 |  | aca-miR-5p-43924 |
| Algae_064-2_Unigene_BMK.13997 | Uncharacterized protein C10orf95 OS=Homo sapiens GN=C10orf95 PE=2 SV=1 | rgl-miR5139_L+3 |
| Algae_064-2_Unigene_BMK.14049 | 60 kDa lysophospholipase OS=Rattus norvegicus GN=Aspg PE=1 SV=1 | osa-miR168a-5p |
| Algae_064-2_Unigene_BMK.1407 |  | aca-miR-3p-456915 |
| Algae_064-2_Unigene_BMK.14193 | Acid ceramidase OS=Macaca fascicularis GN=ASAH1 PE=2 SV=1 | bdi-miR7732-3p_L-1_1ss11GC |
| Algae_064-2_Unigene_BMK.14253 | 3 beta-hydroxysteroid dehydrogenase/Delta 5-->4-isomerase OS=Mycobacterium tuberculosis GN=MT1137 PE=1 SV=1 | osa-miR168a-5p |
| Algae_064-2_Unigene_BMK.14345 |  | aca-miR-5p-43924 |
| Algae_064-2_Unigene_BMK.14349 | RNA-binding protein 12 OS=Macaca mulatta GN=RBM12 PE=2 SV=1 | osa-miR2876-3p_R+1 |
| Algae_064-2_Unigene_BMK.14415 | 60S ribosomal protein L22 OS=Drosophila melanogaster GN=RpL22 PE=1 SV=2 | sbi-miR169c |
| Algae_064-2_Unigene_BMK.14497 |  | tae-miR159a |
| Algae_064-2_Unigene_BMK.14563 | Transmembrane protein 144 homolog A OS=Dictyostelium discoideum GN=tmem144A PE=3 SV=1 | rgl-miR5139_L+3 |
| Algae_064-2_Unigene_BMK.14725 | Serine/arginine repetitive matrix protein 2 OS=Homo sapiens GN=SRRM2 PE=1 SV=2 | aca-miR-5p-43924 |
| Algae_064-2_Unigene_BMK.14778 | Uncharacterized membrane protein STKORF319 OS=Myxococcus xanthus PE=3 SV=1 | sbi-miR169c |
| Algae_064-2_Unigene_BMK.14805 | Nucleolar and coiled-body phosphoprotein 1 OS=Rattus norvegicus GN=Nolc1 PE=1 SV=1 | stu-miR171b-3p |
| Algae_064-2_Unigene_BMK.1492 |  | sbi-miR169c |
| Algae_064-2_Unigene_BMK.15081 |  | aca-miR-5p-43924 |
| Algae_064-2_Unigene_BMK.15104 | Basic proline-rich protein OS=Sus scrofa PE=1 SV=2 | sbi-miR169c |
| Algae_064-2_Unigene_BMK.15126 | Uncharacterized protein DKFZp434B061 OS=Homo sapiens PE=2 SV=2 | aca-miR-3p-456915 |
| Algae_064-2_Unigene_BMK.15203 | Immunoglobulin-binding protein 1 OS=Homo sapiens GN=IGBP1 PE=1 SV=1 | aca-miR-5p-43924 |
| Algae_064-2_Unigene_BMK.15283 | Sterol 3-beta-glucosyltransferase OS=Yarrowia lipolytica GN=ATG26 PE=3 SV=3 | tae-miR159a |
| Algae_064-2_Unigene_BMK.15401 | Uncharacterized protein RSN1 OS=Saccharomyces cerevisiae GN=RSN1 PE=1 SV=1 | aca-miR-5p-43924 |
| Algae_064-2_Unigene_BMK.15604 | Oryzain alpha chain OS=Oryza sativa subsp. japonica GN=Os04g0650000 PE=1 SV=2 | aca-miR-5p-43924 |
| Algae_064-2_Unigene_BMK.15777 | D-aminoacylase OS=Alcaligenes xylosoxydans xylosoxydans GN=dan PE=1 SV=3 | bdi-miR7732-3p_L-1_1ss11GC |
| Algae_064-2_Unigene_BMK.1608 | Transketolase OS=Nostoc sp. (strain PCC 7120 / UTEX 2576) GN=tkt PE=1 SV=1 | aca-miR-3p-456915 |
| Algae_064-2_Unigene_BMK.16163 | CMP-sialic acid transporter OS=Mus musculus GN=Slc35a1 PE=1 SV=1 | aca-miR-5p-43924 |
| Algae_064-2_Unigene_BMK.16204 | Uncharacterized protein LOC284861 OS=Homo sapiens PE=2 SV=1 | aca-miR-5p-43924 |
| Algae_064-2_Unigene_BMK.16219 | Translation initiation factor IF-2 OS=Frankia alni (strain ACN14a) GN=infB PE=3 SV=1 | osa-miR168a-5p |
| Algae_064-2_Unigene_BMK.16253 | Shaker-related potassium channel tsha2 OS=Oncorhynchus mykiss PE=2 SV=1 | sbi-miR169c |
| Algae_064-2_Unigene_BMK.16272 | Chaperone protein dnaK2 OS=Synechococcus elongatus (strain PCC 7942) GN=dnaK2 PE=3 SV=2 | osa-miR168a-5p |
| Algae_064-2_Unigene_BMK.16301 | Formin-like protein 20 OS=Arabidopsis thaliana GN=FH20 PE=2 SV=2 | stu-miR171b-3p |
| Algae_064-2_Unigene_BMK.1637 | Proline-rich receptor-like protein kinase PERK14 OS=Arabidopsis thaliana GN=PERK14 PE=2 SV=1 | osa-miR168a-5p |
| Algae_064-2_Unigene_BMK.16387 | Cation channel sperm-associated protein 1 OS=Mus musculus GN=Catsper1 PE=1 SV=1 | zma-miR529-5p |
| Algae_064-2_Unigene_BMK.16502 | Serine/arginine repetitive matrix protein 2 OS=Homo sapiens GN=SRRM2 PE=1 SV=2 | sbi-miR169c |
| Algae_064-2_Unigene_BMK.16731 | Uncharacterized protein slr0889 OS=Synechocystis sp. (strain ATCC 27184 / PCC 6803 / N-1) GN=slr0889 PE=3 SV=1 | sbi-miR169c |
| Algae_064-2_Unigene_BMK.16747 | Serine/arginine repetitive matrix protein 2 OS=Homo sapiens GN=SRRM2 PE=1 SV=2 | zma-miR529-5p |
| Algae_064-2_Unigene_BMK.16754 | Prohibitin-1 OS=Saccharomyces cerevisiae GN=PHB1 PE=1 SV=2 | aca-miR-3p-456915 |
| Algae_064-2_Unigene_BMK.1708 | Delta(24)-sterol reductase OS=Rattus norvegicus GN=Dhcr24 PE=2 SV=2 | rgl-miR5139_L+3 |
| Algae_064-2_Unigene_BMK.17141 | Glycoprotein gp2 OS=Equine herpesvirus 1 (strain V592) GN=71 PE=3 SV=1 | aca-miR-5p-43924 |
| Algae_064-2_Unigene_BMK.17198 | Serine/arginine repetitive matrix protein 2 OS=Homo sapiens GN=SRRM2 PE=1 SV=2 | aca-miR-5p-43924 |
| Algae_064-2_Unigene_BMK.17274 | Protein NLRC3 OS=Homo sapiens GN=NLRC3 PE=2 SV=2 | sbi-miR169c |
| Algae_064-2_Unigene_BMK.17439 | Serine/arginine repetitive matrix protein 3 OS=Homo sapiens GN=SRRM3 PE=2 SV=4 | aca-miR-5p-43924 |
| Algae_064-2_Unigene_BMK.17499 | Probable ion channel POLLUX OS=Arabidopsis thaliana GN=At5g49960 PE=2 SV=1 | osa-miR2876-3p_R+1 |
| Algae_064-2_Unigene_BMK.17521 | Serine/arginine repetitive matrix protein 2 OS=Homo sapiens GN=SRRM2 PE=1 SV=2 | zma-miR529-5p |
| Algae_064-2_Unigene_BMK.17523 | Uncharacterized protein BHLF1 OS=Epstein-Barr virus (strain B95-8) GN=BHLF1 PE=4 SV=1 | bdi-miR7732-3p_L-1_1ss11GC |
| Algae_064-2_Unigene_BMK.17666 |  | sbi-miR169c |
| Algae_064-2_Unigene_BMK.17704 | Uncharacterized protein LOC284861 OS=Homo sapiens PE=2 SV=1 | osa-miR168a-5p |
| Algae_064-2_Unigene_BMK.17711 | Uncharacterized protein DDB_G0271670 OS=Dictyostelium discoideum GN=DDB_G0271670 PE=4 SV=1 | stu-miR171b-3p |
| Algae_064-2_Unigene_BMK.17788 | Proline-rich protein 12 OS=Homo sapiens GN=PRR12 PE=1 SV=2 | osa-miR2876-3p_R+1 |
| Algae_064-2_Unigene_BMK.17915 | Malonyl-CoA-acyl carrier protein transacylase, mitochondrial OS=Homo sapiens GN=MCAT PE=1 SV=2 | aca-miR-3p-456915 |
| Algae_064-2_Unigene_BMK.1828 | TVP38/TMEM64 family membrane protein slr0305 OS=Synechocystis sp. (strain ATCC 27184 / PCC 6803 / N-1) GN=slr0305 PE=3 SV=1 | aca-miR-3p-456915 |
| Algae_064-2_Unigene_BMK.18428 | Transmembrane protein 231 OS=Bos taurus GN=TMEM231 PE=2 SV=1 | zma-miR529-5p |
| Algae_064-2_Unigene_BMK.18765 | Putative uncharacterized protein DDB_G0271974 OS=Dictyostelium discoideum GN=DDB_G0271974 PE=4 SV=1 | aca-miR-3p-456915 |
| Algae_064-2_Unigene_BMK.18962 | Arsenite resistance protein ArsB OS=Bacillus subtilis GN=arsB PE=3 SV=2 | aca-miR-5p-43924 |
| Algae_064-2_Unigene_BMK.19050 | Translation initiation factor IF-2 OS=Streptomyces griseus subsp. griseus (strain JCM 4626 / NBRC 13350) GN=infB PE=3 SV=1 | zma-miR529-5p |
| Algae_064-2_Unigene_BMK.1914 | Uncharacterized protein PA3753 OS=Pseudomonas aeruginosa GN=PA3753 PE=3 SV=2 | aca-miR-3p-456915 |
| Algae_064-2_Unigene_BMK.1919 | Translation initiation factor IF-2 OS=Streptomyces coelicolor GN=infB PE=3 SV=1 | zma-miR529-5p |
| Algae_064-2_Unigene_BMK.19224 |  | osa-miR168a-5p |
| Algae_064-2_Unigene_BMK.19241 | Guanine deaminase OS=Bacillus subtilis GN=guaD PE=1 SV=1 | aca-miR-5p-43924 |
| Algae_064-2_Unigene_BMK.19355 |  | osa-miR168a-5p |
| Algae_064-2_Unigene_BMK.19403 |  | stu-miR171b-3p |
| Algae_064-2_Unigene_BMK.19523 | Nucleolar protein 6 OS=Homo sapiens GN=NOL6 PE=1 SV=2 | aca-miR-5p-43924 |
| Algae_064-2_Unigene_BMK.19578 | Kinesin-like protein KIF2A OS=Pongo abelii GN=KIF2A PE=2 SV=1 | zma-miR529-5p |
| Algae_064-2_Unigene_BMK.19593 | cGMP-dependent protein kinase, isozyme 2 forms cD5/T2 OS=Drosophila melanogaster GN=for PE=2 SV=3 | zma-miR529-5p |
| Algae_064-2_Unigene_BMK.19691 | Serine/arginine repetitive matrix protein 2 OS=Mus musculus GN=Srrm2 PE=1 SV=2 | zma-miR529-5p |
| Algae_064-2_Unigene_BMK.19720 | MHC class II transactivator OS=Homo sapiens GN=CIITA PE=1 SV=3 | sbi-miR169c |
| Algae_064-2_Unigene_BMK.19759 | Serine/arginine repetitive matrix protein 1 OS=Gallus gallus GN=SRRM1 PE=2 SV=1 | aca-miR-5p-43924 |
| Algae_064-2_Unigene_BMK.19922 | Probable xylitol oxidase OS=Streptomyces coelicolor GN=xyoA PE=1 SV=1 | sbi-miR169c |
| Algae_064-2_Unigene_BMK.20042 | Serine/arginine repetitive matrix protein 2 OS=Homo sapiens GN=SRRM2 PE=1 SV=2 | osa-miR168a-5p |
| Algae_064-2_Unigene_BMK.20085 | Uncharacterized protein BHLF1 OS=Epstein-Barr virus (strain B95-8) GN=BHLF1 PE=4 SV=1 | stu-miR171b-3p |
| Algae_064-2_Unigene_BMK.20122 | Cyclin-T1-3 OS=Oryza sativa subsp. japonica GN=CYCT1-3 PE=3 SV=2 | osa-miR168a-5p |
| Algae_064-2_Unigene_BMK.20139 | Serine/arginine repetitive matrix protein 1 OS=Mus musculus GN=Srrm1 PE=1 SV=1 | aca-miR-5p-43924 |
| Algae_064-2_Unigene_BMK.20312 | Uncharacterized protein LOC284861 OS=Homo sapiens PE=2 SV=1 | aca-miR-5p-43924 |
| Algae_064-2_Unigene_BMK.20327 | Proline-rich protein 2 OS=Mus musculus GN=Prp2 PE=2 SV=2 | osa-miR168a-5p |
| Algae_064-2_Unigene_BMK.20469 |  | aca-miR-5p-43924 |
| Algae_064-2_Unigene_BMK.20487 | High molecular weight rubredoxin OS=Moorella thermoacetica (strain ATCC 39073) GN=hrb PE=1 SV=1 | sbi-miR169c |
| Algae_064-2_Unigene_BMK.20527 | 2-oxoglutarate dehydrogenase, mitochondrial OS=Dictyostelium discoideum GN=ogdh PE=3 SV=1 | aca-miR-5p-43924 |
| Algae_064-2_Unigene_BMK.20603 | Side tail fiber protein homolog from lambdoid prophage Rac OS=Escherichia coli (strain K12) GN=stfR PE=3 SV=2 | osa-miR168a-5p |
| Algae_064-2_Unigene_BMK.20617 | High mobility group protein B2 OS=Rattus norvegicus GN=Hmgb2 PE=2 SV=2 | stu-miR171b-3p |
| Algae_064-2_Unigene_BMK.2081 | Endothelin-converting enzyme 2 OS=Homo sapiens GN=ECE2 PE=1 SV=4 | aca-miR-5p-43924 |
| Algae_064-2_Unigene_BMK.20823 | Tubulin polyglutamylase TTLL4 OS=Mus musculus GN=Ttll4 PE=2 SV=3 | osa-miR168a-5p |
| Algae_064-2_Unigene_BMK.20887 | Probable E3 ubiquitin-protein ligase HERC2 OS=Drosophila melanogaster GN=HERC2 PE=1 SV=3 | aca-miR-5p-43924 |
| Algae_064-2_Unigene_BMK.20960 | Fatty acid desaturase OS=Bacillus subtilis GN=des PE=2 SV=1 | aca-miR-3p-456915 |
| Algae_064-2_Unigene_BMK.21059 | Soma ferritin OS=Lymnaea stagnalis PE=2 SV=2 | sbi-miR169c |
| Algae_064-2_Unigene_BMK.21138 | Probable protein phosphatase 2C 72 OS=Arabidopsis thaliana GN=At5g26010 PE=2 SV=2 | aca-miR-3p-456915 |
| Algae_064-2_Unigene_BMK.21193 |  | aca-miR-3p-456915 |
| Algae_064-2_Unigene_BMK.21272 | Mucin-5B OS=Homo sapiens GN=MUC5B PE=1 SV=3 | aca-miR-3p-456915 |
| Algae_064-2_Unigene_BMK.21524 | Sodium/hydrogen exchanger 8 OS=Arabidopsis thaliana GN=NHX8 PE=2 SV=1 | sbi-miR169c |
| Algae_064-2_Unigene_BMK.21609 | Two pore calcium channel protein 1 OS=Oryza sativa subsp. japonica GN=TPC1 PE=1 SV=2 | rgl-miR5139_L+3 |
| Algae_064-2_Unigene_BMK.21772 | Serine/arginine repetitive matrix protein 2 OS=Homo sapiens GN=SRRM2 PE=1 SV=2 | zma-miR529-5p |
| Algae_064-2_Unigene_BMK.21810 |  | aca-miR-3p-456915 |
| Algae_064-2_Unigene_BMK.2184 |  | aca-miR-5p-43924 |
| Algae_064-2_Unigene_BMK.21879 |  | sbi-miR169c |
| Algae_064-2_Unigene_BMK.21929 | Putative ankyrin repeat protein R840 OS=Acanthamoeba polyphaga mimivirus GN=MIMI_R840 PE=4 SV=1 | aca-miR-3p-456915 |
| Algae_064-2_Unigene_BMK.2195 | Translation initiation factor IF-2 OS=Rhodococcus opacus (strain B4) GN=infB PE=3 SV=1 | zma-miR529-5p |
| Algae_064-2_Unigene_BMK.21966 | 2-oxoglutarate dehydrogenase, mitochondrial OS=Dictyostelium discoideum GN=ogdh PE=3 SV=1 | osa-miR168a-5p |
| Algae_064-2_Unigene_BMK.22083 | Calcium/calmodulin-dependent protein kinase OS=Emericella nidulans GN=cmkA PE=2 SV=2 | osa-miR2876-3p_R+1 |
| Algae_064-2_Unigene_BMK.22112 | Uncharacterized protein DDB_G0271670 OS=Dictyostelium discoideum GN=DDB_G0271670 PE=4 SV=1 | zma-miR529-5p |
| Algae_064-2_Unigene_BMK.22210 | Caldesmon OS=Gallus gallus GN=CALD1 PE=1 SV=2 | zma-miR529-5p |
| Algae_064-2_Unigene_BMK.22247 | 3-hydroxyanthranilate 3,4-dioxygenase OS=Rattus norvegicus GN=Haao PE=1 SV=2 | bdi-miR7732-3p_L-1_1ss11GC |
| Algae_064-2_Unigene_BMK.22327 | Uncharacterized protein DDB_G0271670 OS=Dictyostelium discoideum GN=DDB_G0271670 PE=4 SV=1 | osa-miR2876-3p_R+1 |
| Algae_064-2_Unigene_BMK.22405 | Methyltransferase-like protein 13 OS=Bos taurus GN=METTL13 PE=2 SV=1 | aca-miR-5p-43924 |
| Algae_064-2_Unigene_BMK.22523 | Formin-like protein 1 OS=Homo sapiens GN=FMNL1 PE=1 SV=3 | sbi-miR169c |
| Algae_064-2_Unigene_BMK.22886 |  | aca-miR-5p-43924 |
| Algae_064-2_Unigene_BMK.22923 | Fibroin heavy chain OS=Bombyx mori GN=FIBH PE=1 SV=4 | osa-miR168a-5p |
| Algae_064-2_Unigene_BMK.22991 |  | zma-miR529-5p |
| Algae_064-2_Unigene_BMK.23168 | Uncharacterized protein BHLF1 OS=Epstein-Barr virus (strain B95-8) GN=BHLF1 PE=4 SV=1 | sbi-miR169c |
| Algae_064-2_Unigene_BMK.23169 | Probable protease sohB OS=Shigella flexneri GN=sohB PE=3 SV=1 | aca-miR-5p-43924 |
| Algae_064-2_Unigene_BMK.23293 | PPPDE peptidase domain-containing protein 2 OS=Homo sapiens GN=PPPDE2 PE=1 SV=1 | aca-miR-5p-43924 |
| Algae_064-2_Unigene_BMK.23407 | 2,5-dichloro-2,5-cyclohexadiene-1,4-diol dehydrogenase OS=Pseudomonas paucimobilis GN=linX PE=3 SV=1 | osa-miR168a-5p |
| Algae_064-2_Unigene_BMK.23409 | Probable glucan 1,3-beta-glucosidase A OS=Neosartorya fischeri (strain ATCC 1020 / DSM 3700 / FGSC A1164 / NRRL 181) GN=exgA PE=3 SV=1 | aca-miR-3p-456915 |
| Algae_064-2_Unigene_BMK.23733 | Cyclin-U4-1 OS=Arabidopsis thaliana GN=CYCU4-1 PE=1 SV=1 | sbi-miR169c |
| Algae_064-2_Unigene_BMK.23793 | Putative sulfur deprivation response regulator OS=Chlamydomonas reinhardtii GN=SAC1 PE=2 SV=1 | aca-miR-3p-456915 |
| Algae_064-2_Unigene_BMK.23838 | Mucin-5AC (Fragments) OS=Homo sapiens GN=MUC5AC PE=1 SV=3 | aca-miR-5p-43924 |
| Algae_064-2_Unigene_BMK.24274 | Uncharacterized aarF domain-containing protein kinase 1 OS=Mus musculus GN=Adck1 PE=2 SV=1 | aca-miR-3p-456915 |
| Algae_064-2_Unigene_BMK.2454 | Epstein-Barr nuclear antigen 1 OS=Epstein-Barr virus (strain AG876) GN=EBNA1 PE=3 SV=1 | stu-miR171b-3p |
| Algae_064-2_Unigene_BMK.24554 | Multidrug and toxin extrusion protein 1 OS=Homo sapiens GN=SLC47A1 PE=1 SV=1 | aca-miR-5p-43924 |
| Algae_064-2_Unigene_BMK.24558 | Ankyrin-1 OS=Mus musculus GN=Ank1 PE=1 SV=2 | sbi-miR169c |
| Algae_064-2_Unigene_BMK.24587 | Fucoxanthin-chlorophyll a-c binding protein F, chloroplastic (Fragment) OS=Macrocystis pyrifera GN=FCPF PE=2 SV=1 | aca-miR-3p-456915 |
| Algae_064-2_Unigene_BMK.24631 | Hydrophobic protein LTI6B OS=Oryza sativa subsp. japonica GN=LTI6B PE=2 SV=1 | sbi-miR169c |
| Algae_064-2_Unigene_BMK.24651 |  | aca-miR-5p-43924 |
| Algae_064-2_Unigene_BMK.24696 |  | osa-miR168a-5p |
| Algae_064-2_Unigene_BMK.24742 |  | aca-miR-3p-456915 |
| Algae_064-2_Unigene_BMK.24759 | Uncharacterized serine-rich protein C215.13 OS=Schizosaccharomyces pombe GN=SPBC215.13 PE=1 SV=1 | sbi-miR169c |
| Algae_064-2_Unigene_BMK.24848 | Histone-lysine N-methyltransferase SETD1B OS=Mus musculus GN=Setd1b PE=2 SV=2 | aca-miR-5p-43924 |
| Algae_064-2_Unigene_BMK.25002 | Mucin-19 OS=Mus musculus GN=Muc19 PE=2 SV=2 | osa-miR168a-5p |
| Algae_064-2_Unigene_BMK.25389 | Putative nitric oxide synthase OS=Oryza sativa subsp. japonica GN=Os02g0104700 PE=3 SV=1 | aca-miR-5p-43924 |
| Algae_064-2_Unigene_BMK.25417 |  | sbi-miR169c |
| Algae_064-2_Unigene_BMK.25460 |  | sbi-miR169c |
| Algae_064-2_Unigene_BMK.25496 | Golgin candidate 6 OS=Arabidopsis thaliana GN=GC6 PE=1 SV=2 | stu-miR171b-3p |
| Algae_064-2_Unigene_BMK.25526 | Uncharacterized membrane protein C776.05 OS=Schizosaccharomyces pombe GN=SPBC776.05 PE=2 SV=1 | osa-miR168a-5p |
| Algae_064-2_Unigene_BMK.25626 | Flocculation protein FLO11 OS=Saccharomyces cerevisiae GN=MUC1 PE=1 SV=2 | stu-miR171b-3p |
| Algae_064-2_Unigene_BMK.25706 | Serine/arginine repetitive matrix protein 1 OS=Homo sapiens GN=SRRM1 PE=1 SV=2 | stu-miR171b-3p |
| Algae_064-2_Unigene_BMK.2571 | Calcium/calmodulin-dependent protein kinase type 1 OS=Caenorhabditis briggsae GN=cmk-1 PE=3 SV=3 | osa-miR168a-5p |
| Algae_064-2_Unigene_BMK.25837 |  | sbi-miR169c |
| Algae_064-2_Unigene_BMK.25860 | Translation initiation factor IF-2 OS=Mycobacterium sp. (strain MCS) GN=infB PE=3 SV=1 | sbi-miR169c |
| Algae_064-2_Unigene_BMK.25968 | Serine/arginine repetitive matrix protein 1 OS=Mus musculus GN=Srrm1 PE=1 SV=1 | zma-miR529-5p |
| Algae_064-2_Unigene_BMK.25978 | Regulator of nonsense transcripts 1 homolog OS=Drosophila melanogaster GN=Upf1 PE=1 SV=2 | zma-miR529-5p |
| Algae_064-2_Unigene_BMK.26039 | A-agglutinin anchorage subunit OS=Saccharomyces cerevisiae GN=AGA1 PE=1 SV=1 | osa-miR2876-3p_R+1 |
| Algae_064-2_Unigene_BMK.26132 | Mucin-5AC (Fragments) OS=Homo sapiens GN=MUC5AC PE=1 SV=3 | stu-miR171b-3p |
| Algae_064-2_Unigene_BMK.2630 | Ensconsin OS=Gallus gallus GN=MAP7 PE=2 SV=1 | rgl-miR5139_L+3 |
| Algae_064-2_Unigene_BMK.26301 | Acyl-protein thioesterase 1 OS=Schizosaccharomyces pombe GN=SPAC8E11.04c PE=2 SV=1 | sbi-miR169c |
| Algae_064-2_Unigene_BMK.26310 | Translation initiation factor IF-2 OS=Frankia sp. (strain EAN1pec) GN=infB PE=3 SV=1 | zma-miR529-5p |
| Algae_064-2_Unigene_BMK.26478 | Probable E3 ubiquitin-protein ligase MGRN1 OS=Danio rerio GN=mgrn1 PE=2 SV=1 | zma-miR529-5p |
| Algae_064-2_Unigene_BMK.2684 | Arogenate dehydratase/prephenate dehydratase 1, chloroplastic OS=Arabidopsis thaliana GN=ADT1 PE=1 SV=1 | stu-miR171b-3p |
| Algae_064-2_Unigene_BMK.2707 | Spore coat protein A OS=Bacillus subtilis GN=cotA PE=1 SV=4 | osa-miR168a-5p |
| Algae_064-2_Unigene_BMK.27090 | ABC transporter D family member 1 OS=Arabidopsis thaliana GN=ABCC1 PE=1 SV=1 | sbi-miR169c |
| Algae_064-2_Unigene_BMK.27135 | Translation initiation factor IF-2 OS=Frankia sp. (strain EAN1pec) GN=infB PE=3 SV=1 | bdi-miR7732-3p_L-1_1ss11GC |
| Algae_064-2_Unigene_BMK.2716 | DEAD-box ATP-dependent RNA helicase 14 OS=Arabidopsis thaliana GN=RH14 PE=1 SV=2 | aca-miR-5p-43924 |
| Algae_064-2_Unigene_BMK.27236 |  | stu-miR171b-3p |
| Algae_064-2_Unigene_BMK.27337 |  | aca-miR-5p-43924 |
| Algae_064-2_Unigene_BMK.27485 | Glycoprotein gp2 OS=Equine herpesvirus 1 (strain Ab4p) GN=EUs4 PE=4 SV=1 | bdi-miR7732-3p_L-1_1ss11GC |
| Algae_064-2_Unigene_BMK.27511 | Translation initiation factor IF-2 OS=Frankia alni (strain ACN14a) GN=infB PE=3 SV=1 | aca-miR-5p-43924 |
| Algae_064-2_Unigene_BMK.27663 |  | osa-miR168a-5p |
| Algae_064-2_Unigene_BMK.27763 | UPF0187 protein sll1024 OS=Synechocystis sp. (strain ATCC 27184 / PCC 6803 / N-1) GN=sll1024 PE=3 SV=2 | osa-miR168a-5p |
| Algae_064-2_Unigene_BMK.27776 | Proline-rich proteoglycan 2 OS=Rattus norvegicus GN=Prpg2 PE=1 SV=2 | aca-miR-3p-456915 |
| Algae_064-2_Unigene_BMK.27899 | Proline-rich receptor-like protein kinase PERK9 OS=Arabidopsis thaliana GN=PERK9 PE=2 SV=1 | osa-miR168a-5p |
| Algae_064-2_Unigene_BMK.28043 | Uncharacterized peptidase y4nA OS=Rhizobium sp. (strain NGR234) GN=NGR_a02410 PE=3 SV=1 | rgl-miR5139_L+3 |
| Algae_064-2_Unigene_BMK.28070 | Uncharacterized protein ydeI OS=Bacillus subtilis GN=ydeI PE=4 SV=1 | osa-miR2876-3p_R+1 |
| Algae_064-2_Unigene_BMK.28087 | Acyl-CoA thioesterase 2 OS=Escherichia coli (strain K12) GN=tesB PE=1 SV=2 | bdi-miR7732-3p_L-1_1ss11GC |
| Algae_064-2_Unigene_BMK.28168 |  | stu-miR171b-3p |
| Algae_064-2_Unigene_BMK.28320 | Uncharacterized protein DDB_G0271670 OS=Dictyostelium discoideum GN=DDB_G0271670 PE=4 SV=1 | aca-miR-5p-43924 |
| Algae_064-2_Unigene_BMK.28581 | Uncharacterized abhydrolase domain-containing protein DDB_G0269086 OS=Dictyostelium discoideum GN=DDB_G0269086 PE=1 SV=2 | stu-miR171b-3p |
| Algae_064-2_Unigene_BMK.2863 | Nitric oxide synthase-interacting protein OS=Homo sapiens GN=NOSIP PE=1 SV=1 | aca-miR-5p-43924 |
| Algae_064-2_Unigene_BMK.2877 | Stress-induced-phosphoprotein 1 OS=Macaca fascicularis GN=STIP1 PE=2 SV=1 | aca-miR-5p-43924 |
| Algae_064-2_Unigene_BMK.28809 | Palmitoyltransferase ZDHHC15 OS=Mus musculus GN=Zdhhc15 PE=1 SV=1 | aca-miR-3p-456915 |
| Algae_064-2_Unigene_BMK.2887 | Retinol dehydrogenase 12 OS=Mus musculus GN=Rdh12 PE=2 SV=1 | sbi-miR169c |
| Algae_064-2_Unigene_BMK.28875 | Probable inorganic phosphate transporter 1-10 OS=Oryza sativa subsp. japonica GN=PHT1-10 PE=2 SV=1 | osa-miR168a-5p |
| Algae_064-2_Unigene_BMK.28974 |  | sbi-miR169c |
| Algae_064-2_Unigene_BMK.29017 |  | zma-miR529-5p |
| Algae_064-2_Unigene_BMK.29143 | Pre-mRNA-splicing factor 38B OS=Rattus norvegicus GN=Prpf38b PE=2 SV=1 | zma-miR529-5p |
| Algae_064-2_Unigene_BMK.29180 | Aspartate beta-hydroxylase domain-containing protein 2 OS=Danio rerio GN=asphd2 PE=2 SV=1 | sbi-miR169c |
| Algae_064-2_Unigene_BMK.29212 | ABC transporter D family member 1 OS=Arabidopsis thaliana GN=ABCC1 PE=1 SV=1 | sbi-miR169c |
| Algae_064-2_Unigene_BMK.2922 | Histone-lysine N-methyltransferase, H3 lysine-79 specific OS=Ashbya gossypii (strain ATCC 10895 / CBS 109.51 / FGSC 9923 / NRRL Y-1056) GN=DOT1 PE=3 SV=1 | tae-miR159a |
| Algae_064-2_Unigene_BMK.29235 |  | sbi-miR169c |
| Algae_064-2_Unigene_BMK.29287 |  | sbi-miR169c |
| Algae_064-2_Unigene_BMK.2932 | Quinone oxidoreductase-like protein At1g23740, chloroplastic OS=Arabidopsis thaliana GN=At1g23740 PE=1 SV=2 | osa-miR168a-5p |
| Algae_064-2_Unigene_BMK.29378 | Serine/arginine repetitive matrix protein 3 OS=Homo sapiens GN=SRRM3 PE=2 SV=4 | aca-miR-5p-43924 |
| Algae_064-2_Unigene_BMK.29420 | Serine/arginine repetitive matrix protein 2 OS=Mus musculus GN=Srrm2 PE=1 SV=2 | aca-miR-5p-43924 |
| Algae_064-2_Unigene_BMK.29521 | Alpha-ketoglutarate-dependent sulfonate dioxygenase OS=Saccharomyces cerevisiae GN=JLP1 PE=2 SV=1 | aca-miR-3p-456915 |
| Algae_064-2_Unigene_BMK.29535 | Nephrocystin-3 OS=Mus musculus GN=Nphp3 PE=1 SV=1 | tae-miR159a |
| Algae_064-2_Unigene_BMK.29593 |  | rgl-miR5139_L+3 |
| Algae_064-2_Unigene_BMK.2973 | Uncharacterized protein BHLF1 OS=Epstein-Barr virus (strain B95-8) GN=BHLF1 PE=4 SV=1 | stu-miR171b-3p |
| Algae_064-2_Unigene_BMK.29875 | Serine/arginine repetitive matrix protein 1 OS=Gallus gallus GN=SRRM1 PE=2 SV=1 | stu-miR171b-3p |
| Algae_064-2_Unigene_BMK.2988 | Uncharacterized 35.5 kDa protein in transposon Tn4556 OS=Streptomyces fradiae PE=4 SV=1 | aca-miR-3p-456915 |
| Algae_064-2_Unigene_BMK.29977 | Pentatricopeptide repeat-containing protein At2g31400, chloroplastic OS=Arabidopsis thaliana GN=At2g31400 PE=2 SV=1 | aca-miR-5p-43924 |
| Algae_064-2_Unigene_BMK.30016 | Glycoprotein gp2 OS=Equine herpesvirus 1 (strain V592) GN=71 PE=3 SV=1 | rgl-miR5139_L+3 |
| Algae_064-2_Unigene_BMK.30157 | Uncharacterized abhydrolase domain-containing protein DDB_G0269086 OS=Dictyostelium discoideum GN=DDB_G0269086 PE=1 SV=2 | aca-miR-5p-43924 |
| Algae_064-2_Unigene_BMK.30217 | Putative protein TPRXL OS=Homo sapiens GN=TPRXL PE=5 SV=2 | sbi-miR169c |
| Algae_064-2_Unigene_BMK.30243 | Polypyrimidine tract-binding protein 1 OS=Bos taurus GN=PTBP1 PE=2 SV=1 | aca-miR-3p-456915 |
| Algae_064-2_Unigene_BMK.30244 |  | osa-miR168a-5p |
| Algae_064-2_Unigene_BMK.30584 |  | aca-miR-3p-456915 |
| Algae_064-2_Unigene_BMK.30592 | Casein kinase I isoform epsilon OS=Mus musculus GN=Csnk1e PE=1 SV=2 | sbi-miR169c |
| Algae_064-2_Unigene_BMK.30698 | Putative uncharacterized protein ENSP00000383309 OS=Homo sapiens PE=5 SV=3 | bdi-miR7732-3p_L-1_1ss11GC |
| Algae_064-2_Unigene_BMK.30844 | Translation initiation factor IF-2 OS=Streptomyces griseus subsp. griseus (strain JCM 4626 / NBRC 13350) GN=infB PE=3 SV=1 | osa-miR168a-5p |
| Algae_064-2_Unigene_BMK.30902 |  | aca-miR-5p-43924 |
| Algae_064-2_Unigene_BMK.30916 | Proline-rich protein HaeIII subfamily 1 OS=Mus musculus GN=Prh1 PE=2 SV=2 | aca-miR-3p-456915 |
| Algae_064-2_Unigene_BMK.31081 | Potassium/sodium hyperpolarization-activated cyclic nucleotide-gated channel 4 OS=Rattus norvegicus GN=Hcn4 PE=2 SV=1 | zma-miR529-5p |
| Algae_064-2_Unigene_BMK.31115 |  | zma-miR529-5p |
| Algae_064-2_Unigene_BMK.31169 |  | stu-miR171b-3p |
| Algae_064-2_Unigene_BMK.31312 | Cytochrome b5-related protein OS=Drosophila melanogaster GN=Cyt-b5-r PE=1 SV=2 | aca-miR-5p-43924 |
| Algae_064-2_Unigene_BMK.31387 | Mucin-19 OS=Mus musculus GN=Muc19 PE=2 SV=2 | osa-miR168a-5p |
| Algae_064-2_Unigene_BMK.31431 | Cysteine desulfurase OS=Dictyoglomus turgidum (strain Z-1310 / DSM 6724) GN=iscS PE=3 SV=1 | stu-miR171b-3p |
| Algae_064-2_Unigene_BMK.31519 | Uncharacterized protein LOC284861 OS=Homo sapiens PE=2 SV=1 | aca-miR-5p-43924 |
| Algae_064-2_Unigene_BMK.3152 | Protein KRI1 homolog OS=Danio rerio GN=kri1 PE=2 SV=1 | aca-miR-5p-43924 |
| Algae_064-2_Unigene_BMK.31554 |  | stu-miR171b-3p |
| Algae_064-2_Unigene_BMK.31569 | Ankyrin-1 OS=Homo sapiens GN=ANK1 PE=1 SV=3 | sbi-miR169c |
| Algae_064-2_Unigene_BMK.31702 | Eukaryotic translation initiation factor 4E OS=Candida albicans GN=TIF45 PE=3 SV=1 | rgl-miR5139_L+3 |
| Algae_064-2_Unigene_BMK.31894 | Translation initiation factor IF-2 OS=Frankia alni (strain ACN14a) GN=infB PE=3 SV=1 | osa-miR168a-5p |
| Algae_064-2_Unigene_BMK.32053 |  | aca-miR-3p-456915 |
| Algae_064-2_Unigene_BMK.32388 | N-acylethanolamine-hydrolyzing acid amidase OS=Rattus norvegicus GN=Naaa PE=1 SV=1 | osa-miR168a-5p |
| Algae_064-2_Unigene_BMK.32420 | Putative protein TPRXL OS=Homo sapiens GN=TPRXL PE=5 SV=2 | sbi-miR169c |
| Algae_064-2_Unigene_BMK.32561 | Proline-rich receptor-like protein kinase PERK2 OS=Arabidopsis thaliana GN=PERK2 PE=2 SV=3 | stu-miR171b-3p |
| Algae_064-2_Unigene_BMK.32581 | Uncharacterized protein LOC284861 OS=Homo sapiens PE=2 SV=1 | zma-miR529-5p |
| Algae_064-2_Unigene_BMK.32643 | Potassium voltage-gated channel subfamily D member 1 OS=Homo sapiens GN=KCND1 PE=1 SV=2 | sbi-miR169c |
| Algae_064-2_Unigene_BMK.32760 | Sodium channel protein type 11 subunit alpha OS=Rattus norvegicus GN=Scn11a PE=1 SV=1 | aca-miR-5p-43924 |
| Algae_064-2_Unigene_BMK.32833 |  | osa-miR168a-5p |
| Algae_064-2_Unigene_BMK.32910 | Serine/arginine repetitive matrix protein 2 OS=Homo sapiens GN=SRRM2 PE=1 SV=2 | sbi-miR169c |
| Algae_064-2_Unigene_BMK.32964 |  | zma-miR529-5p |
| Algae_064-2_Unigene_BMK.33062 | Serine/arginine repetitive matrix protein 1 OS=Pongo abelii GN=SRRM1 PE=2 SV=1 | aca-miR-5p-43924 |
| Algae_064-2_Unigene_BMK.33177 | Pentafunctional AROM polypeptide OS=Laccaria bicolor (strain S238N-H82) GN=LACBIDRAFT_233717 PE=3 SV=1 | aca-miR-5p-43924 |
| Algae_064-2_Unigene_BMK.33263 | COBW domain-containing protein 2 OS=Homo sapiens GN=CBWD2 PE=1 SV=1 | aca-miR-3p-456915 |
| Algae_064-2_Unigene_BMK.33388 |  | osa-miR168a-5p |
| Algae_064-2_Unigene_BMK.33405 | Translation initiation factor IF-2 OS=Frankia alni (strain ACN14a) GN=infB PE=3 SV=1 | aca-miR-5p-43924 |
| Algae_064-2_Unigene_BMK.3341 | Plectin OS=Rattus norvegicus GN=Plec PE=1 SV=2 | aca-miR-5p-43924 |
| Algae_064-2_Unigene_BMK.33461 |  | osa-miR168a-5p |
| Algae_064-2_Unigene_BMK.3360 | Mannitol 2-dehydrogenase OS=Aspergillus oryzae (strain ATCC 42149 / RIB 40) GN=AO090011000230 PE=3 SV=1 | stu-miR171b-3p |
| Algae_064-2_Unigene_BMK.33619 | Uncharacterized 24.1 kDa protein in LEF4-P33 intergenic region OS=Autographa californica nuclear polyhedrosis virus PE=4 SV=1 | sbi-miR169c |
| Algae_064-2_Unigene_BMK.33773 | Serine/arginine repetitive matrix protein 2 OS=Mus musculus GN=Srrm2 PE=1 SV=2 | sbi-miR169c |
| Algae_064-2_Unigene_BMK.33900 | Phytanoyl-CoA dioxygenase domain-containing protein 1 OS=Homo sapiens GN=PHYHD1 PE=1 SV=2 | osa-miR168a-5p |
| Algae_064-2_Unigene_BMK.3391 | Uncharacterized protein LOC284861 OS=Homo sapiens PE=2 SV=1 | sbi-miR169c |
| Algae_064-2_Unigene_BMK.33991 | Uncharacterized protein BHLF1 OS=Epstein-Barr virus (strain B95-8) GN=BHLF1 PE=4 SV=1 | aca-miR-5p-43924 |
| Algae_064-2_Unigene_BMK.33997 |  | aca-miR-3p-456915 |
| Algae_064-2_Unigene_BMK.34146 | Putative phosphoglycerate mutase DET1 OS=Saccharomyces cerevisiae GN=DET1 PE=1 SV=1 | aca-miR-3p-456915 |
| Algae_064-2_Unigene_BMK.3415 | Voltage-dependent L-type calcium channel subunit alpha-1D OS=Gallus gallus GN=CACNA1D PE=2 SV=1 | tae-miR159a |
| Algae_064-2_Unigene_BMK.34173 | Serine/arginine repetitive matrix protein 2 OS=Homo sapiens GN=SRRM2 PE=1 SV=2 | sbi-miR169c |
| Algae_064-2_Unigene_BMK.34188 | Uncharacterized protein DKFZp434B061 OS=Homo sapiens PE=2 SV=2 | stu-miR171b-3p |
| Algae_064-2_Unigene_BMK.34221 |  | aca-miR-3p-456915 |
| Algae_064-2_Unigene_BMK.34373 |  | aca-miR-5p-43924 |
| Algae_064-2_Unigene_BMK.3451 | Uncharacterized protein C24H6.11c OS=Schizosaccharomyces pombe GN=SPAC24H6.11c PE=2 SV=1 | rgl-miR5139_L+3 |
| Algae_064-2_Unigene_BMK.34611 | Lupus La protein homolog OS=Mus musculus GN=Ssb PE=2 SV=1 | aca-miR-5p-43924 |
| Algae_064-2_Unigene_BMK.34713 | Putative protein TPRXL OS=Homo sapiens GN=TPRXL PE=5 SV=2 | aca-miR-5p-43924 |
| Algae_064-2_Unigene_BMK.34853 | Probable E3 ubiquitin-protein ligase MYCBP2 OS=Mus musculus GN=Mycbp2 PE=1 SV=2 | sbi-miR169c |
| Algae_064-2_Unigene_BMK.35006 | Lon protease homolog, mitochondrial OS=Phaeodactylum tricornutum (strain CCAP 1055/1) GN=PHATRDRAFT_18202 PE=3 SV=1 | stu-miR171b-3p |
| Algae_064-2_Unigene_BMK.35079 | FAD-linked oxidoreductase DDB_G0289697 OS=Dictyostelium discoideum GN=DDB_G0289697 PE=2 SV=1 | tae-miR159a |
| Algae_064-2_Unigene_BMK.35110 | Serine/arginine repetitive matrix protein 2 OS=Homo sapiens GN=SRRM2 PE=1 SV=2 | sbi-miR169c |
| Algae_064-2_Unigene_BMK.35778 | Chaperone protein dnaJ OS=Burkholderia pseudomallei GN=dnaJ PE=3 SV=1 | zma-miR529-5p |
| Algae_064-2_Unigene_BMK.36093 | Uncharacterized protein DKFZp434B061 OS=Homo sapiens PE=2 SV=2 | osa-miR168a-5p |
| Algae_064-2_Unigene_BMK.36131 |  | sbi-miR169c |
| Algae_064-2_Unigene_BMK.36176 | Pyrimidine-specific ribonucleoside hydrolase rihA OS=Escherichia fergusonii (strain ATCC 35469 / DSM 13698 / CDC 0568-73) GN=rihA PE=3 SV=1 | aca-miR-5p-43924 |
| Algae_064-2_Unigene_BMK.36285 |  | bdi-miR7732-3p_L-1_1ss11GC |
| Algae_064-2_Unigene_BMK.36319 | Putative polyol transporter 1 OS=Arabidopsis thaliana GN=PLT1 PE=2 SV=1 | zma-miR529-5p |
| Algae_064-2_Unigene_BMK.36341 | Uncharacterized protein L662 OS=Acanthamoeba polyphaga mimivirus GN=MIMI_L662 PE=4 SV=1 | aca-miR-5p-43924 |
| Algae_064-2_Unigene_BMK.36358 | 1,3-beta-glucan synthase component FKS1 OS=Aspergillus niger (strain CBS 513.88 / FGSC A1513) GN=fksA PE=3 SV=1 | rgl-miR5139_L+3 |
| Algae_064-2_Unigene_BMK.36386 | EH domain-containing protein 1 OS=Rattus norvegicus GN=Ehd1 PE=1 SV=1 | bdi-miR7732-3p_L-1_1ss11GC |
| Algae_064-2_Unigene_BMK.36442 | C3 and PZP-like alpha-2-macroglobulin domain-containing protein 8 OS=Homo sapiens GN=CPAMD8 PE=1 SV=2 | sbi-miR169c |
| Algae_064-2_Unigene_BMK.36664 | Probable inorganic phosphate transporter 1-6 OS=Arabidopsis thaliana GN=PHT1-6 PE=2 SV=1 | aca-miR-3p-456915 |
| Algae_064-2_Unigene_BMK.36757 | Integumentary mucin C.1 (Fragment) OS=Xenopus laevis PE=2 SV=1 | stu-miR171b-3p |
| Algae_064-2_Unigene_BMK.36867 | Serine/arginine repetitive matrix protein 2 OS=Mus musculus GN=Srrm2 PE=1 SV=2 | rgl-miR5139_L+3 |
| Algae_064-2_Unigene_BMK.36979 | Transmembrane protein 65 OS=Homo sapiens GN=TMEM65 PE=1 SV=2 | aca-miR-5p-43924 |
| Algae_064-2_Unigene_BMK.37064 | Uncharacterized gene 73 protein OS=Alcelaphine herpesvirus 1 (strain C500) GN=73 PE=4 SV=1 | osa-miR168a-5p |
| Algae_064-2_Unigene_BMK.37370 | Dehydrogenase/reductase SDR family member on chromosome X OS=Homo sapiens GN=DHRSX PE=2 SV=2 | zma-miR529-5p |
| Algae_064-2_Unigene_BMK.374 | Uncharacterized protein BHLF1 OS=Epstein-Barr virus (strain B95-8) GN=BHLF1 PE=4 SV=1 | zma-miR529-5p |
| Algae_064-2_Unigene_BMK.37553 |  | stu-miR171b-3p |
| Algae_064-2_Unigene_BMK.37684 | Putative 5'(3')-deoxyribonucleotidase OS=Bordetella bronchiseptica GN=BB0433 PE=3 SV=1 | sbi-miR169c |
| Algae_064-2_Unigene_BMK.37703 | Dynein heavy chain 1, axonemal OS=Rattus norvegicus GN=Dnah1 PE=2 SV=2 | stu-miR171b-3p |
| Algae_064-2_Unigene_BMK.37724 | Uncharacterized proline-rich protein (Fragment) OS=Owenia fusiformis PE=4 SV=1 | sbi-miR169c |
| Algae_064-2_Unigene_BMK.3782 |  | stu-miR171b-3p |
| Algae_064-2_Unigene_BMK.37891 | Serine/arginine repetitive matrix protein 2 OS=Mus musculus GN=Srrm2 PE=1 SV=2 | osa-miR168a-5p |
| Algae_064-2_Unigene_BMK.37911 | Putative uncharacterized protein ENSP00000383309 OS=Homo sapiens PE=5 SV=3 | osa-miR168a-5p |
| Algae_064-2_Unigene_BMK.3792 | Uncharacterized protein C20orf26 OS=Homo sapiens GN=C20orf26 PE=2 SV=3 | aca-miR-5p-43924 |
| Algae_064-2_Unigene_BMK.37970 | Translation initiation factor IF-2 OS=Mycobacterium tuberculosis GN=infB PE=3 SV=1 | zma-miR529-5p |
| Algae_064-2_Unigene_BMK.38080 | RING-H2 finger protein ATL72 OS=Arabidopsis thaliana GN=ATL72 PE=2 SV=1 | rgl-miR5139_L+3 |
| Algae_064-2_Unigene_BMK.38330 | Serine/arginine repetitive matrix protein 1 OS=Gallus gallus GN=SRRM1 PE=2 SV=1 | sbi-miR169c |
| Algae_064-2_Unigene_BMK.38404 |  | aca-miR-3p-456915 |
| Algae_064-2_Unigene_BMK.38443 | Vegetative cell wall protein gp1 OS=Chlamydomonas reinhardtii GN=GP1 PE=2 SV=1 | aca-miR-5p-43924 |
| Algae_064-2_Unigene_BMK.38741 | Uncharacterized aminotransferase YfbQ OS=Shigella flexneri GN=yfbQ PE=3 SV=1 | aca-miR-5p-43924 |
| Algae_064-2_Unigene_BMK.38850 | Glycoprotein gp2 OS=Equine herpesvirus 1 (strain V592) GN=71 PE=3 SV=1 | stu-miR171b-3p |
| Algae_064-2_Unigene_BMK.38854 | Glucose-repressible alcohol dehydrogenase transcriptional effector OS=Candida glabrata GN=CCR4 PE=3 SV=1 | sbi-miR169c |
| Algae_064-2_Unigene_BMK.38864 | Translation initiation factor IF-2 OS=Synechococcus sp. (strain CC9311) GN=infB PE=3 SV=1 | bdi-miR7732-3p_L-1_1ss11GC |
| Algae_064-2_Unigene_BMK.38956 | Serine/arginine repetitive matrix protein 2 OS=Homo sapiens GN=SRRM2 PE=1 SV=2 | aca-miR-5p-43924 |
| Algae_064-2_Unigene_BMK.3903 | UPF0317 protein KRH_21160 OS=Kocuria rhizophila (strain ATCC 9341 / DSM 348 / NBRC 103217 / DC2201) GN=KRH_21160 PE=3 SV=1 | osa-miR168a-5p |
| Algae_064-2_Unigene_BMK.39086 | UPF0187 protein sll1024 OS=Synechocystis sp. (strain ATCC 27184 / PCC 6803 / N-1) GN=sll1024 PE=3 SV=2 | bdi-miR7732-3p_L-1_1ss11GC |
| Algae_064-2_Unigene_BMK.39161 | Metal resistance protein YCF1 OS=Saccharomyces cerevisiae GN=YCF1 PE=1 SV=2 | aca-miR-3p-456915 |
| Algae_064-2_Unigene_BMK.39269 | Uncharacterized protein yloA OS=Bacillus subtilis GN=yloA PE=2 SV=1 | sbi-miR169c |
| Algae_064-2_Unigene_BMK.39328 | Troponin I OS=Chlamys nipponensis akazara PE=1 SV=2 | osa-miR2876-3p_R+1 |
| Algae_064-2_Unigene_BMK.39588 | Translation initiation factor IF-2 OS=Frankia alni (strain ACN14a) GN=infB PE=3 SV=1 | aca-miR-5p-43924 |
| Algae_064-2_Unigene_BMK.3959 | Mucin-5AC (Fragments) OS=Homo sapiens GN=MUC5AC PE=1 SV=3 | aca-miR-5p-43924 |
| Algae_064-2_Unigene_BMK.39641 | Putative UDP-glucose 6-dehydrogenase ytcA OS=Bacillus subtilis GN=ytcA PE=3 SV=1 | aca-miR-5p-43924 |
| Algae_064-2_Unigene_BMK.39732 | Hypersensitive-induced response protein 3 OS=Arabidopsis thaliana GN=HIR3 PE=1 SV=1 | sbi-miR169c |
| Algae_064-2_Unigene_BMK.39850 |  | zma-miR529-5p |
| Algae_064-2_Unigene_BMK.39856 |  | tae-miR159a |
| Algae_064-2_Unigene_BMK.39930 | Uncharacterized protein LOC284861 OS=Homo sapiens PE=2 SV=1 | osa-miR168a-5p |
| Algae_064-2_Unigene_BMK.3998 | Sulfite reductase [NADPH] flavoprotein component OS=Saccharomyces cerevisiae GN=MET10 PE=1 SV=2 | aca-miR-5p-43924 |
| Algae_064-2_Unigene_BMK.40046 | Transcriptional repressor rco-1 OS=Neurospora crassa GN=rco-1 PE=4 SV=2 | aca-miR-5p-43924 |
| Algae_064-2_Unigene_BMK.40212 | Putative protein TPRXL OS=Homo sapiens GN=TPRXL PE=5 SV=2 | aca-miR-5p-43924 |
| Algae_064-2_Unigene_BMK.40698 | Protein NLRC3 OS=Homo sapiens GN=NLRC3 PE=2 SV=2 | aca-miR-3p-456915 |
| Algae_064-2_Unigene_BMK.40912 | Histidyl-tRNA synthetase OS=Leptospira borgpetersenii serovar Hardjo-bovis (strain L550) GN=hisS PE=3 SV=1 | sbi-miR169c |
| Algae_064-2_Unigene_BMK.41158 | Abhydrolase domain-containing protein FAM108B1 OS=Homo sapiens GN=FAM108B1 PE=2 SV=1 | aca-miR-5p-43924 |
| Algae_064-2_Unigene_BMK.4161 | Basic proline-rich protein OS=Sus scrofa PE=1 SV=2 | osa-miR168a-5p |
| Algae_064-2_Unigene_BMK.41653 |  | stu-miR171b-3p |
| Algae_064-2_Unigene_BMK.41911 |  | aca-miR-5p-43924 |
| Algae_064-2_Unigene_BMK.42077 |  | bdi-miR7732-3p_L-1_1ss11GC |
| Algae_064-2_Unigene_BMK.42275 |  | sbi-miR169c |
| Algae_064-2_Unigene_BMK.4233 | Developmentally-regulated GTP-binding protein 1 OS=Homo sapiens GN=DRG1 PE=1 SV=1 | tae-miR159a |
| Algae_064-2_Unigene_BMK.4234 | Protein transport protein SEC31 OS=Magnaporthe oryzae (strain 70-15 / FGSC 8958) GN=SEC31 PE=3 SV=1 | sbi-miR169c |
| Algae_064-2_Unigene_BMK.42639 |  | aca-miR-5p-43924 |
| Algae_064-2_Unigene_BMK.42712 |  | bdi-miR7732-3p_L-1_1ss11GC |
| Algae_064-2_Unigene_BMK.42741 | Serine/arginine repetitive matrix protein 1 OS=Mus musculus GN=Srrm1 PE=1 SV=1 | sbi-miR169c |
| Algae_064-2_Unigene_BMK.42931 | Lipase OS=Rhizomucor miehei PE=1 SV=2 | zma-miR529-5p |
| Algae_064-2_Unigene_BMK.43224 |  | stu-miR171b-3p |
| Algae_064-2_Unigene_BMK.43259 | Basic juvenile hormone-suppressible protein 2 OS=Trichoplusia ni GN=BJSP-2 PE=1 SV=1 | sbi-miR169c |
| Algae_064-2_Unigene_BMK.43294 | Formin-like protein 5 OS=Oryza sativa subsp. japonica GN=FH5 PE=2 SV=2 | stu-miR171b-3p |
| Algae_064-2_Unigene_BMK.43328 |  | zma-miR529-5p |
| Algae_064-2_Unigene_BMK.43395 | Peptidyl-prolyl cis-trans isomerase CYP19-3 OS=Arabidopsis thaliana GN=CYP19-3 PE=1 SV=2 | rgl-miR5139_L+3 |
| Algae_064-2_Unigene_BMK.43467 | Mitochondrial uncoupling protein 2 OS=Bos taurus GN=UCP2 PE=2 SV=1 | sbi-miR169c |
| Algae_064-2_Unigene_BMK.43697 |  | sbi-miR169c |
| Algae_064-2_Unigene_BMK.44053 | Pentatricopeptide repeat-containing protein At2g31400, chloroplastic OS=Arabidopsis thaliana GN=At2g31400 PE=2 SV=1 | bdi-miR7732-3p_L-1_1ss11GC |
| Algae_064-2_Unigene_BMK.44392 | Vegetative incompatibility protein HET-E-1 OS=Podospora anserina GN=HET-E1 PE=4 SV=1 | zma-miR529-5p |
| Algae_064-2_Unigene_BMK.44436 |  | aca-miR-5p-43924 |
| Algae_064-2_Unigene_BMK.44454 | Formin-like protein 5 OS=Oryza sativa subsp. japonica GN=FH5 PE=2 SV=2 | aca-miR-5p-43924 |
| Algae_064-2_Unigene_BMK.44480 |  | osa-miR168a-5p |
| Algae_064-2_Unigene_BMK.44751 | Egl nine homolog 1 OS=Rattus norvegicus GN=Egln1 PE=2 SV=1 | aca-miR-5p-43924 |
| Algae_064-2_Unigene_BMK.44810 |  | aca-miR-5p-43924 |
| Algae_064-2_Unigene_BMK.44867 | Kinesin-like protein KIF18B OS=Mus musculus GN=Kif18b PE=2 SV=2 | bdi-miR7732-3p_L-1_1ss11GC |
| Algae_064-2_Unigene_BMK.44945 | Peptide methionine sulfoxide reductase B5 OS=Oryza sativa subsp. japonica GN=MSRB5 PE=2 SV=1 | bdi-miR7732-3p_L-1_1ss11GC |
| Algae_064-2_Unigene_BMK.45058 | Flocculation protein FLO11 OS=Saccharomyces cerevisiae GN=MUC1 PE=1 SV=2 | aca-miR-3p-456915 |
| Algae_064-2_Unigene_BMK.45211 | Cholinesterase OS=Equus caballus GN=BCHE PE=1 SV=1 | osa-miR168a-5p |
| Algae_064-2_Unigene_BMK.45431 | Probable zinc transporter 12 OS=Arabidopsis thaliana GN=ZIP12 PE=2 SV=1 | osa-miR168a-5p |
| Algae_064-2_Unigene_BMK.46166 | Sucrose-phosphatase 2 OS=Oryza sativa subsp. japonica GN=SPP2 PE=1 SV=1 | osa-miR168a-5p |
| Algae_064-2_Unigene_BMK.46399 | Putative callose synthase 6 OS=Arabidopsis thaliana GN=CALS6 PE=3 SV=2 | sbi-miR169c |
| Algae_064-2_Unigene_BMK.4643 | Uncharacterized protein BHLF1 OS=Epstein-Barr virus (strain B95-8) GN=BHLF1 PE=4 SV=1 | aca-miR-5p-43924 |
| Algae_064-2_Unigene_BMK.46492 | Pentatricopeptide repeat-containing protein At2g41720 OS=Arabidopsis thaliana GN=EMB2654 PE=2 SV=1 | rgl-miR5139_L+3 |
| Algae_064-2_Unigene_BMK.46614 | Dynein heavy chain 10, axonemal OS=Homo sapiens GN=DNAH10 PE=1 SV=4 | stu-miR171b-3p |
| Algae_064-2_Unigene_BMK.46722 |  | osa-miR2876-3p_R+1 |
| Algae_064-2_Unigene_BMK.46771 |  | sbi-miR169c |
| Algae_064-2_Unigene_BMK.46912 | Flocculation protein FLO11 OS=Saccharomyces cerevisiae GN=MUC1 PE=1 SV=2 | aca-miR-5p-43924 |
| Algae_064-2_Unigene_BMK.46932 |  | stu-miR171b-3p |
| Algae_064-2_Unigene_BMK.46973 |  | sbi-miR169c |
| Algae_064-2_Unigene_BMK.47120 |  | zma-miR529-5p |
| Algae_064-2_Unigene_BMK.47363 | Serine/arginine repetitive matrix protein 1 OS=Mus musculus GN=Srrm1 PE=1 SV=1 | rgl-miR5139_L+3 |
| Algae_064-2_Unigene_BMK.47609 |  | aca-miR-5p-43924 |
| Algae_064-2_Unigene_BMK.47899 | Myeloid leukemia factor OS=Drosophila melanogaster GN=Mlf PE=1 SV=2 | aca-miR-5p-43924 |
| Algae_064-2_Unigene_BMK.47937 | Formin-like protein 5 OS=Oryza sativa subsp. japonica GN=FH5 PE=2 SV=2 | aca-miR-5p-43924 |
| Algae_064-2_Unigene_BMK.48012 | Serine/arginine repetitive matrix protein 1 OS=Gallus gallus GN=SRRM1 PE=2 SV=1 | osa-miR168a-5p |
| Algae_064-2_Unigene_BMK.48494 | Cytochrome c6 OS=Bumilleriopsis filiformis GN=petJ PE=1 SV=1 | tae-miR159a |
| Algae_064-2_Unigene_BMK.4870 | Putative exosome complex exonuclease rrp40 OS=Dictyostelium discoideum GN=exosc3 PE=3 SV=1 | bdi-miR7732-3p_L-1_1ss11GC |
| Algae_064-2_Unigene_BMK.49054 | Nickel-binding periplasmic protein OS=Escherichia coli (strain K12) GN=nikA PE=1 SV=1 | aca-miR-5p-43924 |
| Algae_064-2_Unigene_BMK.4916 | E3 ubiquitin-protein ligase RLIM OS=Mus musculus GN=Rlim PE=1 SV=2 | osa-miR2876-3p_R+1 |
| Algae_064-2_Unigene_BMK.49164 | Fumarylacetoacetase OS=Bos taurus GN=FAH PE=2 SV=1 | aca-miR-3p-456915 |
| Algae_064-2_Unigene_BMK.49208 | Ubiquitin carboxyl-terminal hydrolase 51 OS=Homo sapiens GN=USP51 PE=2 SV=1 | zma-miR529-5p |
| Algae_064-2_Unigene_BMK.49329 |  | sbi-miR169c |
| Algae_064-2_Unigene_BMK.49368 |  | osa-miR168a-5p |
| Algae_064-2_Unigene_BMK.49372 | ATP-dependent RNA helicase DHX8 OS=Homo sapiens GN=DHX8 PE=1 SV=1 | aca-miR-5p-43924 |
| Algae_064-2_Unigene_BMK.49443 | Wiskott-Aldrich syndrome protein homolog OS=Mus musculus GN=Was PE=1 SV=1 | aca-miR-3p-456915 |
| Algae_064-2_Unigene_BMK.49504 | Proline-rich proteoglycan 2 OS=Rattus norvegicus GN=Prpg2 PE=1 SV=2 | zma-miR529-5p |
| Algae_064-2_Unigene_BMK.49635 | Serine/arginine repetitive matrix protein 3 OS=Homo sapiens GN=SRRM3 PE=2 SV=4 | aca-miR-5p-43924 |
| Algae_064-2_Unigene_BMK.49669 | Protein DD3-3 OS=Dictyostelium discoideum GN=DD3-3 PE=2 SV=1 | osa-miR168a-5p |
| Algae_064-2_Unigene_BMK.4975 | Serine/arginine repetitive matrix protein 2 OS=Homo sapiens GN=SRRM2 PE=1 SV=2 | sbi-miR169c |
| Algae_064-2_Unigene_BMK.49803 | Uncharacterized ATP-dependent helicase yprA OS=Bacillus subtilis GN=yprA PE=3 SV=1 | osa-miR168a-5p |
| Algae_064-2_Unigene_BMK.49903 | Isopentenyl-diphosphate Delta-isomerase II OS=Arabidopsis thaliana GN=IPP2 PE=2 SV=1 | aca-miR-5p-43924 |
| Algae_064-2_Unigene_BMK.49919 |  | aca-miR-3p-456915 |
| Algae_064-2_Unigene_BMK.50142 | Kinesin light chain 2 OS=Homo sapiens GN=KLC2 PE=1 SV=1 | zma-miR529-5p |
| Algae_064-2_Unigene_BMK.50206 | ABC transporter F family member 2 OS=Dictyostelium discoideum GN=abcF2 PE=3 SV=1 | sbi-miR169c |
| Algae_064-2_Unigene_BMK.50302 |  | aca-miR-5p-43924 |
| Algae_064-2_Unigene_BMK.50317 | Serine/threonine-protein kinase SIK2 OS=Pongo abelii GN=SIK2 PE=2 SV=1 | osa-miR168a-5p |
| Algae_064-2_Unigene_BMK.50617 | Serine/arginine repetitive matrix protein 1 OS=Homo sapiens GN=SRRM1 PE=1 SV=2 | aca-miR-5p-43924 |
| Algae_064-2_Unigene_BMK.50715 |  | osa-miR168a-5p |
| Algae_064-2_Unigene_BMK.508 | Uncharacterized protein DDB_G0271670 OS=Dictyostelium discoideum GN=DDB_G0271670 PE=4 SV=1 | tae-miR159a |
| Algae_064-2_Unigene_BMK.50814 | Twitchin OS=Caenorhabditis elegans GN=unc-22 PE=1 SV=3 | aca-miR-5p-43924 |
| Algae_064-2_Unigene_BMK.50896 | Translation initiation factor IF-2 OS=Arthrobacter chlorophenolicus (strain A6 / ATCC 700700 / DSM 12829 / JCM 12360) GN=infB PE=3 SV=1 | osa-miR168a-5p |
| Algae_064-2_Unigene_BMK.51192 | Tankyrase-2 OS=Homo sapiens GN=TNKS2 PE=1 SV=1 | osa-miR168a-5p |
| Algae_064-2_Unigene_BMK.51651 | Translation initiation factor IF-2 OS=Frankia sp. (strain EAN1pec) GN=infB PE=3 SV=1 | stu-miR171b-3p |
| Algae_064-2_Unigene_BMK.51665 | Putative protein TPRXL OS=Homo sapiens GN=TPRXL PE=5 SV=2 | osa-miR168a-5p |
| Algae_064-2_Unigene_BMK.51858 | Translation initiation factor IF-2 OS=Mycobacterium sp. (strain MCS) GN=infB PE=3 SV=1 | sbi-miR169c |
| Algae_064-2_Unigene_BMK.51922 |  | bdi-miR7732-3p_L-1_1ss11GC |
| Algae_064-2_Unigene_BMK.51955 | Glutathione S-transferase 1 OS=Ascaris suum GN=GST1 PE=1 SV=3 | osa-miR168a-5p |
| Algae_064-2_Unigene_BMK.51989 | MFS-type transporter C6orf192 homolog OS=Mus musculus PE=2 SV=2 | aca-miR-3p-456915 |
| Algae_064-2_Unigene_BMK.521 |  | zma-miR529-5p |
| Algae_064-2_Unigene_BMK.52242 |  | osa-miR168a-5p |
| Algae_064-2_Unigene_BMK.5235 | Translation initiation factor IF-2 OS=Frankia sp. (strain EAN1pec) GN=infB PE=3 SV=1 | sbi-miR169c |
| Algae_064-2_Unigene_BMK.52412 | Gamma-butyrobetaine dioxygenase OS=Mus musculus GN=Bbox1 PE=2 SV=1 | aca-miR-3p-456915 |
| Algae_064-2_Unigene_BMK.52443 | Putative uncharacterized protein FLJ22184 OS=Homo sapiens PE=1 SV=1 | osa-miR168a-5p |
| Algae_064-2_Unigene_BMK.52574 |  | bdi-miR7732-3p_L-1_1ss11GC |
| Algae_064-2_Unigene_BMK.52604 |  | sbi-miR169c |
| Algae_064-2_Unigene_BMK.5267 | Uncharacterized protein C16orf48 homolog OS=Mus musculus PE=2 SV=1 | zma-miR529-5p |
| Algae_064-2_Unigene_BMK.52737 | Elongation factor G, chloroplastic OS=Glycine max GN=FUSA PE=3 SV=1 | osa-miR168a-5p |
| Algae_064-2_Unigene_BMK.5275 |  | sbi-miR169c |
| Algae_064-2_Unigene_BMK.52908 |  | aca-miR-5p-43924 |
| Algae_064-2_Unigene_BMK.53030 | Glycoprotein gp2 OS=Equine herpesvirus 1 (strain V592) GN=71 PE=3 SV=1 | sbi-miR169c |
| Algae_064-2_Unigene_BMK.53036 | Serine/arginine repetitive matrix protein 2 OS=Homo sapiens GN=SRRM2 PE=1 SV=2 | sbi-miR169c |
| Algae_064-2_Unigene_BMK.53087 |  | osa-miR168a-5p |
| Algae_064-2_Unigene_BMK.53133 |  | osa-miR168a-5p |
| Algae_064-2_Unigene_BMK.53185 | Peptidyl-prolyl cis-trans isomerase B OS=Haemophilus influenzae (strain ATCC 51907 / DSM 11121 / KW20 / Rd) GN=ppiB PE=3 SV=1 | zma-miR529-5p |
| Algae_064-2_Unigene_BMK.53252 | ADP-ribosyl-[dinitrogen reductase] glycohydrolase OS=Rhodospirillum rubrum GN=draG PE=1 SV=1 | aca-miR-5p-43924 |
| Algae_064-2_Unigene_BMK.53517 | Uncharacterized protein BHLF1 OS=Epstein-Barr virus (strain B95-8) GN=BHLF1 PE=4 SV=1 | sbi-miR169c |
| Algae_064-2_Unigene_BMK.5364 | Arylsulfatase B OS=Rattus norvegicus GN=Arsb PE=2 SV=2 | osa-miR168a-5p |
| Algae_064-2_Unigene_BMK.53670 | Viral protein TPX OS=Thermoproteus tenax virus 1 (strain VT3) PE=4 SV=1 | aca-miR-5p-43924 |
| Algae_064-2_Unigene_BMK.53700 | Kinesin-like protein KIF1C OS=Mus musculus GN=Kif1c PE=1 SV=2 | bdi-miR7732-3p_L-1_1ss11GC |
| Algae_064-2_Unigene_BMK.53943 |  | zma-miR529-5p |
| Algae_064-2_Unigene_BMK.54139 | N-acyl-D-aspartate deacylase OS=Alcaligenes xylosoxydans xylosoxydans PE=1 SV=1 | sbi-miR169c |
| Algae_064-2_Unigene_BMK.54406 | Spermatogenesis-associated protein 5 OS=Mus musculus GN=Spata5 PE=2 SV=2 | osa-miR168a-5p |
| Algae_064-2_Unigene_BMK.54442 | Magnesium transporter NIPA3 OS=Pongo abelii GN=NIPAL1 PE=2 SV=1 | stu-miR171b-3p |
| Algae_064-2_Unigene_BMK.54844 |  | aca-miR-5p-43924 |
| Algae_064-2_Unigene_BMK.55090 | Carbonyl reductase family member 4 OS=Rattus norvegicus GN=Cbr4 PE=2 SV=1 | tae-miR159a |
| Algae_064-2_Unigene_BMK.55374 | Keratin-associated protein 5-2 OS=Mus musculus GN=Krtap5-2 PE=2 SV=1 | sbi-miR169c |
| Algae_064-2_Unigene_BMK.55549 |  | sbi-miR169c |
| Algae_064-2_Unigene_BMK.55696 |  | stu-miR171b-3p |
| Algae_064-2_Unigene_BMK.557 | Taxadiene 5-alpha hydroxylase OS=Taxus cuspidata PE=1 SV=2 | rgl-miR5139_L+3 |
| Algae_064-2_Unigene_BMK.55768 | Anoctamin-10 OS=Mus musculus GN=Ano10 PE=2 SV=1 | bdi-miR7732-3p_L-1_1ss11GC |
| Algae_064-2_Unigene_BMK.5583 |  | sbi-miR169c |
| Algae_064-2_Unigene_BMK.5588 |  | aca-miR-5p-43924 |
| Algae_064-2_Unigene_BMK.5600 | Digalactosyldiacylglycerol synthase 1, chloroplastic OS=Arabidopsis thaliana GN=DGD1 PE=1 SV=1 | rgl-miR5139_L+3 |
| Algae_064-2_Unigene_BMK.5603 |  | sbi-miR169c |
| Algae_064-2_Unigene_BMK.56180 | mRNA 3'-end-processing protein yth1 OS=Emericella nidulans GN=yth1 PE=3 SV=1 | zma-miR529-5p |
| Algae_064-2_Unigene_BMK.56198 | Nuclear pore complex protein Nup98-Nup96 OS=Dictyostelium discoideum GN=nup98 PE=3 SV=1 | zma-miR529-5p |
| Algae_064-2_Unigene_BMK.56276 |  | aca-miR-5p-43924 |
| Algae_064-2_Unigene_BMK.56494 |  | tae-miR159a |
| Algae_064-2_Unigene_BMK.56551 |  | sbi-miR169c |
| Algae_064-2_Unigene_BMK.56576 | Phytochrome OS=Picea abies PE=2 SV=1 | stu-miR171b-3p |
| Algae_064-2_Unigene_BMK.56616 | Translation initiation factor IF-2 OS=Frankia alni (strain ACN14a) GN=infB PE=3 SV=1 | aca-miR-5p-43924 |
| Algae_064-2_Unigene_BMK.57281 |  | sbi-miR169c |
| Algae_064-2_Unigene_BMK.5772 | Leucoanthocyanidin dioxygenase OS=Zea mays GN=A2 PE=2 SV=1 | aca-miR-3p-456915 |
| Algae_064-2_Unigene_BMK.57878 | Pentatricopeptide repeat-containing protein At2g31400, chloroplastic OS=Arabidopsis thaliana GN=At2g31400 PE=2 SV=1 | sbi-miR169c |
| Algae_064-2_Unigene_BMK.5803 | Malate dehydrogenase, mitochondrial OS=Chlamydomonas reinhardtii PE=3 SV=1 | aca-miR-3p-456915 |
| Algae_064-2_Unigene_BMK.58079 |  | osa-miR168a-5p |
| Algae_064-2_Unigene_BMK.58232 |  | sbi-miR169c |
| Algae_064-2_Unigene_BMK.58301 | Pentatricopeptide repeat-containing protein At1g12775, mitochondrial OS=Arabidopsis thaliana GN=At1g12775 PE=2 SV=1 | zma-miR529-5p |
| Algae_064-2_Unigene_BMK.58304 | Thymidylate kinase OS=Schizosaccharomyces pombe GN=tmp1 PE=2 SV=2 | aca-miR-5p-43924 |
| Algae_064-2_Unigene_BMK.58535 | Ribulose bisphosphate carboxylase small chain 1B, chloroplastic OS=Arabidopsis thaliana GN=RBCS-1B PE=1 SV=1 | stu-miR171b-3p |
| Algae_064-2_Unigene_BMK.59020 | Vegetative cell wall protein gp1 OS=Chlamydomonas reinhardtii GN=GP1 PE=2 SV=1 | osa-miR168a-5p |
| Algae_064-2_Unigene_BMK.59160 | [Pyruvate dehydrogenase [lipoamide]] kinase isozyme 3, mitochondrial OS=Homo sapiens GN=PDK3 PE=1 SV=1 | zma-miR529-5p |
| Algae_064-2_Unigene_BMK.59502 | Regulator of nonsense transcripts 1 homolog OS=Drosophila melanogaster GN=Upf1 PE=1 SV=2 | stu-miR171b-3p |
| Algae_064-2_Unigene_BMK.59537 | Probable beta-D-xylosidase 2 OS=Arabidopsis thaliana GN=BXL2 PE=2 SV=1 | sbi-miR169c |
| Algae_064-2_Unigene_BMK.59574 |  | aca-miR-3p-456915 |
| Algae_064-2_Unigene_BMK.59772 |  | rgl-miR5139_L+3 |
| Algae_064-2_Unigene_BMK.60213 |  | sbi-miR169c |
| Algae_064-2_Unigene_BMK.6028 | Serine/arginine repetitive matrix protein 2 OS=Homo sapiens GN=SRRM2 PE=1 SV=2 | zma-miR529-5p |
| Algae_064-2_Unigene_BMK.60311 |  | osa-miR168a-5p |
| Algae_064-2_Unigene_BMK.60544 | Facilitator of iron transport 1 OS=Saccharomyces cerevisiae GN=FIT1 PE=2 SV=1 | sbi-miR169c |
| Algae_064-2_Unigene_BMK.6063 | TBC1 domain family member 4 OS=Homo sapiens GN=TBC1D4 PE=1 SV=2 | sbi-miR169c |
| Algae_064-2_Unigene_BMK.60672 | Translation initiation factor IF-2 OS=Synechococcus sp. (strain RCC307) GN=infB PE=3 SV=1 | zma-miR529-5p |
| Algae_064-2_Unigene_BMK.60769 | Mucin-5AC (Fragments) OS=Homo sapiens GN=MUC5AC PE=1 SV=3 | stu-miR171b-3p |
| Algae_064-2_Unigene_BMK.60938 |  | stu-miR171b-3p |
| Algae_064-2_Unigene_BMK.61062 | Translation initiation factor IF-2 OS=Nocardia farcinica GN=infB PE=3 SV=2 | osa-miR168a-5p |
| Algae_064-2_Unigene_BMK.61613 | LanC-like protein 2 OS=Mus musculus GN=Lancl2 PE=1 SV=1 | rgl-miR5139_L+3 |
| Algae_064-2_Unigene_BMK.617 | Uncharacterized aarF domain-containing protein kinase 1 OS=Homo sapiens GN=ADCK1 PE=1 SV=2 | aca-miR-3p-456915 |
| Algae_064-2_Unigene_BMK.6208 | Translation initiation factor IF-2 OS=Mycobacterium vanbaalenii (strain DSM 7251 / PYR-1) GN=infB PE=3 SV=1 | zma-miR529-5p |
| Algae_064-2_Unigene_BMK.6215 |  | sbi-miR169c |
| Algae_064-2_Unigene_BMK.62261 | Serine/arginine repetitive matrix protein 2 OS=Mus musculus GN=Srrm2 PE=1 SV=2 | aca-miR-5p-43924 |
| Algae_064-2_Unigene_BMK.62276 | JmjC domain-containing protein 8 OS=Homo sapiens GN=JMJD8 PE=2 SV=1 | osa-miR168a-5p |
| Algae_064-2_Unigene_BMK.62301 | Extensin OS=Nicotiana tabacum GN=HRGPNT3 PE=2 SV=1 | stu-miR171b-3p |
| Algae_064-2_Unigene_BMK.62600 |  | sbi-miR169c |
| Algae_064-2_Unigene_BMK.62630 | Translation initiation factor IF-2 OS=Rhodococcus opacus (strain B4) GN=infB PE=3 SV=1 | sbi-miR169c |
| Algae_064-2_Unigene_BMK.62680 |  | sbi-miR169c |
| Algae_064-2_Unigene_BMK.6270 | Gibberellin 20 oxidase 2 OS=Oryza sativa subsp. japonica GN=20ox2 PE=1 SV=1 | stu-miR171b-3p |
| Algae_064-2_Unigene_BMK.62894 | Serine-rich adhesin for platelets OS=Staphylococcus haemolyticus (strain JCSC1435) GN=sraP PE=3 SV=1 | bdi-miR7732-3p_L-1_1ss11GC |
| Algae_064-2_Unigene_BMK.63166 | Uncharacterized protein DDB_G0271670 OS=Dictyostelium discoideum GN=DDB_G0271670 PE=4 SV=1 | sbi-miR169c |
| Algae_064-2_Unigene_BMK.63167 | Flocculation protein FLO11 OS=Saccharomyces cerevisiae GN=MUC1 PE=1 SV=2 | sbi-miR169c |
| Algae_064-2_Unigene_BMK.63498 | PPPDE peptidase domain-containing protein 2 OS=Homo sapiens GN=PPPDE2 PE=1 SV=1 | stu-miR171b-3p |
| Algae_064-2_Unigene_BMK.63516 | Translation initiation factor IF-2 OS=Frankia sp. (strain EAN1pec) GN=infB PE=3 SV=1 | aca-miR-5p-43924 |
| Algae_064-2_Unigene_BMK.6356 |  | aca-miR-3p-456915 |
| Algae_064-2_Unigene_BMK.63631 |  | rgl-miR5139_L+3 |
| Algae_064-2_Unigene_BMK.63773 |  | rgl-miR5139_L+3 |
| Algae_064-2_Unigene_BMK.64126 | Uncharacterized protein ORF4 OS=Grapevine fleck virus (isolate Italy/MT48) GN=ORF4 PE=4 SV=1 | aca-miR-5p-43924 |
| Algae_064-2_Unigene_BMK.64316 | UPF0317 protein KRH_21160 OS=Kocuria rhizophila (strain ATCC 9341 / DSM 348 / NBRC 103217 / DC2201) GN=KRH_21160 PE=3 SV=1 | aca-miR-5p-43924 |
| Algae_064-2_Unigene_BMK.644 | Mucin-19 OS=Mus musculus GN=Muc19 PE=2 SV=2 | bdi-miR7732-3p_L-1_1ss11GC |
| Algae_064-2_Unigene_BMK.64452 |  | osa-miR168a-5p |
| Algae_064-2_Unigene_BMK.6447 | Mps one binder kinase activator-like 1B OS=Rattus norvegicus GN=Mobkl1b PE=1 SV=3 | aca-miR-5p-43924 |
| Algae_064-2_Unigene_BMK.64526 |  | aca-miR-3p-456915 |
| Algae_064-2_Unigene_BMK.6466 | Glycoprotein gp2 OS=Equine herpesvirus 1 (strain V592) GN=71 PE=3 SV=1 | sbi-miR169c |
| Algae_064-2_Unigene_BMK.6467 | Serine/arginine repetitive matrix protein 2 OS=Mus musculus GN=Srrm2 PE=1 SV=2 | osa-miR168a-5p |
| Algae_064-2_Unigene_BMK.64834 | Bacitracin synthase 3 OS=Bacillus licheniformis GN=bacC PE=3 SV=1 | bdi-miR7732-3p_L-1_1ss11GC |
| Algae_064-2_Unigene_BMK.64850 | Ras-related protein YPTC6 OS=Chlamydomonas reinhardtii GN=YPTC6 PE=3 SV=1 | aca-miR-5p-43924 |
| Algae_064-2_Unigene_BMK.65050 | Serine/arginine repetitive matrix protein 2 OS=Homo sapiens GN=SRRM2 PE=1 SV=2 | zma-miR529-5p |
| Algae_064-2_Unigene_BMK.65565 | Pentatricopeptide repeat-containing protein At5g02860 OS=Arabidopsis thaliana GN=At5g02860 PE=2 SV=1 | aca-miR-5p-43924 |
| Algae_064-2_Unigene_BMK.65648 | ATP-dependent DNA helicase recQ OS=Haemophilus influenzae (strain ATCC 51907 / DSM 11121 / KW20 / Rd) GN=recQ PE=3 SV=1 | sbi-miR169c |
| Algae_064-2_Unigene_BMK.65746 | Pentatricopeptide repeat-containing protein At2g31400, chloroplastic OS=Arabidopsis thaliana GN=At2g31400 PE=2 SV=1 | bdi-miR7732-3p_L-1_1ss11GC |
| Algae_064-2_Unigene_BMK.65763 | Ubiquitin-conjugating enzyme E2 2 OS=Caenorhabditis elegans GN=let-70 PE=1 SV=1 | sbi-miR169c |
| Algae_064-2_Unigene_BMK.65804 |  | aca-miR-5p-43924 |
| Algae_064-2_Unigene_BMK.65806 | Rhomboid-related protein 3 OS=Homo sapiens GN=RHBDL3 PE=2 SV=1 | bdi-miR7732-3p_L-1_1ss11GC |
| Algae_064-2_Unigene_BMK.6589 | Ice-structuring glycoprotein (Fragment) OS=Notothenia coriiceps neglecta GN=afgp8 PE=1 SV=2 | sbi-miR169c |
| Algae_064-2_Unigene_BMK.65917 | Inner membrane protein alx OS=Salmonella typhi GN=alx PE=3 SV=1 | bdi-miR7732-3p_L-1_1ss11GC |
| Algae_064-2_Unigene_BMK.65932 | Probable E3 ubiquitin-protein ligase HECTD2 OS=Pongo abelii GN=HECTD2 PE=2 SV=1 | aca-miR-5p-43924 |
| Algae_064-2_Unigene_BMK.66027 | Putative protein TPRXL OS=Homo sapiens GN=TPRXL PE=5 SV=2 | aca-miR-5p-43924 |
| Algae_064-2_Unigene_BMK.66157 | 5' exonuclease Apollo OS=Ailuropoda melanoleuca GN=DCLRE1B PE=3 SV=1 | aca-miR-5p-43924 |
| Algae_064-2_Unigene_BMK.66302 | Translation initiation factor IF-2 OS=Rhodococcus sp. (strain RHA1) GN=infB PE=3 SV=1 | aca-miR-5p-43924 |
| Algae_064-2_Unigene_BMK.66431 | Small RNA 2'-O-methyltransferase OS=Xenopus tropicalis GN=henmt1 PE=2 SV=1 | osa-miR168a-5p |
| Algae_064-2_Unigene_BMK.66555 | Putative surface protein bspA-like OS=Trichomonas vaginalis GN=BSPAL1 PE=4 SV=1 | aca-miR-5p-43924 |
| Algae_064-2_Unigene_BMK.6659 | High affinity cGMP-specific 3',5'-cyclic phosphodiesterase 9A OS=Mus musculus GN=Pde9a PE=2 SV=1 | osa-miR168a-5p |
| Algae_064-2_Unigene_BMK.666 | Protein CREG1 OS=Gallus gallus GN=CREG1 PE=2 SV=1 | rgl-miR5139_L+3 |
| Algae_064-2_Unigene_BMK.66649 |  | sbi-miR169c |
| Algae_064-2_Unigene_BMK.66884 | Uncharacterized protein BHLF1 OS=Epstein-Barr virus (strain B95-8) GN=BHLF1 PE=4 SV=1 | rgl-miR5139_L+3 |
| Algae_064-2_Unigene_BMK.67010 |  | tae-miR159a |
| Algae_064-2_Unigene_BMK.67225 | Protein TBRG4 OS=Homo sapiens GN=TBRG4 PE=1 SV=1 | stu-miR171b-3p |
| Algae_064-2_Unigene_BMK.67305 | Basic proline-rich protein OS=Sus scrofa PE=1 SV=2 | stu-miR171b-3p |
| Algae_064-2_Unigene_BMK.67500 | Translation initiation factor IF-2 OS=Rhodococcus opacus (strain B4) GN=infB PE=3 SV=1 | aca-miR-5p-43924 |
| Algae_064-2_Unigene_BMK.67669 | Spondin-1 OS=Gallus gallus GN=SPON1 PE=2 SV=1 | zma-miR529-5p |
| Algae_064-2_Unigene_BMK.67753 |  | rgl-miR5139_L+3 |
| Algae_064-2_Unigene_BMK.67765 | Ubiquitin OS=Oryctolagus cuniculus PE=1 SV=1 | aca-miR-5p-43924 |
| Algae_064-2_Unigene_BMK.67796 | Cell wall protein TIR4 OS=Saccharomyces cerevisiae GN=TIR4 PE=1 SV=1 | zma-miR529-5p |
| Algae_064-2_Unigene_BMK.67840 |  | osa-miR168a-5p |
| Algae_064-2_Unigene_BMK.67957 |  | sbi-miR169c |
| Algae_064-2_Unigene_BMK.68040 | Serine-rich adhesin for platelets OS=Staphylococcus haemolyticus (strain JCSC1435) GN=sraP PE=3 SV=1 | osa-miR168a-5p |
| Algae_064-2_Unigene_BMK.6817 |  | aca-miR-5p-43924 |
| Algae_064-2_Unigene_BMK.68176 | Serine/arginine repetitive matrix protein 2 OS=Homo sapiens GN=SRRM2 PE=1 SV=2 | sbi-miR169c |
| Algae_064-2_Unigene_BMK.68233 |  | zma-miR529-5p |
| Algae_064-2_Unigene_BMK.68234 | Ribosomal protein L11 methyltransferase OS=Thermotoga neapolitana GN=prmA PE=3 SV=2 | bdi-miR7732-3p_L-1_1ss11GC |
| Algae_064-2_Unigene_BMK.68778 | Beta-glucosidase A OS=Clostridium thermocellum (strain ATCC 27405 / DSM 1237) GN=bglA PE=3 SV=1 | sbi-miR169c |
| Algae_064-2_Unigene_BMK.68789 | Serine/arginine repetitive matrix protein 3 OS=Homo sapiens GN=SRRM3 PE=2 SV=4 | aca-miR-5p-43924 |
| Algae_064-2_Unigene_BMK.68968 | Metal resistance protein YCF1 OS=Saccharomyces cerevisiae GN=YCF1 PE=1 SV=2 | aca-miR-5p-43924 |
| Algae_064-2_Unigene_BMK.69003 | Atherin OS=Homo sapiens GN=SAMD1 PE=1 SV=1 | osa-miR168a-5p |
| Algae_064-2_Unigene_BMK.69015 |  | bdi-miR7732-3p_L-1_1ss11GC |
| Algae_064-2_Unigene_BMK.69200 | Phosphoserine phosphatase OS=Archaeoglobus fulgidus GN=AF_2138 PE=3 SV=1 | stu-miR171b-3p |
| Algae_064-2_Unigene_BMK.69298 | Sterol 3-beta-glucosyltransferase OS=Pichia angusta GN=ATG26 PE=3 SV=1 | aca-miR-5p-43924 |
| Algae_064-2_Unigene_BMK.69332 | Probable E3 ubiquitin-protein ligase HERC1 OS=Homo sapiens GN=HERC1 PE=1 SV=2 | rgl-miR5139_L+3 |
| Algae_064-2_Unigene_BMK.69364 | Stress response protein nst1 OS=Sclerotinia sclerotiorum (strain ATCC 18683 / 1980 / Ss-1) GN=nst1 PE=3 SV=1 | osa-miR168a-5p |
| Algae_064-2_Unigene_BMK.69399 |  | tae-miR159a |
| Algae_064-2_Unigene_BMK.69611 | Serine/arginine repetitive matrix protein 2 OS=Homo sapiens GN=SRRM2 PE=1 SV=2 | stu-miR171b-3p |
| Algae_064-2_Unigene_BMK.69981 | Myosin-If OS=Mus musculus GN=Myo1f PE=1 SV=1 | aca-miR-5p-43924 |
| Algae_064-2_Unigene_BMK.70085 |  | rgl-miR5139_L+3 |
| Algae_064-2_Unigene_BMK.70156 | Pentatricopeptide repeat-containing protein At2g31400, chloroplastic OS=Arabidopsis thaliana GN=At2g31400 PE=2 SV=1 | stu-miR171b-3p |
| Algae_064-2_Unigene_BMK.70306 | Serine/arginine repetitive matrix protein 3 OS=Homo sapiens GN=SRRM3 PE=2 SV=4 | aca-miR-5p-43924 |
| Algae_064-2_Unigene_BMK.70396 | Translation initiation factor IF-2 OS=Frankia sp. (strain CcI3) GN=infB PE=3 SV=1 | aca-miR-5p-43924 |
| Algae_064-2_Unigene_BMK.7101 | 2-methylcitrate dehydratase 1 OS=Corynebacterium glutamicum GN=prpD1 PE=3 SV=1 | stu-miR171b-3p |
| Algae_064-2_Unigene_BMK.71367 |  | sbi-miR169c |
| Algae_064-2_Unigene_BMK.7138 | Kinesin-like protein K39 (Fragment) OS=Leishmania chagasi GN=KIN PE=2 SV=1 | bdi-miR7732-3p_L-1_1ss11GC |
| Algae_064-2_Unigene_BMK.71458 |  | stu-miR171b-3p |
| Algae_064-2_Unigene_BMK.71599 | Glycoprotein gp2 OS=Equine herpesvirus 1 (strain V592) GN=71 PE=3 SV=1 | sbi-miR169c |
| Algae_064-2_Unigene_BMK.71788 | D-aminoacylase OS=Alcaligenes xylosoxydans xylosoxydans GN=dan PE=1 SV=3 | stu-miR171b-3p |
| Algae_064-2_Unigene_BMK.71875 | Calcium/calmodulin-dependent protein kinase type 1B OS=Rattus norvegicus GN=Pnck PE=2 SV=1 | zma-miR529-5p |
| Algae_064-2_Unigene_BMK.71936 | ABC transporter C family member 3 OS=Dictyostelium discoideum GN=abcC3 PE=3 SV=1 | rgl-miR5139_L+3 |
| Algae_064-2_Unigene_BMK.72101 | Uncharacterized protein LOC284861 OS=Homo sapiens PE=2 SV=1 | osa-miR168a-5p |
| Algae_064-2_Unigene_BMK.72176 | Uncharacterized protein Rv1367c/MT1414 OS=Mycobacterium tuberculosis GN=Rv1367c PE=4 SV=2 | aca-miR-5p-43924 |
| Algae_064-2_Unigene_BMK.72236 | Serine/arginine repetitive matrix protein 2 OS=Mus musculus GN=Srrm2 PE=1 SV=2 | sbi-miR169c |
| Algae_064-2_Unigene_BMK.72237 | Cathepsin L OS=Sarcophaga peregrina PE=1 SV=1 | sbi-miR169c |
| Algae_064-2_Unigene_BMK.724 | Vegetative cell wall protein gp1 OS=Chlamydomonas reinhardtii GN=GP1 PE=2 SV=1 | sbi-miR169c |
| Algae_064-2_Unigene_BMK.725 | Serine/arginine repetitive matrix protein 2 OS=Homo sapiens GN=SRRM2 PE=1 SV=2 | zma-miR529-5p |
| Algae_064-2_Unigene_BMK.7259 | Transmembrane protein 110 OS=Rattus norvegicus GN=Tmem110 PE=2 SV=1 | osa-miR168a-5p |
| Algae_064-2_Unigene_BMK.72604 | Serine/threonine-protein phosphatase PP2A OS=Drosophila melanogaster GN=mts PE=1 SV=1 | zma-miR529-5p |
| Algae_064-2_Unigene_BMK.72832 | Solute carrier family 15 member 2 OS=Rattus norvegicus GN=Slc15a2 PE=2 SV=1 | aca-miR-3p-456915 |
| Algae_064-2_Unigene_BMK.72913 |  | sbi-miR169c |
| Algae_064-2_Unigene_BMK.73184 | Dynein heavy chain, cytoplasmic OS=Saccharomyces cerevisiae GN=DYN1 PE=1 SV=1 | zma-miR529-5p |
| Algae_064-2_Unigene_BMK.73460 | Sodium/hydrogen exchanger 8 OS=Rattus norvegicus GN=Slc9a8 PE=2 SV=1 | zma-miR529-5p |
| Algae_064-2_Unigene_BMK.73671 |  | tae-miR159a |
| Algae_064-2_Unigene_BMK.7390 | Gramicidin S biosynthesis protein GrsT OS=Aneurinibacillus migulanus GN=grsT PE=3 SV=1 | aca-miR-3p-456915 |
| Algae_064-2_Unigene_BMK.74055 | Transmembrane anterior posterior transformation protein 1 homolog OS=Danio rerio GN=tapt1 PE=3 SV=1 | osa-miR168a-5p |
| Algae_064-2_Unigene_BMK.7412 | Dehydrogenase/reductase SDR family member 1 OS=Mus musculus GN=Dhrs1 PE=2 SV=1 | aca-miR-5p-43924 |
| Algae_064-2_Unigene_BMK.74184 |  | osa-miR168a-5p |
| Algae_064-2_Unigene_BMK.74195 | Serine/arginine repetitive matrix protein 2 OS=Homo sapiens GN=SRRM2 PE=1 SV=2 | stu-miR171b-3p |
| Algae_064-2_Unigene_BMK.74336 |  | aca-miR-3p-456915 |
| Algae_064-2_Unigene_BMK.74360 | Zinc finger protein ZFPM1 OS=Homo sapiens GN=ZFPM1 PE=1 SV=2 | aca-miR-5p-43924 |
| Algae_064-2_Unigene_BMK.74413 |  | tae-miR159a |
| Algae_064-2_Unigene_BMK.74814 |  | sbi-miR169c |
| Algae_064-2_Unigene_BMK.74920 | Uncharacterized oxidoreductase YtbE OS=Bacillus subtilis GN=ytbE PE=1 SV=1 | bdi-miR7732-3p_L-1_1ss11GC |
| Algae_064-2_Unigene_BMK.7498 |  | bdi-miR7732-3p_L-1_1ss11GC |
| Algae_064-2_Unigene_BMK.75010 | Translation initiation factor IF-2 OS=Arthrobacter sp. (strain FB24) GN=infB PE=3 SV=1 | zma-miR529-5p |
| Algae_064-2_Unigene_BMK.7503 | Intraflagellar transport protein 81 homolog OS=Homo sapiens GN=IFT81 PE=1 SV=1 | aca-miR-3p-456915 |
| Algae_064-2_Unigene_BMK.75163 | Uncharacterized protein LOC284861 OS=Homo sapiens PE=2 SV=1 | osa-miR168a-5p |
| Algae_064-2_Unigene_BMK.75214 |  | rgl-miR5139_L+3 |
| Algae_064-2_Unigene_BMK.75310 | Protein mosB OS=Rhizobium meliloti GN=mosB PE=3 SV=1 | bdi-miR7732-3p_L-1_1ss11GC |
| Algae_064-2_Unigene_BMK.7536 | Cathepsin C OS=Schistosoma mansoni PE=2 SV=1 | osa-miR168a-5p |
| Algae_064-2_Unigene_BMK.75534 | Protein FAM110A OS=Homo sapiens GN=FAM110A PE=1 SV=1 | stu-miR169a-5p_R+1 |
| Algae_064-2_Unigene_BMK.75565 |  | sbi-miR169c |
| Algae_064-2_Unigene_BMK.7558 | G patch domain-containing protein 4 OS=Mus musculus GN=Gpatch4 PE=1 SV=1 | aca-miR-3p-456915 |
| Algae_064-2_Unigene_BMK.7562 |  | zma-miR529-5p |
| Algae_064-2_Unigene_BMK.75805 | Uncharacterized protein DKFZp434B061 OS=Homo sapiens PE=2 SV=2 | sbi-miR169c |
| Algae_064-2_Unigene_BMK.76032 | Keratin-associated protein 5-3 OS=Homo sapiens GN=KRTAP5-3 PE=2 SV=1 | osa-miR2876-3p_R+1 |
| Algae_064-2_Unigene_BMK.76033 | tRNA pseudouridine synthase C OS=Escherichia coli O6 GN=truC PE=3 SV=1 | sbi-miR169c |
| Algae_064-2_Unigene_BMK.76112 | Membrane-associated guanylate kinase, WW and PDZ domain-containing protein 2 OS=Homo sapiens GN=MAGI2 PE=1 SV=3 | bdi-miR7732-3p_L-1_1ss11GC |
| Algae_064-2_Unigene_BMK.76783 | WAS/WASL-interacting protein family member 1 OS=Rattus norvegicus GN=Wipf1 PE=1 SV=2 | aca-miR-5p-43924 |
| Algae_064-2_Unigene_BMK.76802 | Serine/arginine repetitive matrix protein 2 OS=Mus musculus GN=Srrm2 PE=1 SV=2 | stu-miR171b-3p |
| Algae_064-2_Unigene_BMK.7687 | Uncharacterized sodium-dependent transporter yocS OS=Bacillus subtilis GN=yocS PE=3 SV=1 | stu-miR171b-3p |
| Algae_064-2_Unigene_BMK.7712 | Malonyl-CoA-acyl carrier protein transacylase, mitochondrial OS=Homo sapiens GN=MCAT PE=1 SV=2 | sbi-miR169c |
| Algae_064-2_Unigene_BMK.77215 | Translation initiation factor IF-2 OS=Streptomyces griseus subsp. griseus (strain JCM 4626 / NBRC 13350) GN=infB PE=3 SV=1 | aca-miR-5p-43924 |
| Algae_064-2_Unigene_BMK.7736 |  | sbi-miR169c |
| Algae_064-2_Unigene_BMK.77413 | Formin-like protein 5 OS=Oryza sativa subsp. japonica GN=FH5 PE=2 SV=2 | stu-miR171b-3p |
| Algae_064-2_Unigene_BMK.77584 |  | rgl-miR5139_L+3 |
| Algae_064-2_Unigene_BMK.77606 |  | aca-miR-5p-43924 |
| Algae_064-2_Unigene_BMK.77886 |  | bdi-miR7732-3p_L-1_1ss11GC |
| Algae_064-2_Unigene_BMK.77908 |  | osa-miR168a-5p |
| Algae_064-2_Unigene_BMK.7799 | Peroxisomal membrane protein PMP34 OS=Mus musculus GN=Slc25a17 PE=2 SV=1 | osa-miR2876-3p_R+1 |
| Algae_064-2_Unigene_BMK.77995 | Sterol 3-beta-glucosyltransferase OS=Cryptococcus neoformans GN=ATG26 PE=3 SV=2 | aca-miR-3p-456915 |
| Algae_064-2_Unigene_BMK.7810 | Epstein-Barr nuclear antigen 1 OS=Epstein-Barr virus (strain GD1) GN=EBNA1 PE=1 SV=1 | sbi-miR169c |
| Algae_064-2_Unigene_BMK.78155 |  | aca-miR-5p-43924 |
| Algae_064-2_Unigene_BMK.78280 |  | osa-miR168a-5p |
| Algae_064-2_Unigene_BMK.78879 | Translation initiation factor IF-2 OS=Rhodococcus opacus (strain B4) GN=infB PE=3 SV=1 | stu-miR171b-3p |
| Algae_064-2_Unigene_BMK.79161 |  | aca-miR-3p-456915 |
| Algae_064-2_Unigene_BMK.79982 |  | sbi-miR169c |
| Algae_064-2_Unigene_BMK.80481 |  | tae-miR159a |
| Algae_064-2_Unigene_BMK.80767 | Mucin-5AC (Fragments) OS=Homo sapiens GN=MUC5AC PE=1 SV=3 | aca-miR-3p-456915 |
| Algae_064-2_Unigene_BMK.80806 |  | bdi-miR7732-3p_L-1_1ss11GC |
| Algae_064-2_Unigene_BMK.80851 | Ubiquitin OS=Coprinus congregatus PE=1 SV=1 | stu-miR171b-3p |
| Algae_064-2_Unigene_BMK.80919 | DBF4-type zinc finger-containing protein 2 homolog OS=Mus musculus GN=Zdbf2 PE=2 SV=1 | aca-miR-5p-43924 |
| Algae_064-2_Unigene_BMK.81274 | Cytokinesis protein sepH OS=Aspergillus niger (strain CBS 513.88 / FGSC A1513) GN=sepH PE=3 SV=1 | stu-miR171b-3p |
| Algae_064-2_Unigene_BMK.81776 |  | aca-miR-3p-456915 |
| Algae_064-2_Unigene_BMK.81966 | Cell wall protein DAN4 OS=Saccharomyces cerevisiae GN=DAN4 PE=2 SV=1 | rgl-miR5139_L+3 |
| Algae_064-2_Unigene_BMK.82028 |  | stu-miR171b-3p |
| Algae_064-2_Unigene_BMK.82107 |  | osa-miR168a-5p |
| Algae_064-2_Unigene_BMK.82269 | Calpain-D OS=Drosophila melanogaster GN=sol PE=1 SV=2 | stu-miR171b-3p |
| Algae_064-2_Unigene_BMK.82302 | Pentatricopeptide repeat-containing protein At1g63080, mitochondrial OS=Arabidopsis thaliana GN=At1g63080 PE=2 SV=1 | aca-miR-3p-456915 |
| Algae_064-2_Unigene_BMK.82324 |  | tae-miR159a |
| Algae_064-2_Unigene_BMK.82497 |  | stu-miR171b-3p |
| Algae_064-2_Unigene_BMK.8256 | Serine/arginine repetitive matrix protein 1 OS=Gallus gallus GN=SRRM1 PE=2 SV=1 | osa-miR168a-5p |
| Algae_064-2_Unigene_BMK.82642 | Pre-mRNA-splicing ATP-dependent RNA helicase prp28 OS=Neosartorya fischeri (strain ATCC 1020 / DSM 3700 / FGSC A1164 / NRRL 181) GN=prp28 PE=3 SV=1 | aca-miR-5p-43924 |
| Algae_064-2_Unigene_BMK.82781 | Potassium/sodium hyperpolarization-activated cyclic nucleotide-gated channel 4 OS=Rattus norvegicus GN=Hcn4 PE=2 SV=1 | osa-miR168a-5p |
| Algae_064-2_Unigene_BMK.82942 | 2-succinyl-5-enolpyruvyl-6-hydroxy-3-cyclohexene-1- carboxylate synthase OS=Rubrobacter xylanophilus (strain DSM 9941 / NBRC 16129) GN=menD PE=3 SV=1 | zma-miR529-5p |
| Algae_064-2_Unigene_BMK.8295 | Diacylglycerol O-acyltransferase 2B OS=Umbelopsis ramanniana GN=DGAT2B PE=1 SV=1 | sbi-miR169c |
| Algae_064-2_Unigene_BMK.83034 | Zinc finger CCCH domain-containing protein 56 OS=Oryza sativa subsp. japonica GN=Os08g0159800 PE=2 SV=1 | aca-miR-5p-43924 |
| Algae_064-2_Unigene_BMK.83211 |  | zma-miR529-5p |
| Algae_064-2_Unigene_BMK.83247 | STE20-like serine/threonine-protein kinase OS=Homo sapiens GN=SLK PE=1 SV=1 | aca-miR-5p-43924 |
| Algae_064-2_Unigene_BMK.83442 | Uncharacterized peptidase y4nA OS=Rhizobium sp. (strain NGR234) GN=NGR_a02410 PE=3 SV=1 | sbi-miR169c |
| Algae_064-2_Unigene_BMK.8348 | COBW domain-containing protein 1 OS=Rattus norvegicus GN=Cbwd1 PE=2 SV=2 | sbi-miR169c |
| Algae_064-2_Unigene_BMK.84074 | Histone-lysine N-methyltransferase ASHR1 OS=Arabidopsis thaliana GN=ASHR1 PE=2 SV=2 | stu-miR171b-3p |
| Algae_064-2_Unigene_BMK.84745 |  | aca-miR-3p-456915 |
| Algae_064-2_Unigene_BMK.84826 | Atherin OS=Oryctolagus cuniculus GN=SAMD1 PE=2 SV=1 | stu-miR171b-3p |
| Algae_064-2_Unigene_BMK.8489 | Nucleolin OS=Xenopus laevis GN=ncl PE=2 SV=3 | aca-miR-5p-43924 |
| Algae_064-2_Unigene_BMK.8493 | Eukaryotic initiation factor iso-4F subunit p82-34 OS=Triticum aestivum PE=1 SV=2 | aca-miR-5p-43924 |
| Algae_064-2_Unigene_BMK.84949 |  | bdi-miR7732-3p_L-1_1ss11GC |
| Algae_064-2_Unigene_BMK.85105 |  | osa-miR168a-5p |
| Algae_064-2_Unigene_BMK.85158 | Guanine nucleotide-binding protein-like 1 OS=Pongo abelii GN=GNL1 PE=2 SV=1 | stu-miR171b-3p |
| Algae_064-2_Unigene_BMK.85396 |  | zma-miR529-5p |
| Algae_064-2_Unigene_BMK.86159 | Hybrid signal transduction histidine kinase D OS=Dictyostelium discoideum GN=dhkD PE=2 SV=1 | osa-miR168a-5p |
| Algae_064-2_Unigene_BMK.86915 | Eukaryotic translation initiation factor 5A-4 OS=Solanum tuberosum GN=EIF5A4 PE=2 SV=1 | aca-miR-3p-456915 |
| Algae_064-2_Unigene_BMK.8714 | 4-hydroxy-2-oxovalerate aldolase OS=Rhodococcus sp. (strain RHA1) GN=bphF PE=2 SV=1 | aca-miR-3p-456915 |
| Algae_064-2_Unigene_BMK.8726 |  | aca-miR-3p-456915 |
| Algae_064-2_Unigene_BMK.87306 | Transmembrane protein 20 OS=Homo sapiens GN=TMEM20 PE=2 SV=1 | sbi-miR169c |
| Algae_064-2_Unigene_BMK.87440 | Pentatricopeptide repeat-containing protein At2g41720 OS=Arabidopsis thaliana GN=EMB2654 PE=2 SV=1 | tae-miR159a |
| Algae_064-2_Unigene_BMK.87482 |  | sbi-miR169c |
| Algae_064-2_Unigene_BMK.87562 | ABC transporter A family member 2 OS=Dictyostelium discoideum GN=abcA2 PE=3 SV=1 | aca-miR-5p-43924 |
| Algae_064-2_Unigene_BMK.87760 | Fumarate hydratase class I, anaerobic OS=Escherichia coli (strain K12) GN=fumB PE=1 SV=2 | rgl-miR5139_L+3 |
| Algae_064-2_Unigene_BMK.87939 | Serine/arginine repetitive matrix protein 3 OS=Homo sapiens GN=SRRM3 PE=2 SV=4 | aca-miR-5p-43924 |
| Algae_064-2_Unigene_BMK.88530 |  | rgl-miR5139_L+3 |
| Algae_064-2_Unigene_BMK.88638 |  | osa-miR168a-5p |
| Algae_064-2_Unigene_BMK.8875 |  | aca-miR-3p-456915 |
| Algae_064-2_Unigene_BMK.88787 | Collectin-12 OS=Mus musculus GN=Colec12 PE=1 SV=1 | stu-miR171b-3p |
| Algae_064-2_Unigene_BMK.889 | Uncharacterized protein BHLF1 OS=Epstein-Barr virus (strain B95-8) GN=BHLF1 PE=4 SV=1 | zma-miR529-5p |
| Algae_064-2_Unigene_BMK.8935 |  | aca-miR-3p-456915 |
| Algae_064-2_Unigene_BMK.8941 |  | aca-miR-3p-456915 |
| Algae_064-2_Unigene_BMK.89608 | Spore coat protein SP96 OS=Dictyostelium discoideum GN=cotA PE=4 SV=2 | osa-miR168a-5p |
| Algae_064-2_Unigene_BMK.8961 | Epstein-Barr nuclear antigen 1 OS=Epstein-Barr virus (strain GD1) GN=EBNA1 PE=1 SV=1 | aca-miR-5p-43924 |
| Algae_064-2_Unigene_BMK.89773 | Potassium voltage-gated channel subfamily H member 5 OS=Rattus norvegicus GN=Kcnh5 PE=2 SV=1 | zma-miR529-5p |
| Algae_064-2_Unigene_BMK.90003 |  | aca-miR-5p-43924 |
| Algae_064-2_Unigene_BMK.90247 |  | tae-miR159a |
| Algae_064-2_Unigene_BMK.90295 | Calcium-binding protein 7 OS=Rattus norvegicus GN=Cabp7 PE=1 SV=1 | zma-miR529-5p |
| Algae_064-2_Unigene_BMK.90561 | Hydroxysteroid dehydrogenase-like protein 2 OS=Rattus norvegicus GN=Hsdl2 PE=2 SV=1 | stu-miR171b-3p |
| Algae_064-2_Unigene_BMK.90625 |  | aca-miR-5p-43924 |
| Algae_064-2_Unigene_BMK.91080 | Aldose reductase OS=Sus scrofa GN=AKR1B1 PE=1 SV=2 | stu-miR171b-3p |
| Algae_064-2_Unigene_BMK.91160 |  | stu-miR171b-3p |
| Algae_064-2_Unigene_BMK.91260 |  | osa-miR168a-5p |
| Algae_064-2_Unigene_BMK.92106 | Putative protein TPRXL OS=Homo sapiens GN=TPRXL PE=5 SV=2 | zma-miR529-5p |
| Algae_064-2_Unigene_BMK.9211 |  | rgl-miR5139_L+3 |
| Algae_064-2_Unigene_BMK.92497 | Uncharacterized oxidoreductase YtbE OS=Bacillus subtilis GN=ytbE PE=1 SV=1 | bdi-miR7732-3p_L-1_1ss11GC |
| Algae_064-2_Unigene_BMK.92726 | Leucine-rich repeat-containing protein 40 OS=Mus musculus GN=Lrrc40 PE=2 SV=2 | bdi-miR7732-3p_L-1_1ss11GC |
| Algae_064-2_Unigene_BMK.92746 | Copper transport protein ATOX1 homolog OS=Dictyostelium discoideum GN=atox1 PE=3 SV=2 | sbi-miR169c |
| Algae_064-2_Unigene_BMK.92752 | DNA polymerase lambda OS=Homo sapiens GN=POLL PE=1 SV=1 | aca-miR-5p-43924 |
| Algae_064-2_Unigene_BMK.93037 | Chorismate mutase, chloroplastic OS=Arabidopsis thaliana GN=CM1 PE=2 SV=2 | aca-miR-3p-456915 |
| Algae_064-2_Unigene_BMK.9304 | Protein gar2 OS=Schizosaccharomyces pombe GN=gar2 PE=1 SV=2 | aca-miR-5p-43924 |
| Algae_064-2_Unigene_BMK.93623 |  | aca-miR-5p-43924 |
| Algae_064-2_Unigene_BMK.9368 | Serine/arginine repetitive matrix protein 2 OS=Homo sapiens GN=SRRM2 PE=1 SV=2 | aca-miR-5p-43924 |
| Algae_064-2_Unigene_BMK.93736 | Basic proline-rich protein OS=Sus scrofa PE=1 SV=2 | aca-miR-3p-456915 |
| Algae_064-2_Unigene_BMK.93842 | Serine/arginine repetitive matrix protein 2 OS=Homo sapiens GN=SRRM2 PE=1 SV=2 | aca-miR-5p-43924 |
| Algae_064-2_Unigene_BMK.94021 |  | aca-miR-3p-456915 |
| Algae_064-2_Unigene_BMK.94045 | 60S ribosomal protein L16 OS=Neurospora crassa GN=rpl-16 PE=3 SV=1 | osa-miR168a-5p |
| Algae_064-2_Unigene_BMK.94082 | WW domain-containing oxidoreductase OS=Mus musculus GN=Wwox PE=1 SV=1 | bdi-miR7732-3p_L-1_1ss11GC |
| Algae_064-2_Unigene_BMK.94113 | Basic proline-rich protein OS=Sus scrofa PE=1 SV=2 | stu-miR171b-3p |
| Algae_064-2_Unigene_BMK.94170 |  | sbi-miR169c |
| Algae_064-2_Unigene_BMK.9490 | Mannosyl-oligosaccharide 1,2-alpha-mannosidase IA OS=Mus musculus GN=Man1a1 PE=1 SV=1 | sbi-miR169c |
| Algae_064-2_Unigene_BMK.9543 |  | aca-miR-3p-456915 |
| Algae_064-2_Unigene_BMK.9679 | Serine/arginine repetitive matrix protein 2 OS=Homo sapiens GN=SRRM2 PE=1 SV=2 | rgl-miR5139_L+3 |
| Algae_064-2_Unigene_BMK.9694 | Probable exonuclease mut-7 homolog OS=Homo sapiens GN=EXD3 PE=1 SV=3 | sbi-miR169c |
| Algae_064-2_Unigene_BMK.9801 |  | osa-miR168a-5p |
| Algae_064-2_Unigene_BMK.9815 |  | sbi-miR169c |
| Algae_064-2_Unigene_BMK.9818 | Keratin-associated protein 5-4 OS=Mus musculus GN=Krtap5-4 PE=2 SV=1 | aca-miR-5p-43924 |
| Algae_064-2_Unigene_BMK.9913 | Transmembrane protein 110 OS=Rattus norvegicus GN=Tmem110 PE=2 SV=1 | sbi-miR169c |
| Algae_064-3_Unigene_BMK.1000 | Inositol-trisphosphate 3-kinase A OS=Homo sapiens GN=ITPKA PE=1 SV=1 | sbi-miR169c |
| Algae_064-3_Unigene_BMK.10461 |  | osa-miR168a-5p |
| Algae_064-3_Unigene_BMK.10548 | Translation initiation factor IF-2 OS=Streptomyces avermitilis GN=infB PE=3 SV=1 | sbi-miR169c |
| Algae_064-3_Unigene_BMK.1060 | Peptidyl-prolyl cis-trans isomerase 1 OS=Brugia malayi GN=CYP-1 PE=1 SV=1 | sbi-miR169c |
| Algae_064-3_Unigene_BMK.10633 | ATP-dependent zinc metalloprotease FtsH OS=Porphyra yezoensis GN=ftsH PE=3 SV=1 | aca-miR-3p-456915 |
| Algae_064-3_Unigene_BMK.10897 | Uncharacterized protein BHLF1 OS=Epstein-Barr virus (strain B95-8) GN=BHLF1 PE=4 SV=1 | sbi-miR169c |
| Algae_064-3_Unigene_BMK.1097 | Sodium-coupled neutral amino acid transporter 1 OS=Mus musculus GN=Slc38a1 PE=1 SV=1 | rgl-miR5139_L+3 |
| Algae_064-3_Unigene_BMK.11001 | Uncharacterized serine/threonine-rich protein PB15E9.01c OS=Schizosaccharomyces pombe GN=SPAPB15E9.01c PE=2 SV=2 | sbi-miR169c |
| Algae_064-3_Unigene_BMK.11040 | Translation initiation factor IF-2 OS=Frankia sp. (strain EAN1pec) GN=infB PE=3 SV=1 | aca-miR-3p-456915 |
| Algae_064-3_Unigene_BMK.11173 | Branchpoint-bridging protein OS=Debaryomyces hansenii GN=BBP PE=3 SV=2 | zma-miR529-5p |
| Algae_064-3_Unigene_BMK.11205 |  | aca-miR-3p-456915 |
| Algae_064-3_Unigene_BMK.11243 | Probable steroid-binding protein 3 OS=Arabidopsis thaliana GN=MP3 PE=1 SV=1 | osa-miR168a-5p |
| Algae_064-3_Unigene_BMK.11464 | Protein FAM119A OS=Danio rerio GN=fam119a PE=2 SV=1 | sbi-miR169c |
| Algae_064-3_Unigene_BMK.11605 | Serine/threonine-protein phosphatase 2B catalytic subunit 1 OS=Drosophila melanogaster GN=CanA1 PE=1 SV=2 | stu-miR171b-3p |
| Algae_064-3_Unigene_BMK.11641 | 1-deoxy-D-xylulose-5-phosphate synthase OS=Rhodopseudomonas palustris (strain BisB18) GN=dxs PE=3 SV=1 | sbi-miR169c |
| Algae_064-3_Unigene_BMK.11694 |  | stu-miR171b-3p |
| Algae_064-3_Unigene_BMK.11792 | Uncharacterized protein DKFZp434B061 OS=Homo sapiens PE=2 SV=2 | zma-miR529-5p |
| Algae_064-3_Unigene_BMK.12016 | Serine/threonine-protein kinase HT1 OS=Arabidopsis thaliana GN=HT1 PE=1 SV=1 | stu-miR171b-3p |
| Algae_064-3_Unigene_BMK.12183 | Translation initiation factor IF-2 OS=Kineococcus radiotolerans (strain ATCC BAA-149 / DSM 14245 / SRS30216) GN=infB PE=3 SV=1 | zma-miR529-5p |
| Algae_064-3_Unigene_BMK.12230 |  | sbi-miR169c |
| Algae_064-3_Unigene_BMK.1236 | Speckle-type POZ protein OS=Xenopus tropicalis GN=spop PE=2 SV=1 | rgl-miR5139_L+3 |
| Algae_064-3_Unigene_BMK.1252 | Mucin-5AC (Fragments) OS=Homo sapiens GN=MUC5AC PE=1 SV=3 | sbi-miR169c |
| Algae_064-3_Unigene_BMK.12529 | E3 ubiquitin-protein ligase MARCH9 OS=Homo sapiens GN=MARCH9 PE=1 SV=2 | aca-miR-3p-456915 |
| Algae_064-3_Unigene_BMK.1265 | Adenylate cyclase type 4 OS=Homo sapiens GN=ADCY4 PE=1 SV=1 | rgl-miR5139_L+3 |
| Algae_064-3_Unigene_BMK.12914 | Arginine and glutamate-rich protein 1 OS=Rattus norvegicus GN=Arglu1 PE=2 SV=1 | aca-miR-5p-43924 |
| Algae_064-3_Unigene_BMK.12944 |  | osa-miR168a-5p |
| Algae_064-3_Unigene_BMK.1306 | Mucin-19 OS=Mus musculus GN=Muc19 PE=2 SV=2 | bdi-miR7732-3p_L-1_1ss11GC |
| Algae_064-3_Unigene_BMK.1309 | Translation initiation factor IF-2 OS=Streptomyces coelicolor GN=infB PE=3 SV=1 | osa-miR168a-5p |
| Algae_064-3_Unigene_BMK.13186 |  | aca-miR-3p-456915 |
| Algae_064-3_Unigene_BMK.13370 |  | osa-miR168a-5p |
| Algae_064-3_Unigene_BMK.13531 | Glutaredoxin OS=Vernicia fordii PE=3 SV=1 | zma-miR529-5p |
| Algae_064-3_Unigene_BMK.13546 | Uncharacterized protein TC_0114 OS=Chlamydia muridarum GN=TC_0114 PE=4 SV=2 | osa-miR168a-5p |
| Algae_064-3_Unigene_BMK.13644 |  | zma-miR529-5p |
| Algae_064-3_Unigene_BMK.13752 | Putative protein TPRXL OS=Homo sapiens GN=TPRXL PE=5 SV=2 | bdi-miR7732-3p_L-1_1ss11GC |
| Algae_064-3_Unigene_BMK.13759 | Serine/arginine repetitive matrix protein 2 OS=Homo sapiens GN=SRRM2 PE=1 SV=2 | aca-miR-5p-43924 |
| Algae_064-3_Unigene_BMK.13802 | Uncharacterized serine/threonine-rich protein PB15E9.01c OS=Schizosaccharomyces pombe GN=SPAPB15E9.01c PE=2 SV=2 | sbi-miR169c |
| Algae_064-3_Unigene_BMK.13835 | Putative protein TPRXL OS=Homo sapiens GN=TPRXL PE=5 SV=2 | sbi-miR169c |
| Algae_064-3_Unigene_BMK.13917 | Protein farnesyltransferase subunit beta OS=Mus musculus GN=Fntb PE=2 SV=1 | sbi-miR169c |
| Algae_064-3_Unigene_BMK.13972 | Kinesin-associated protein 3 OS=Homo sapiens GN=KIFAP3 PE=1 SV=2 | rgl-miR5139_L+3 |
| Algae_064-3_Unigene_BMK.14140 |  | sbi-miR169c |
| Algae_064-3_Unigene_BMK.14296 | Putative ariadne-like RING finger protein R811 OS=Acanthamoeba polyphaga mimivirus GN=MIMI_R811 PE=4 SV=1 | zma-miR529-5p |
| Algae_064-3_Unigene_BMK.1438 | RING-H2 finger protein ATL43 OS=Arabidopsis thaliana GN=ATL43 PE=2 SV=2 | bdi-miR7732-3p_L-1_1ss11GC |
| Algae_064-3_Unigene_BMK.14384 | Phosphatidylinositol-4-phosphate 5-kinase 5 OS=Arabidopsis thaliana GN=PIP5K5 PE=2 SV=1 | osa-miR168a-5p |
| Algae_064-3_Unigene_BMK.14494 | Uncharacterized protein DKFZp434B061 OS=Homo sapiens PE=2 SV=2 | stu-miR171b-3p |
| Algae_064-3_Unigene_BMK.14498 | Uncharacterized protein LF3 OS=Epstein-Barr virus (strain GD1) GN=LF3 PE=3 SV=1 | stu-miR171b-3p |
| Algae_064-3_Unigene_BMK.1457 | Tetra-peptide repeat homeobox protein 1 OS=Homo sapiens GN=TPRX1 PE=2 SV=3 | sbi-miR169c |
| Algae_064-3_Unigene_BMK.14783 |  | aca-miR-3p-456915 |
| Algae_064-3_Unigene_BMK.14815 |  | rgl-miR5139_L+3 |
| Algae_064-3_Unigene_BMK.14888 | Serine/threonine-protein kinase SIK1 OS=Homo sapiens GN=SIK1 PE=1 SV=2 | aca-miR-3p-456915 |
| Algae_064-3_Unigene_BMK.14897 | Calcium-dependent protein kinase 3 OS=Plasmodium falciparum (isolate 3D7) GN=CPK3 PE=1 SV=1 | sbi-miR169c |
| Algae_064-3_Unigene_BMK.14930 | Uncharacterized serine-rich protein C215.13 OS=Schizosaccharomyces pombe GN=SPBC215.13 PE=1 SV=1 | zma-miR529-5p |
| Algae_064-3_Unigene_BMK.15039 | Protein CbbY OS=Rhodobacter sphaeroides GN=cbbY PE=3 SV=1 | aca-miR-3p-456915 |
| Algae_064-3_Unigene_BMK.15065 | Basic proline-rich protein OS=Sus scrofa PE=1 SV=2 | stu-miR171b-3p |
| Algae_064-3_Unigene_BMK.15098 | Sialin OS=Homo sapiens GN=SLC17A5 PE=1 SV=2 | aca-miR-3p-456915 |
| Algae_064-3_Unigene_BMK.15161 | Uncharacterized protein BHLF1 OS=Epstein-Barr virus (strain B95-8) GN=BHLF1 PE=4 SV=1 | zma-miR529-5p |
| Algae_064-3_Unigene_BMK.15472 | Uncharacterized protein DKFZp434B061 OS=Homo sapiens PE=2 SV=2 | aca-miR-3p-456915 |
| Algae_064-3_Unigene_BMK.15653 | Uncharacterized 35.5 kDa protein in transposon Tn4556 OS=Streptomyces fradiae PE=4 SV=1 | aca-miR-3p-456915 |
| Algae_064-3_Unigene_BMK.15708 | Basic proline-rich protein OS=Sus scrofa PE=1 SV=2 | aca-miR-5p-43924 |
| Algae_064-3_Unigene_BMK.15826 | Myosin-1 OS=Equus caballus GN=MYH1 PE=2 SV=1 | rgl-miR5139_L+3 |
| Algae_064-3_Unigene_BMK.15855 | Translation initiation factor IF-2 OS=Streptomyces coelicolor GN=infB PE=3 SV=1 | sbi-miR169c |
| Algae_064-3_Unigene_BMK.15908 | Peroxisomal acyl-coenzyme A oxidase 1 OS=Rattus norvegicus GN=Acox1 PE=1 SV=1 | aca-miR-5p-43924 |
| Algae_064-3_Unigene_BMK.1599 | Serine/arginine repetitive matrix protein 2 OS=Mus musculus GN=Srrm2 PE=1 SV=2 | zma-miR529-5p |
| Algae_064-3_Unigene_BMK.16054 | Probable endo-beta-1,4-glucanase celB OS=Neosartorya fischeri (strain ATCC 1020 / DSM 3700 / FGSC A1164 / NRRL 181) GN=celB PE=3 SV=1 | aca-miR-5p-43924 |
| Algae_064-3_Unigene_BMK.16402 | Flap endonuclease 1 OS=Glycine max GN=FEN1 PE=2 SV=1 | osa-miR2876-3p_R+1 |
| Algae_064-3_Unigene_BMK.16632 |  | aca-miR-3p-456915 |
| Algae_064-3_Unigene_BMK.16725 | Uncharacterized serine/threonine-rich protein PB15E9.01c OS=Schizosaccharomyces pombe GN=SPAPB15E9.01c PE=2 SV=2 | aca-miR-3p-456915 |
| Algae_064-3_Unigene_BMK.16740 | Actin cytoskeleton-regulatory complex protein pan1 OS=Aspergillus clavatus (strain ATCC 1007 / CBS 513.65 / DSM 816 / NCTC 3887 / NRRL 1) GN=pan1 PE=3 SV=1 | zma-miR529-5p |
| Algae_064-3_Unigene_BMK.16825 | Alpha-ketoglutarate-dependent taurine dioxygenase OS=Escherichia coli (strain K12) GN=tauD PE=1 SV=3 | osa-miR168a-5p |
| Algae_064-3_Unigene_BMK.16990 | Thylakoid membrane protein slr0575 OS=Synechocystis sp. (strain ATCC 27184 / PCC 6803 / N-1) GN=slr0575 PE=4 SV=1 | aca-miR-5p-43924 |
| Algae_064-3_Unigene_BMK.17066 | Poly [ADP-ribose] polymerase 1 OS=Gallus gallus GN=PARP1 PE=1 SV=2 | aca-miR-3p-456915 |
| Algae_064-3_Unigene_BMK.17177 |  | aca-miR-3p-456915 |
| Algae_064-3_Unigene_BMK.17268 | Casein kinase I OS=Toxoplasma gondii PE=2 SV=1 | stu-miR171b-3p |
| Algae_064-3_Unigene_BMK.1727 | Uncharacterized protein R166.3 OS=Caenorhabditis elegans GN=R166.3 PE=2 SV=1 | sbi-miR169c |
| Algae_064-3_Unigene_BMK.1747 | Putative protein TPRXL OS=Homo sapiens GN=TPRXL PE=5 SV=2 | osa-miR168a-5p |
| Algae_064-3_Unigene_BMK.17492 | Serine/arginine repetitive matrix protein 1 OS=Mus musculus GN=Srrm1 PE=1 SV=1 | rgl-miR5139_L+3 |
| Algae_064-3_Unigene_BMK.17540 | Uncharacterized protein DDB_G0271670 OS=Dictyostelium discoideum GN=DDB_G0271670 PE=4 SV=1 | aca-miR-5p-43924 |
| Algae_064-3_Unigene_BMK.17548 | Haloacid dehalogenase-like hydrolase domain-containing protein 2 OS=Rattus norvegicus GN=Hdhd2 PE=2 SV=1 | tae-miR159a |
| Algae_064-3_Unigene_BMK.1755 | BTB/POZ domain-containing protein KCTD12 OS=Homo sapiens GN=KCTD12 PE=1 SV=1 | sbi-miR169c |
| Algae_064-3_Unigene_BMK.17658 | Pentatricopeptide repeat-containing protein At1g62930, chloroplastic OS=Arabidopsis thaliana GN=At1g62930 PE=2 SV=2 | aca-miR-5p-43924 |
| Algae_064-3_Unigene_BMK.17661 | Phosphoethanolamine N-methyltransferase 1 OS=Arabidopsis thaliana GN=NMT1 PE=2 SV=1 | zma-miR529-5p |
| Algae_064-3_Unigene_BMK.17747 | FK506-binding protein 4 OS=Rhizopus oryzae GN=FKBP4 PE=3 SV=1 | bdi-miR7732-3p_L-1_1ss11GC |
| Algae_064-3_Unigene_BMK.17796 | Dapper homolog 3 OS=Mus musculus GN=Dact3 PE=2 SV=1 | stu-miR171b-3p |
| Algae_064-3_Unigene_BMK.17816 | Uncharacterized 35.5 kDa protein in transposon Tn4556 OS=Streptomyces fradiae PE=4 SV=1 | aca-miR-5p-43924 |
| Algae_064-3_Unigene_BMK.1798 | Translation initiation factor IF-2 OS=Frankia alni (strain ACN14a) GN=infB PE=3 SV=1 | osa-miR168a-5p |
| Algae_064-3_Unigene_BMK.18038 | Translation initiation factor IF-2 OS=Frankia sp. (strain EAN1pec) GN=infB PE=3 SV=1 | rgl-miR5139_L+3 |
| Algae_064-3_Unigene_BMK.18075 | SH3 domain-binding protein 1 OS=Homo sapiens GN=SH3BP1 PE=1 SV=3 | sbi-miR169c |
| Algae_064-3_Unigene_BMK.18170 | Peptide methionine sulfoxide reductase OS=Drosophila melanogaster GN=Eip71CD PE=2 SV=2 | bdi-miR7732-3p_L-1_1ss11GC |
| Algae_064-3_Unigene_BMK.18197 | Splicing factor, arginine/serine-rich 19 OS=Homo sapiens GN=SCAF1 PE=1 SV=3 | aca-miR-5p-43924 |
| Algae_064-3_Unigene_BMK.18252 | Potassium voltage-gated channel subfamily H member 6 OS=Homo sapiens GN=KCNH6 PE=1 SV=1 | stu-miR171b-3p |
| Algae_064-3_Unigene_BMK.18293 | Mucin-19 OS=Mus musculus GN=Muc19 PE=2 SV=2 | sbi-miR169c |
| Algae_064-3_Unigene_BMK.18349 |  | aca-miR-3p-456915 |
| Algae_064-3_Unigene_BMK.1848 | Ataxin-2 OS=Homo sapiens GN=ATXN2 PE=1 SV=2 | rgl-miR5139_L+3 |
| Algae_064-3_Unigene_BMK.18588 |  | aca-miR-5p-43924 |
| Algae_064-3_Unigene_BMK.18675 | Lipase maturation factor 1 OS=Homo sapiens GN=LMF1 PE=1 SV=1 | aca-miR-3p-456915 |
| Algae_064-3_Unigene_BMK.18705 | Ice-structuring protein 4 OS=Pseudopleuronectes americanus PE=3 SV=1 | osa-miR168a-5p |
| Algae_064-3_Unigene_BMK.18774 |  | sbi-miR169c |
| Algae_064-3_Unigene_BMK.18920 | Uncharacterized protein BHLF1 OS=Epstein-Barr virus (strain B95-8) GN=BHLF1 PE=4 SV=1 | stu-miR171b-3p |
| Algae_064-3_Unigene_BMK.19092 |  | sbi-miR169c |
| Algae_064-3_Unigene_BMK.19132 |  | sbi-miR169c |
| Algae_064-3_Unigene_BMK.1925 | Translation initiation factor IF-2 OS=Corynebacterium glutamicum GN=infB PE=3 SV=1 | sbi-miR169c |
| Algae_064-3_Unigene_BMK.19532 | Protein dispatched homolog 1 OS=Mus musculus GN=Disp1 PE=1 SV=2 | aca-miR-3p-456915 |
| Algae_064-3_Unigene_BMK.19642 | Keratin-associated protein 4-5 OS=Homo sapiens GN=KRTAP4-5 PE=2 SV=3 | stu-miR171b-3p |
| Algae_064-3_Unigene_BMK.20246 | 5E5 antigen OS=Rattus norvegicus PE=2 SV=1 | aca-miR-5p-43924 |
| Algae_064-3_Unigene_BMK.20434 |  | zma-miR529-5p |
| Algae_064-3_Unigene_BMK.20590 |  | stu-miR171b-3p |
| Algae_064-3_Unigene_BMK.20694 |  | osa-miR168a-5p |
| Algae_064-3_Unigene_BMK.20799 | Immediate-early protein IE180 OS=Suid herpesvirus 1 (strain Kaplan) GN=IE PE=3 SV=1 | osa-miR168a-5p |
| Algae_064-3_Unigene_BMK.20945 | Probable endo-beta-1,4-glucanase celB OS=Neosartorya fischeri (strain ATCC 1020 / DSM 3700 / FGSC A1164 / NRRL 181) GN=celB PE=3 SV=1 | zma-miR529-5p |
| Algae_064-3_Unigene_BMK.21260 | Dynein light chain Tctex-type 1 OS=Rattus norvegicus GN=Dynlt1 PE=1 SV=1 | sbi-miR169c |
| Algae_064-3_Unigene_BMK.21324 | 2-hydroxymuconic semialdehyde hydrolase OS=Pseudomonas sp. (strain CF600) GN=dmpD PE=3 SV=1 | sbi-miR169c |
| Algae_064-3_Unigene_BMK.21471 | Probable malate:quinone oxidoreductase OS=Kocuria rhizophila (strain ATCC 9341 / DSM 348 / NBRC 103217 / DC2201) GN=mqo PE=3 SV=1 | rgl-miR5139_L+3 |
| Algae_064-3_Unigene_BMK.2161 | Putative protein TPRXL OS=Homo sapiens GN=TPRXL PE=5 SV=2 | aca-miR-5p-43924 |
| Algae_064-3_Unigene_BMK.21749 | Ankyrin-1 OS=Mus musculus GN=Ank1 PE=1 SV=2 | osa-miR168a-5p |
| Algae_064-3_Unigene_BMK.21951 | N-alpha-acetyltransferase 15, NatA auxiliary subunit OS=Mus musculus GN=Naa15 PE=1 SV=1 | aca-miR-5p-43924 |
| Algae_064-3_Unigene_BMK.22185 | 50S ribosomal protein L22 OS=Acaryochloris marina (strain MBIC 11017) GN=rplV PE=3 SV=1 | sbi-miR169c |
| Algae_064-3_Unigene_BMK.22630 |  | aca-miR-3p-456915 |
| Algae_064-3_Unigene_BMK.22707 | Uncharacterized protein KIAA0802 OS=Homo sapiens GN=KIAA0802 PE=1 SV=4 | aca-miR-5p-43924 |
| Algae_064-3_Unigene_BMK.22820 | Putative protein TPRXL OS=Homo sapiens GN=TPRXL PE=5 SV=2 | aca-miR-5p-43924 |
| Algae_064-3_Unigene_BMK.22922 | Alternative oxidase, mitochondrial OS=Cryptococcus neoformans var. grubii GN=AOX1 PE=3 SV=1 | aca-miR-5p-43924 |
| Algae_064-3_Unigene_BMK.23016 | Probable syntaxin-8B OS=Dictyostelium discoideum GN=syn8B PE=3 SV=1 | aca-miR-3p-456915 |
| Algae_064-3_Unigene_BMK.23247 |  | aca-miR-3p-456915 |
| Algae_064-3_Unigene_BMK.2338 | Ribulose bisphosphate carboxylase (Fragment) OS=Prorocentrum minimum GN=rbcL PE=2 SV=1 | stu-miR171b-3p |
| Algae_064-3_Unigene_BMK.23394 | Putative uncharacterized protein ENSP00000383309 OS=Homo sapiens PE=5 SV=3 | osa-miR168a-5p |
| Algae_064-3_Unigene_BMK.23764 | Homoserine O-acetyltransferase OS=Stenotrophomonas maltophilia (strain K279a) GN=metX PE=3 SV=1 | sbi-miR169c |
| Algae_064-3_Unigene_BMK.2391 |  | sbi-miR169c |
| Algae_064-3_Unigene_BMK.23975 | Cathepsin E OS=Rattus norvegicus GN=Ctse PE=1 SV=3 | zma-miR529-5p |
| Algae_064-3_Unigene_BMK.2405 | Protein notum homolog OS=Homo sapiens GN=NOTUM PE=2 SV=2 | sbi-miR169c |
| Algae_064-3_Unigene_BMK.24116 | Pyruvate carboxylase OS=Bacillus subtilis GN=pyc PE=2 SV=1 | rgl-miR5139_L+3 |
| Algae_064-3_Unigene_BMK.2415 | Meiosis protein mei2 OS=Schizosaccharomyces pombe GN=mei2 PE=1 SV=1 | aca-miR-5p-43924 |
| Algae_064-3_Unigene_BMK.24405 | WD repeat-containing protein 35 OS=Rattus norvegicus GN=Wdr35 PE=1 SV=1 | aca-miR-5p-43924 |
| Algae_064-3_Unigene_BMK.2443 | 50S ribosomal protein L14 OS=Gemmatimonas aurantiaca (strain T-27 / DSM 14586 / JCM 11422 / NBRC 100505) GN=rplN PE=3 SV=1 | sbi-miR169c |
| Algae_064-3_Unigene_BMK.24526 | Probable glutathione peroxidase 2 OS=Arabidopsis thaliana GN=GPX2 PE=1 SV=1 | bdi-miR7732-3p_L-1_1ss11GC |
| Algae_064-3_Unigene_BMK.24550 |  | stu-miR171b-3p |
| Algae_064-3_Unigene_BMK.24711 | Metacaspase-1 OS=Ustilago maydis GN=MCA1 PE=3 SV=1 | sbi-miR169c |
| Algae_064-3_Unigene_BMK.24822 | Mucin-19 OS=Mus musculus GN=Muc19 PE=2 SV=2 | aca-miR-5p-43924 |
| Algae_064-3_Unigene_BMK.25028 |  | aca-miR-3p-456915 |
| Algae_064-3_Unigene_BMK.25104 | Gibberellin 20 oxidase 2 OS=Oryza sativa subsp. japonica GN=20ox2 PE=1 SV=1 | rgl-miR5139_L+3 |
| Algae_064-3_Unigene_BMK.25113 | Translation initiation factor IF-2 OS=Nocardioides sp. (strain BAA-499 / JS614) GN=infB PE=3 SV=1 | osa-miR168a-5p |
| Algae_064-3_Unigene_BMK.2535 |  | aca-miR-3p-456915 |
| Algae_064-3_Unigene_BMK.25442 | Formin-like protein 5 OS=Oryza sativa subsp. japonica GN=FH5 PE=2 SV=2 | aca-miR-5p-43924 |
| Algae_064-3_Unigene_BMK.25573 |  | stu-miR171b-3p |
| Algae_064-3_Unigene_BMK.25832 |  | tae-miR159a |
| Algae_064-3_Unigene_BMK.26004 | Glycoprotein gp2 OS=Equine herpesvirus 1 (strain Ab4p) GN=EUs4 PE=4 SV=1 | sbi-miR169c |
| Algae_064-3_Unigene_BMK.26481 | Uncharacterized protein LF3 OS=Epstein-Barr virus (strain GD1) GN=LF3 PE=3 SV=1 | zma-miR529-5p |
| Algae_064-3_Unigene_BMK.26711 |  | zma-miR529-5p |
| Algae_064-3_Unigene_BMK.26773 | Uncharacterized 29.3 kDa protein OS=Orgyia pseudotsugata multicapsid polyhedrosis virus GN=ORF92 PE=4 SV=1 | stu-miR171b-3p |
| Algae_064-3_Unigene_BMK.26899 | Carnosine synthase 1 OS=Gallus gallus GN=CARNS1 PE=1 SV=1 | bdi-miR7732-3p_L-1_1ss11GC |
| Algae_064-3_Unigene_BMK.27194 | Putative protein TPRXL OS=Homo sapiens GN=TPRXL PE=5 SV=2 | zma-miR529-5p |
| Algae_064-3_Unigene_BMK.2720 |  | zma-miR529-5p |
| Algae_064-3_Unigene_BMK.27364 | Extracellular serine proteinase OS=Thermus sp. (strain Rt41A) PE=1 SV=3 | aca-miR-5p-43924 |
| Algae_064-3_Unigene_BMK.27444 | Poly(ADP-ribose) glycohydrolase ARH3 OS=Gallus gallus GN=ADPRHL2 PE=2 SV=1 | osa-miR168a-5p |
| Algae_064-3_Unigene_BMK.27489 | Guanine nucleotide-binding protein G(s) subunit alpha isoforms XLas OS=Rattus norvegicus GN=Gnas PE=1 SV=3 | sbi-miR169c |
| Algae_064-3_Unigene_BMK.27659 | ESX-1 secretion-associated protein EspI OS=Mycobacterium tuberculosis GN=espI PE=4 SV=1 | osa-miR168a-5p |
| Algae_064-3_Unigene_BMK.27667 | Uncharacterized protein BHLF1 OS=Epstein-Barr virus (strain B95-8) GN=BHLF1 PE=4 SV=1 | aca-miR-5p-43924 |
| Algae_064-3_Unigene_BMK.27837 | Nucleosome assembly protein 1-like 1 OS=Xenopus tropicalis GN=nap1l1 PE=2 SV=1 | osa-miR168a-5p |
| Algae_064-3_Unigene_BMK.27868 | Putative aliphatic sulfonates-binding protein OS=Bacillus subtilis GN=ssuA PE=2 SV=1 | bdi-miR7732-3p_L-1_1ss11GC |
| Algae_064-3_Unigene_BMK.27956 | Probable elongation factor 1-gamma 1 OS=Arabidopsis thaliana GN=At1g09640 PE=2 SV=1 | stu-miR171b-3p |
| Algae_064-3_Unigene_BMK.28199 | Serine/arginine repetitive matrix protein 1 OS=Pongo abelii GN=SRRM1 PE=2 SV=1 | sbi-miR169c |
| Algae_064-3_Unigene_BMK.28344 | Translation initiation factor IF-2 OS=Rhodococcus erythropolis (strain PR4 / NBRC 100887) GN=infB PE=3 SV=1 | osa-miR168a-5p |
| Algae_064-3_Unigene_BMK.28463 | Translation initiation factor IF-2 OS=Streptomyces griseus subsp. griseus (strain JCM 4626 / NBRC 13350) GN=infB PE=3 SV=1 | aca-miR-5p-43924 |
| Algae_064-3_Unigene_BMK.28721 | Chitin-binding lectin 1 OS=Solanum tuberosum PE=1 SV=2 | zma-miR529-5p |
| Algae_064-3_Unigene_BMK.29052 | Carnosine synthase 1 OS=Gallus gallus GN=CARNS1 PE=1 SV=1 | aca-miR-3p-456915 |
| Algae_064-3_Unigene_BMK.2909 | E3 ubiquitin ligase complex SCF subunit scon-3 OS=Neurospora crassa GN=scon-3 PE=1 SV=1 | sbi-miR169c |
| Algae_064-3_Unigene_BMK.29107 | Putative protein TPRXL OS=Homo sapiens GN=TPRXL PE=5 SV=2 | aca-miR-5p-43924 |
| Algae_064-3_Unigene_BMK.29255 | Uncharacterized ABC transporter ATP-binding protein Rv1819c/MT1867 OS=Mycobacterium tuberculosis GN=Rv1819c PE=3 SV=1 | aca-miR-5p-43924 |
| Algae_064-3_Unigene_BMK.29385 | Serine/arginine repetitive matrix protein 1 OS=Pongo abelii GN=SRRM1 PE=2 SV=1 | sbi-miR169c |
| Algae_064-3_Unigene_BMK.29414 | Putative mitochondrial carrier protein PET8 OS=Ashbya gossypii (strain ATCC 10895 / CBS 109.51 / FGSC 9923 / NRRL Y-1056) GN=PET8 PE=3 SV=1 | rgl-miR5139_L+3 |
| Algae_064-3_Unigene_BMK.29493 | DnaJ protein homolog ANJ1 OS=Atriplex nummularia PE=2 SV=1 | osa-miR168a-5p |
| Algae_064-3_Unigene_BMK.29532 |  | sbi-miR169c |
| Algae_064-3_Unigene_BMK.29597 | Malonyl-CoA-acyl carrier protein transacylase, mitochondrial OS=Mus musculus GN=Mcat PE=2 SV=3 | aca-miR-3p-456915 |
| Algae_064-3_Unigene_BMK.29732 | Probable mitochondrial-processing peptidase subunit beta OS=Schizosaccharomyces pombe GN=qcr1 PE=2 SV=1 | stu-miR171b-3p |
| Algae_064-3_Unigene_BMK.30042 | Alpha-glucan water dikinase, chloroplastic OS=Solanum tuberosum GN=R1 PE=1 SV=2 | sbi-miR169c |
| Algae_064-3_Unigene_BMK.30106 | Poly [ADP-ribose] polymerase 15 OS=Homo sapiens GN=PARP15 PE=1 SV=1 | aca-miR-5p-43924 |
| Algae_064-3_Unigene_BMK.30120 | Vegetative cell wall protein gp1 OS=Chlamydomonas reinhardtii GN=GP1 PE=2 SV=1 | zma-miR529-5p |
| Algae_064-3_Unigene_BMK.30198 |  | osa-miR168a-5p |
| Algae_064-3_Unigene_BMK.30271 | mRNA-decapping enzyme-like protein OS=Arabidopsis thaliana GN=At1g08370 PE=2 SV=2 | sbi-miR169c |
| Algae_064-3_Unigene_BMK.30370 | Atherin OS=Oryctolagus cuniculus GN=SAMD1 PE=2 SV=1 | aca-miR-3p-456915 |
| Algae_064-3_Unigene_BMK.30475 |  | aca-miR-3p-456915 |
| Algae_064-3_Unigene_BMK.30479 | Uncharacterized protein LOC284861 OS=Homo sapiens PE=2 SV=1 | aca-miR-5p-43924 |
| Algae_064-3_Unigene_BMK.30511 | Ankycorbin OS=Rattus norvegicus GN=Rai14 PE=2 SV=2 | osa-miR2876-3p_R+1 |
| Algae_064-3_Unigene_BMK.30537 |  | aca-miR-3p-456915 |
| Algae_064-3_Unigene_BMK.30596 |  | aca-miR-5p-43924 |
| Algae_064-3_Unigene_BMK.30709 | Meiosis protein mei2 OS=Schizosaccharomyces pombe GN=mei2 PE=1 SV=1 | aca-miR-3p-456915 |
| Algae_064-3_Unigene_BMK.30758 | Tetratricopeptide repeat protein 28 OS=Homo sapiens GN=TTC28 PE=1 SV=4 | zma-miR529-5p |
| Algae_064-3_Unigene_BMK.3080 | Rab9 effector protein with kelch motifs OS=Bos taurus GN=RABEPK PE=2 SV=1 | bdi-miR7732-3p_L-1_1ss11GC |
| Algae_064-3_Unigene_BMK.30834 | Importin-5 OS=Homo sapiens GN=IPO5 PE=1 SV=4 | sbi-miR169c |
| Algae_064-3_Unigene_BMK.30874 | RING finger protein 44 OS=Homo sapiens GN=RNF44 PE=2 SV=1 | osa-miR168a-5p |
| Algae_064-3_Unigene_BMK.3089 | Putative uridine kinase C227.14 OS=Schizosaccharomyces pombe GN=SPAC227.14 PE=2 SV=1 | stu-miR171b-3p |
| Algae_064-3_Unigene_BMK.31108 |  | stu-miR171b-3p |
| Algae_064-3_Unigene_BMK.31177 | Uncharacterized protein BHLF1 OS=Epstein-Barr virus (strain B95-8) GN=BHLF1 PE=4 SV=1 | zma-miR529-5p |
| Algae_064-3_Unigene_BMK.31420 | Translation initiation factor IF-2 OS=Rhodococcus sp. (strain RHA1) GN=infB PE=3 SV=1 | stu-miR171b-3p |
| Algae_064-3_Unigene_BMK.31541 | Myb-like protein P OS=Dictyostelium discoideum GN=mybP PE=3 SV=1 | aca-miR-5p-43924 |
| Algae_064-3_Unigene_BMK.31619 | Translation initiation factor IF-2 OS=Mycobacterium marinum (strain ATCC BAA-535 / M) GN=infB PE=3 SV=1 | stu-miR171b-3p |
| Algae_064-3_Unigene_BMK.31825 | Integral membrane protein GPR155 OS=Homo sapiens GN=GPR155 PE=1 SV=2 | stu-miR171b-3p |
| Algae_064-3_Unigene_BMK.31919 | Keratinocyte proline-rich protein OS=Rattus norvegicus GN=Kprp PE=2 SV=1 | zma-miR529-5p |
| Algae_064-3_Unigene_BMK.3196 | Translation initiation factor IF-2 OS=Rhodococcus opacus (strain B4) GN=infB PE=3 SV=1 | sbi-miR169c |
| Algae_064-3_Unigene_BMK.32179 |  | zma-miR529-5p |
| Algae_064-3_Unigene_BMK.32196 |  | tae-miR159a |
| Algae_064-3_Unigene_BMK.32289 |  | aca-miR-5p-43924 |
| Algae_064-3_Unigene_BMK.32310 |  | stu-miR171b-3p |
| Algae_064-3_Unigene_BMK.32358 | Beta-glucosidase OS=Rhizobium radiobacter GN=cbg-1 PE=3 SV=1 | bdi-miR7732-3p_L-1_1ss11GC |
| Algae_064-3_Unigene_BMK.3247 | Translation initiation factor IF-2 OS=Synechococcus sp. (strain CC9311) GN=infB PE=3 SV=1 | aca-miR-5p-43924 |
| Algae_064-3_Unigene_BMK.325 | Peptidyl-prolyl isomerase FKBP12 OS=Arabidopsis thaliana GN=FKBP12 PE=1 SV=2 | zma-miR529-5p |
| Algae_064-3_Unigene_BMK.32594 | Proteasome assembly chaperone 4 OS=Mus musculus GN=Psmg4 PE=1 SV=1 | sbi-miR169c |
| Algae_064-3_Unigene_BMK.32596 | Calmodulin OS=Prorocentrum minimum PE=2 SV=1 | osa-miR168a-5p |
| Algae_064-3_Unigene_BMK.32625 | Basic proline-rich protein OS=Sus scrofa PE=1 SV=2 | aca-miR-5p-43924 |
| Algae_064-3_Unigene_BMK.32683 | Serine/arginine repetitive matrix protein 2 OS=Homo sapiens GN=SRRM2 PE=1 SV=2 | aca-miR-3p-456915 |
| Algae_064-3_Unigene_BMK.32724 | Wiskott-Aldrich syndrome protein homolog 1 OS=Schizosaccharomyces pombe GN=wsp1 PE=1 SV=3 | aca-miR-3p-456915 |
| Algae_064-3_Unigene_BMK.32856 | Flocculation protein FLO11 OS=Saccharomyces cerevisiae GN=MUC1 PE=1 SV=2 | sbi-miR169c |
| Algae_064-3_Unigene_BMK.3292 | Translation initiation factor IF-2 OS=Synechococcus sp. (strain CC9311) GN=infB PE=3 SV=1 | sbi-miR169c |
| Algae_064-3_Unigene_BMK.3303 |  | stu-miR171b-3p |
| Algae_064-3_Unigene_BMK.33636 |  | zma-miR529-5p |
| Algae_064-3_Unigene_BMK.33706 | Atherin OS=Oryctolagus cuniculus GN=SAMD1 PE=2 SV=1 | sbi-miR169c |
| Algae_064-3_Unigene_BMK.33715 | Translation initiation factor IF-2 OS=Micrococcus luteus (strain ATCC 4698 / IFO 3333 / NCTC 2665) GN=infB PE=3 SV=1 | osa-miR168a-5p |
| Algae_064-3_Unigene_BMK.34065 | Ubiquitin-like protein OS=Orgyia pseudotsugata multicapsid polyhedrosis virus GN=V-UBI PE=3 SV=1 | sbi-miR169c |
| Algae_064-3_Unigene_BMK.34091 | UDP-sugar-dependent glycosyltransferase 52 OS=Dictyostelium discoideum GN=ugt52 PE=2 SV=1 | stu-miR171b-3p |
| Algae_064-3_Unigene_BMK.34265 | Probable beta-glucosidase L OS=Aspergillus terreus (strain NIH 2624 / FGSC A1156) GN=bglL PE=3 SV=1 | aca-miR-3p-456915 |
| Algae_064-3_Unigene_BMK.34541 | Ferredoxin OS=Peridinium bipes PE=1 SV=1 | aca-miR-3p-456915 |
| Algae_064-3_Unigene_BMK.3458 | Sperm-associated antigen 1 OS=Mus musculus GN=Spag1 PE=1 SV=1 | osa-miR168a-5p |
| Algae_064-3_Unigene_BMK.34958 | Translation initiation factor IF-2 OS=Rhodococcus opacus (strain B4) GN=infB PE=3 SV=1 | stu-miR171b-3p |
| Algae_064-3_Unigene_BMK.35210 |  | sbi-miR169c |
| Algae_064-3_Unigene_BMK.35341 |  | sbi-miR169c |
| Algae_064-3_Unigene_BMK.3582 | Glycoprotein gp2 OS=Equine herpesvirus 1 (strain V592) GN=71 PE=3 SV=1 | osa-miR168a-5p |
| Algae_064-3_Unigene_BMK.35840 | Cathepsin L1 OS=Canis familiaris GN=CTSL1 PE=2 SV=1 | zma-miR529-5p |
| Algae_064-3_Unigene_BMK.36029 |  | rgl-miR5139_L+3 |
| Algae_064-3_Unigene_BMK.36314 | Serine/arginine repetitive matrix protein 2 OS=Homo sapiens GN=SRRM2 PE=1 SV=2 | osa-miR168a-5p |
| Algae_064-3_Unigene_BMK.36328 | Probable phenylalanine-4-hydroxylase 1 OS=Caenorhabditis elegans GN=pah-1 PE=1 SV=2 | tae-miR159a |
| Algae_064-3_Unigene_BMK.36483 | Uncharacterized abhydrolase domain-containing protein DDB_G0269086 OS=Dictyostelium discoideum GN=DDB_G0269086 PE=1 SV=2 | sbi-miR169c |
| Algae_064-3_Unigene_BMK.36527 | Polycystic kidney disease 2-like 1 protein OS=Homo sapiens GN=PKD2L1 PE=1 SV=1 | zma-miR529-5p |
| Algae_064-3_Unigene_BMK.36542 | Translation initiation factor IF-2 OS=Synechococcus sp. (strain ATCC 27264 / PCC 7002 / PR-6) GN=infB PE=3 SV=1 | stu-miR171b-3p |
| Algae_064-3_Unigene_BMK.36579 | Uncharacterized WD repeat-containing protein alr2800 OS=Nostoc sp. (strain PCC 7120 / UTEX 2576) GN=alr2800 PE=4 SV=1 | aca-miR-5p-43924 |
| Algae_064-3_Unigene_BMK.36615 | Cleavage and polyadenylation specificity factor subunit 6 OS=Mus musculus GN=Cpsf6 PE=1 SV=1 | aca-miR-5p-43924 |
| Algae_064-3_Unigene_BMK.3672 | Vesicle-fusing ATPase OS=Arabidopsis thaliana GN=NSF PE=2 SV=2 | rgl-miR5139_L+3 |
| Algae_064-3_Unigene_BMK.36958 |  | bdi-miR7732-3p_L-1_1ss11GC |
| Algae_064-3_Unigene_BMK.37055 | Serine/arginine repetitive matrix protein 1 OS=Homo sapiens GN=SRRM1 PE=1 SV=2 | aca-miR-5p-43924 |
| Algae_064-3_Unigene_BMK.37070 | Basic proline-rich protein OS=Sus scrofa PE=1 SV=2 | aca-miR-3p-456915 |
| Algae_064-3_Unigene_BMK.37241 | Casein kinase II subunit beta OS=Dictyostelium discoideum GN=csnk2b PE=3 SV=1 | sbi-miR169c |
| Algae_064-3_Unigene_BMK.3735 | Formin-like protein 6 OS=Oryza sativa subsp. japonica GN=FH6 PE=2 SV=2 | sbi-miR169c |
| Algae_064-3_Unigene_BMK.38159 | Transcriptional regulator ATRX homolog OS=Caenorhabditis elegans GN=xnp-1 PE=1 SV=1 | zma-miR529-5p |
| Algae_064-3_Unigene_BMK.38172 | Sodium/potassium/calcium exchanger 2 OS=Homo sapiens GN=SLC24A2 PE=2 SV=1 | bdi-miR7732-3p_L-1_1ss11GC |
| Algae_064-3_Unigene_BMK.38230 | Translation initiation factor IF-2 OS=Synechococcus sp. (strain RCC307) GN=infB PE=3 SV=1 | bdi-miR7732-3p_L-1_1ss11GC |
| Algae_064-3_Unigene_BMK.38239 |  | sbi-miR169c |
| Algae_064-3_Unigene_BMK.3839 | Myosin heavy chain, striated muscle OS=Aequipecten irradians PE=1 SV=1 | aca-miR-5p-43924 |
| Algae_064-3_Unigene_BMK.38447 | ABC transporter B family member 12 OS=Arabidopsis thaliana GN=ABCB12 PE=2 SV=2 | sbi-miR169c |
| Algae_064-3_Unigene_BMK.38519 | Serine/arginine repetitive matrix protein 2 OS=Homo sapiens GN=SRRM2 PE=1 SV=2 | aca-miR-5p-43924 |
| Algae_064-3_Unigene_BMK.38679 | Sal-like protein 3 OS=Homo sapiens GN=SALL3 PE=2 SV=2 | aca-miR-5p-43924 |
| Algae_064-3_Unigene_BMK.38713 |  | sbi-miR169c |
| Algae_064-3_Unigene_BMK.38755 | Uncharacterized protein DDB_G0271670 OS=Dictyostelium discoideum GN=DDB_G0271670 PE=4 SV=1 | aca-miR-5p-43924 |
| Algae_064-3_Unigene_BMK.39225 |  | aca-miR-5p-43924 |
| Algae_064-3_Unigene_BMK.39362 | Formin-like protein 5 OS=Oryza sativa subsp. japonica GN=FH5 PE=2 SV=2 | osa-miR168a-5p |
| Algae_064-3_Unigene_BMK.39465 |  | sbi-miR169c |
| Algae_064-3_Unigene_BMK.39492 |  | sbi-miR169c |
| Algae_064-3_Unigene_BMK.39497 |  | tae-miR159a |
| Algae_064-3_Unigene_BMK.3973 |  | sbi-miR169c |
| Algae_064-3_Unigene_BMK.3982 | Mucin-19 OS=Mus musculus GN=Muc19 PE=2 SV=2 | stu-miR171b-3p |
| Algae_064-3_Unigene_BMK.39895 | Histidine-rich glycoprotein OS=Plasmodium lophurae PE=4 SV=1 | aca-miR-5p-43924 |
| Algae_064-3_Unigene_BMK.4013 | DNA polymerase I OS=Escherichia coli (strain K12) GN=polA PE=1 SV=1 | aca-miR-5p-43924 |
| Algae_064-3_Unigene_BMK.40160 | Heparan sulfate glucosamine 3-O-sulfotransferase 3B1 OS=Mus musculus GN=Hs3st3b1 PE=2 SV=1 | sbi-miR169c |
| Algae_064-3_Unigene_BMK.40338 |  | stu-miR171b-3p |
| Algae_064-3_Unigene_BMK.40451 | Translation initiation factor IF-2 OS=Corynebacterium diphtheriae GN=infB PE=3 SV=1 | osa-miR168a-5p |
| Algae_064-3_Unigene_BMK.40488 | Vegetative incompatibility protein HET-E-1 OS=Podospora anserina GN=HET-E1 PE=4 SV=1 | osa-miR168a-5p |
| Algae_064-3_Unigene_BMK.40512 | Serine/arginine repetitive matrix protein 2 OS=Mus musculus GN=Srrm2 PE=1 SV=2 | stu-miR171b-3p |
| Algae_064-3_Unigene_BMK.40518 | Translation initiation factor IF-2 OS=Streptomyces coelicolor GN=infB PE=3 SV=1 | bdi-miR7732-3p_L-1_1ss11GC |
| Algae_064-3_Unigene_BMK.40781 | UPF0497 membrane protein Os02g0134500 OS=Oryza sativa subsp. japonica GN=Os02g0134500 PE=2 SV=2 | aca-miR-5p-43924 |
| Algae_064-3_Unigene_BMK.4093 | Spore coat protein SP96 OS=Dictyostelium discoideum GN=cotA PE=4 SV=2 | stu-miR171b-3p |
| Algae_064-3_Unigene_BMK.40984 | Probable ribosome biogenesis protein RLP24 OS=Danio rerio GN=rsl24d1 PE=2 SV=1 | stu-miR171b-3p |
| Algae_064-3_Unigene_BMK.41105 | Kelch-like protein 35 OS=Mus musculus GN=Klhl35 PE=2 SV=1 | osa-miR168a-5p |
| Algae_064-3_Unigene_BMK.41284 | Uncharacterized protein BHLF1 OS=Epstein-Barr virus (strain B95-8) GN=BHLF1 PE=4 SV=1 | tae-miR159a |
| Algae_064-3_Unigene_BMK.41401 |  | zma-miR529-5p |
| Algae_064-3_Unigene_BMK.41418 | Uncharacterized protein DDB_G0284671 OS=Dictyostelium discoideum GN=DDB_G0284671 PE=4 SV=1 | sbi-miR169c |
| Algae_064-3_Unigene_BMK.41723 | Putative protein TPRXL OS=Homo sapiens GN=TPRXL PE=5 SV=2 | aca-miR-5p-43924 |
| Algae_064-3_Unigene_BMK.4181 | Meiosis protein mei2 OS=Schizosaccharomyces pombe GN=mei2 PE=1 SV=1 | zma-miR529-5p |
| Algae_064-3_Unigene_BMK.41817 | Peptide deformylase 1B, chloroplastic OS=Arabidopsis thaliana GN=PDF1B PE=1 SV=2 | aca-miR-5p-43924 |
| Algae_064-3_Unigene_BMK.41820 | Uncharacterized protein LOC284861 OS=Homo sapiens PE=2 SV=1 | aca-miR-5p-43924 |
| Algae_064-3_Unigene_BMK.4185 | Serine/arginine repetitive matrix protein 2 OS=Homo sapiens GN=SRRM2 PE=1 SV=2 | osa-miR2876-3p_R+1 |
| Algae_064-3_Unigene_BMK.42176 |  | zma-miR529-5p |
| Algae_064-3_Unigene_BMK.42195 |  | aca-miR-5p-43924 |
| Algae_064-3_Unigene_BMK.42300 | Dynein heavy chain 10, axonemal OS=Homo sapiens GN=DNAH10 PE=1 SV=4 | aca-miR-3p-456915 |
| Algae_064-3_Unigene_BMK.42322 |  | sbi-miR169c |
| Algae_064-3_Unigene_BMK.4279 | Casein kinase I isoform alpha OS=Ovis aries GN=CSNK1A1 PE=3 SV=1 | stu-miR171b-3p |
| Algae_064-3_Unigene_BMK.42894 | Putative DNA helicase ino80 OS=Lodderomyces elongisporus GN=INO80 PE=3 SV=1 | aca-miR-3p-456915 |
| Algae_064-3_Unigene_BMK.42964 | Translation initiation factor IF-2 OS=Rhodococcus sp. (strain RHA1) GN=infB PE=3 SV=1 | osa-miR168a-5p |
| Algae_064-3_Unigene_BMK.42983 |  | sbi-miR169c |
| Algae_064-3_Unigene_BMK.430 | 30S ribosomal protein S1 OS=Staphylococcus epidermidis (strain ATCC 35984 / RP62A) GN=rpsA PE=3 SV=1 | aca-miR-3p-456915 |
| Algae_064-3_Unigene_BMK.4301 | Aspartate-semialdehyde dehydrogenase OS=Aquifex aeolicus GN=asd PE=3 SV=1 | stu-miR171b-3p |
| Algae_064-3_Unigene_BMK.43050 | DNA-directed RNA polymerases I, II, and III subunit RPABC3 OS=Mus musculus GN=Polr2h PE=2 SV=3 | aca-miR-5p-43924 |
| Algae_064-3_Unigene_BMK.43088 | YLP motif-containing protein 1 OS=Homo sapiens GN=YLPM1 PE=1 SV=3 | rgl-miR5139_L+3 |
| Algae_064-3_Unigene_BMK.432 | Peroxiredoxin-2E-2, chloroplastic OS=Oryza sativa subsp. japonica GN=PRXIIE-2 PE=1 SV=1 | stu-miR171b-3p |
| Algae_064-3_Unigene_BMK.43517 | Formin-like protein 6 OS=Oryza sativa subsp. japonica GN=FH6 PE=2 SV=2 | stu-miR171b-3p |
| Algae_064-3_Unigene_BMK.4371 | Protein aardvark OS=Dictyostelium discoideum GN=aarA PE=2 SV=1 | osa-miR168a-5p |
| Algae_064-3_Unigene_BMK.45087 | H(+)/Cl(-) exchange transporter 7 OS=Homo sapiens GN=CLCN7 PE=1 SV=2 | stu-miR171b-3p |
| Algae_064-3_Unigene_BMK.4533 | Flocculation protein FLO11 OS=Saccharomyces cerevisiae GN=MUC1 PE=1 SV=2 | sbi-miR169c |
| Algae_064-3_Unigene_BMK.45401 |  | osa-miR168a-5p |
| Algae_064-3_Unigene_BMK.45485 | Serine/arginine repetitive matrix protein 2 OS=Homo sapiens GN=SRRM2 PE=1 SV=2 | sbi-miR169c |
| Algae_064-3_Unigene_BMK.45529 | Putative protein TPRXL OS=Homo sapiens GN=TPRXL PE=5 SV=2 | aca-miR-3p-456915 |
| Algae_064-3_Unigene_BMK.45916 | Photosystem I P700 chlorophyll a apoprotein A1 OS=Heterocapsa triquetra GN=psaA PE=3 SV=1 | tae-miR159a |
| Algae_064-3_Unigene_BMK.4600 | Translation initiation factor IF-2 OS=Frankia alni (strain ACN14a) GN=infB PE=3 SV=1 | sbi-miR169c |
| Algae_064-3_Unigene_BMK.46079 | Tetracycline resistance protein, class A OS=Escherichia coli GN=tetA PE=3 SV=2 | zma-miR529-5p |
| Algae_064-3_Unigene_BMK.46088 |  | aca-miR-5p-43924 |
| Algae_064-3_Unigene_BMK.46235 | Vegetative cell wall protein gp1 OS=Chlamydomonas reinhardtii GN=GP1 PE=2 SV=1 | sbi-miR169c |
| Algae_064-3_Unigene_BMK.4674 | AP-1 complex subunit gamma-2 OS=Arabidopsis thaliana GN=At1g60070 PE=2 SV=2 | zma-miR529-5p |
| Algae_064-3_Unigene_BMK.46798 | Uncharacterized abhydrolase domain-containing protein DDB_G0269086 OS=Dictyostelium discoideum GN=DDB_G0269086 PE=1 SV=2 | sbi-miR169c |
| Algae_064-3_Unigene_BMK.47053 | Immediate-early protein OS=Saimiriine herpesvirus 2 (strain 11) GN=73 PE=3 SV=1 | sbi-miR169c |
| Algae_064-3_Unigene_BMK.47144 |  | sbi-miR169c |
| Algae_064-3_Unigene_BMK.47423 |  | bdi-miR7732-3p_L-1_1ss11GC |
| Algae_064-3_Unigene_BMK.4763 | Putative protein TPRXL OS=Homo sapiens GN=TPRXL PE=5 SV=2 | aca-miR-5p-43924 |
| Algae_064-3_Unigene_BMK.4774 | NADPH--cytochrome P450 reductase OS=Musca domestica PE=2 SV=1 | stu-miR171b-3p |
| Algae_064-3_Unigene_BMK.4812 | Loricrin OS=Mus musculus GN=Lor PE=2 SV=2 | osa-miR168a-5p |
| Algae_064-3_Unigene_BMK.48173 | V-type proton ATPase 16 kDa proteolipid subunit OS=Pleurochrysis carterae GN=VAP PE=2 SV=1 | rgl-miR5139_L+3 |
| Algae_064-3_Unigene_BMK.48356 | Glyoxylate reductase OS=Thermofilum pendens (strain Hrk 5) GN=gyaR PE=3 SV=1 | stu-miR171b-3p |
| Algae_064-3_Unigene_BMK.48387 | E3 ubiquitin-protein ligase HERC2 OS=Mus musculus GN=Herc2 PE=1 SV=2 | osa-miR168a-5p |
| Algae_064-3_Unigene_BMK.4869 |  | osa-miR168a-5p |
| Algae_064-3_Unigene_BMK.48913 | Vegetative cell wall protein gp1 OS=Chlamydomonas reinhardtii GN=GP1 PE=2 SV=1 | aca-miR-5p-43924 |
| Algae_064-3_Unigene_BMK.49203 | Pentatricopeptide repeat-containing protein At2g31400, chloroplastic OS=Arabidopsis thaliana GN=At2g31400 PE=2 SV=1 | sbi-miR169c |
| Algae_064-3_Unigene_BMK.49248 | Transposase insH for insertion sequence element IS5-18 OS=Escherichia coli (strain K12) GN=JW5951 PE=3 SV=1 | stu-miR171b-3p |
| Algae_064-3_Unigene_BMK.49372 | Translation initiation factor IF-2 OS=Rhodococcus opacus (strain B4) GN=infB PE=3 SV=1 | bdi-miR7732-3p_L-1_1ss11GC |
| Algae_064-3_Unigene_BMK.49390 | Chromatin modification-related protein eaf-1 OS=Neurospora crassa GN=eaf-1 PE=3 SV=1 | stu-miR171b-3p |
| Algae_064-3_Unigene_BMK.49810 |  | sbi-miR169c |
| Algae_064-3_Unigene_BMK.49986 | Uncharacterized 29.3 kDa protein OS=Orgyia pseudotsugata multicapsid polyhedrosis virus GN=ORF92 PE=4 SV=1 | rgl-miR5139_L+3 |
| Algae_064-3_Unigene_BMK.50042 |  | sbi-miR169c |
| Algae_064-3_Unigene_BMK.5041 | Translation initiation factor IF-2 OS=Nocardia farcinica GN=infB PE=3 SV=2 | tae-miR159a |
| Algae_064-3_Unigene_BMK.50455 | Phosphoenolpyruvate carboxykinase [ATP] OS=Kluyveromyces lactis GN=PCK1 PE=3 SV=2 | zma-miR529-5p |
| Algae_064-3_Unigene_BMK.50509 | Serine/arginine repetitive matrix protein 1 OS=Gallus gallus GN=SRRM1 PE=2 SV=1 | aca-miR-5p-43924 |
| Algae_064-3_Unigene_BMK.50636 | SWI/SNF chromatin-remodeling complex subunit snf22 OS=Schizosaccharomyces pombe GN=snf22 PE=1 SV=2 | osa-miR168a-5p |
| Algae_064-3_Unigene_BMK.5069 | Acetyl-CoA carboxylase 2 OS=Homo sapiens GN=ACACB PE=1 SV=3 | tae-miR159a |
| Algae_064-3_Unigene_BMK.50710 |  | sbi-miR169c |
| Algae_064-3_Unigene_BMK.50952 | Alpha-glucosidase 2 OS=Bacillus thermoamyloliquefaciens PE=3 SV=1 | aca-miR-5p-43924 |
| Algae_064-3_Unigene_BMK.51015 |  | aca-miR-3p-456915 |
| Algae_064-3_Unigene_BMK.51189 | Protein aardvark OS=Dictyostelium discoideum GN=aarA PE=2 SV=1 | stu-miR171b-3p |
| Algae_064-3_Unigene_BMK.5228 | Translation initiation factor IF-2 OS=Frankia sp. (strain CcI3) GN=infB PE=3 SV=1 | rgl-miR5139_L+3 |
| Algae_064-3_Unigene_BMK.52576 |  | aca-miR-5p-43924 |
| Algae_064-3_Unigene_BMK.52591 | Tellurium resistance protein TerZ OS=Serratia marcescens GN=terZ PE=3 SV=1 | osa-miR168a-5p |
| Algae_064-3_Unigene_BMK.52628 |  | sbi-miR169c |
| Algae_064-3_Unigene_BMK.52899 | Translation initiation factor IF-2 OS=Rhodococcus opacus (strain B4) GN=infB PE=3 SV=1 | aca-miR-3p-456915 |
| Algae_064-3_Unigene_BMK.53119 |  | zma-miR529-5p |
| Algae_064-3_Unigene_BMK.53602 | Glycoprotein gp2 OS=Equine herpesvirus 1 (strain V592) GN=71 PE=3 SV=1 | aca-miR-3p-456915 |
| Algae_064-3_Unigene_BMK.53714 | Pentatricopeptide repeat-containing protein At2g31400, chloroplastic OS=Arabidopsis thaliana GN=At2g31400 PE=2 SV=1 | stu-miR171b-3p |
| Algae_064-3_Unigene_BMK.5382 | Uncharacterized protein C10orf95 OS=Homo sapiens GN=C10orf95 PE=2 SV=1 | sbi-miR169c |
| Algae_064-3_Unigene_BMK.5407 | Potassium voltage-gated channel subfamily H member 5 OS=Mus musculus GN=Kcnh5 PE=2 SV=2 | aca-miR-3p-456915 |
| Algae_064-3_Unigene_BMK.54347 |  | aca-miR-3p-456915 |
| Algae_064-3_Unigene_BMK.54645 | Translation initiation factor IF-2 OS=Nocardia farcinica GN=infB PE=3 SV=2 | aca-miR-3p-456915 |
| Algae_064-3_Unigene_BMK.54690 | N-alpha-acetyltransferase 38, NatC auxiliary subunit OS=Pongo abelii GN=NAA38 PE=3 SV=3 | rgl-miR5139_L+3 |
| Algae_064-3_Unigene_BMK.55189 | NADH-cytochrome b5 reductase 1 OS=Bos taurus GN=CYB5R1 PE=2 SV=1 | aca-miR-3p-456915 |
| Algae_064-3_Unigene_BMK.55288 |  | bdi-miR7732-3p_L-1_1ss11GC |
| Algae_064-3_Unigene_BMK.55316 |  | osa-miR168a-5p |
| Algae_064-3_Unigene_BMK.55405 | Lysine histidine transporter-like 3 OS=Arabidopsis thaliana GN=At1g61270 PE=3 SV=2 | aca-miR-3p-456915 |
| Algae_064-3_Unigene_BMK.55460 | Translation initiation factor IF-2 OS=Frankia sp. (strain EAN1pec) GN=infB PE=3 SV=1 | sbi-miR169c |
| Algae_064-3_Unigene_BMK.55703 | Helicase SRCAP OS=Homo sapiens GN=SRCAP PE=1 SV=3 | sbi-miR169c |
| Algae_064-3_Unigene_BMK.55984 |  | stu-miR171b-3p |
| Algae_064-3_Unigene_BMK.5667 | Translation initiation factor IF-2 OS=Kineococcus radiotolerans (strain ATCC BAA-149 / DSM 14245 / SRS30216) GN=infB PE=3 SV=1 | sbi-miR169c |
| Algae_064-3_Unigene_BMK.56889 | Mucin-5B OS=Homo sapiens GN=MUC5B PE=1 SV=3 | zma-miR529-5p |
| Algae_064-3_Unigene_BMK.56965 | Keratin-associated protein 5-5 OS=Mus musculus GN=Krtap5-5 PE=2 SV=1 | aca-miR-5p-43924 |
| Algae_064-3_Unigene_BMK.56972 | Protein arginine N-methyltransferase 7 OS=Drosophila persimilis GN=Art7 PE=3 SV=1 | zma-miR529-5p |
| Algae_064-3_Unigene_BMK.57064 | Putative uncharacterized protein ENSP00000383309 OS=Homo sapiens PE=5 SV=3 | rgl-miR5139_L+3 |
| Algae_064-3_Unigene_BMK.57330 | Uncharacterized protein BHLF1 OS=Epstein-Barr virus (strain B95-8) GN=BHLF1 PE=4 SV=1 | aca-miR-3p-456915 |
| Algae_064-3_Unigene_BMK.57341 | Calcium-dependent protein kinase 2 OS=Plasmodium falciparum (isolate K1 / Thailand) GN=CPK2 PE=1 SV=3 | sbi-miR169c |
| Algae_064-3_Unigene_BMK.5747 | Serine/arginine repetitive matrix protein 1 OS=Gallus gallus GN=SRRM1 PE=2 SV=1 | bdi-miR7732-3p_L-1_1ss11GC |
| Algae_064-3_Unigene_BMK.5758 | Circumsporozoite protein OS=Plasmodium cynomolgi (strain Mulligan/NIH) PE=3 SV=3 | bdi-miR7732-3p_L-1_1ss11GC |
| Algae_064-3_Unigene_BMK.57715 | Cell wall protein AWA1 OS=Saccharomyces cerevisiae GN=AWA1 PE=1 SV=1 | sbi-miR169c |
| Algae_064-3_Unigene_BMK.5773 | DNA-directed RNA polymerase II subunit rpb3 OS=Dictyostelium discoideum GN=polr2c PE=3 SV=1 | rgl-miR5139_L+3 |
| Algae_064-3_Unigene_BMK.57813 | ATP-dependent RNA helicase DDX42 OS=Xenopus laevis GN=ddx42 PE=2 SV=1 | rgl-miR5139_L+3 |
| Algae_064-3_Unigene_BMK.57922 | Serine/arginine repetitive matrix protein 1 OS=Gallus gallus GN=SRRM1 PE=2 SV=1 | aca-miR-5p-43924 |
| Algae_064-3_Unigene_BMK.57983 | MAM domain-containing glycosylphosphatidylinositol anchor protein 2 OS=Mus musculus GN=Mdga2 PE=2 SV=1 | osa-miR168a-5p |
| Algae_064-3_Unigene_BMK.58200 | Mucin-5AC (Fragments) OS=Homo sapiens GN=MUC5AC PE=1 SV=3 | aca-miR-3p-456915 |
| Algae_064-3_Unigene_BMK.5836 | ATP-dependent zinc metalloprotease FtsH OS=Guillardia theta GN=ftsH PE=3 SV=1 | sbi-miR169c |
| Algae_064-3_Unigene_BMK.58560 |  | tae-miR159a |
| Algae_064-3_Unigene_BMK.58593 | Vacuolar cation/proton exchanger 2 OS=Oryza sativa subsp. japonica GN=CAX2 PE=2 SV=2 | aca-miR-3p-456915 |
| Algae_064-3_Unigene_BMK.58930 | Potassium voltage-gated channel subfamily F member 1 OS=Homo sapiens GN=KCNF1 PE=1 SV=1 | sbi-miR169c |
| Algae_064-3_Unigene_BMK.58941 | Photosystem I assembly protein ycf4 OS=Synechocystis sp. (strain ATCC 27184 / PCC 6803 / N-1) GN=ycf4 PE=3 SV=1 | zma-miR529-5p |
| Algae_064-3_Unigene_BMK.59171 |  | stu-miR171b-3p |
| Algae_064-3_Unigene_BMK.59382 | Carotenoid 9,10(9',10')-cleavage dioxygenase 1 OS=Pisum sativum GN=CCD1 PE=2 SV=1 | rgl-miR5139_L+3 |
| Algae_064-3_Unigene_BMK.5950 | ATP-dependent RNA helicase eIF4A OS=Ustilago maydis GN=TIF1 PE=3 SV=1 | rgl-miR5139_L+3 |
| Algae_064-3_Unigene_BMK.5977 | Serine/arginine repetitive matrix protein 1 OS=Pongo abelii GN=SRRM1 PE=2 SV=1 | tae-miR159a |
| Algae_064-3_Unigene_BMK.59804 | Periplasmic AppA protein OS=Escherichia coli (strain K12) GN=appA PE=1 SV=2 | bdi-miR7732-3p_L-1_1ss11GC |
| Algae_064-3_Unigene_BMK.5989 |  | aca-miR-3p-456915 |
| Algae_064-3_Unigene_BMK.5996 |  | osa-miR168a-5p |
| Algae_064-3_Unigene_BMK.60168 |  | rgl-miR5139_L+3 |
| Algae_064-3_Unigene_BMK.6018 |  | zma-miR529-5p |
| Algae_064-3_Unigene_BMK.6090 |  | osa-miR168a-5p |
| Algae_064-3_Unigene_BMK.60967 | 3-oxoacyl-[acyl-carrier-protein] synthase 3 OS=Synechococcus sp. (strain WH7803) GN=fabH PE=3 SV=1 | sbi-miR169c |
| Algae_064-3_Unigene_BMK.6108 |  | osa-miR168a-5p |
| Algae_064-3_Unigene_BMK.61135 |  | aca-miR-3p-456915 |
| Algae_064-3_Unigene_BMK.61272 | Putative protein TPRXL OS=Homo sapiens GN=TPRXL PE=5 SV=2 | zma-miR529-5p |
| Algae_064-3_Unigene_BMK.61299 | Homeobox protein cut-like 1 (Fragment) OS=Canis familiaris GN=CUX1 PE=2 SV=1 | aca-miR-5p-43924 |
| Algae_064-3_Unigene_BMK.6132 | Uncharacterized protein LOC284861 OS=Homo sapiens PE=2 SV=1 | sbi-miR169c |
| Algae_064-3_Unigene_BMK.61363 | Dynein heavy chain, cytoplasmic OS=Caenorhabditis elegans GN=dhc-1 PE=2 SV=1 | aca-miR-5p-43924 |
| Algae_064-3_Unigene_BMK.6148 | Putative protein TPRXL OS=Homo sapiens GN=TPRXL PE=5 SV=2 | osa-miR168a-5p |
| Algae_064-3_Unigene_BMK.6149 | Uncharacterized abhydrolase domain-containing protein DDB_G0269086 OS=Dictyostelium discoideum GN=DDB_G0269086 PE=1 SV=2 | sbi-miR169c |
| Algae_064-3_Unigene_BMK.61513 | Translation initiation factor IF-2 OS=Synechococcus sp. (strain RCC307) GN=infB PE=3 SV=1 | stu-miR171b-3p |
| Algae_064-3_Unigene_BMK.61683 | 2-amino-3-carboxymuconate-6-semialdehyde decarboxylase OS=Homo sapiens GN=ACMSD PE=1 SV=1 | aca-miR-5p-43924 |
| Algae_064-3_Unigene_BMK.6169 | Translation initiation factor IF-2 OS=Synechococcus sp. (strain WH7803) GN=infB PE=3 SV=1 | aca-miR-5p-43924 |
| Algae_064-3_Unigene_BMK.61786 |  | bdi-miR7732-3p_L-1_1ss11GC |
| Algae_064-3_Unigene_BMK.62217 | Calcium-dependent protein kinase 2 OS=Plasmodium falciparum (isolate K1 / Thailand) GN=CPK2 PE=1 SV=3 | sbi-miR169c |
| Algae_064-3_Unigene_BMK.62271 | Putative protein TPRXL OS=Homo sapiens GN=TPRXL PE=5 SV=2 | osa-miR168a-5p |
| Algae_064-3_Unigene_BMK.62371 | Atherin OS=Oryctolagus cuniculus GN=SAMD1 PE=2 SV=1 | osa-miR168a-5p |
| Algae_064-3_Unigene_BMK.62689 | Iron-sulfur cluster co-chaperone protein HscB, mitochondrial OS=Mus musculus GN=Hscb PE=2 SV=2 | sbi-miR169c |
| Algae_064-3_Unigene_BMK.62998 | Probable DNA replication complex GINS protein PSF1 OS=Dictyostelium discoideum GN=gins1 PE=3 SV=1 | sbi-miR169c |
| Algae_064-3_Unigene_BMK.63039 | Mucin-5AC (Fragments) OS=Homo sapiens GN=MUC5AC PE=1 SV=3 | sbi-miR169c |
| Algae_064-3_Unigene_BMK.6339 |  | aca-miR-5p-43924 |
| Algae_064-3_Unigene_BMK.63467 | Serine/arginine repetitive matrix protein 1 OS=Homo sapiens GN=SRRM1 PE=1 SV=2 | stu-miR171b-3p |
| Algae_064-3_Unigene_BMK.63630 | Calpain-15 OS=Mus musculus GN=Solh PE=1 SV=1 | zma-miR529-5p |
| Algae_064-3_Unigene_BMK.63900 | Serine/arginine repetitive matrix protein 2 OS=Homo sapiens GN=SRRM2 PE=1 SV=2 | tae-miR159a |
| Algae_064-3_Unigene_BMK.6395 | Putative monooxygenase Rv1533 OS=Mycobacterium tuberculosis GN=Rv1533 PE=3 SV=1 | aca-miR-3p-456915 |
| Algae_064-3_Unigene_BMK.64033 | 46 kDa FK506-binding nuclear protein OS=Spodoptera frugiperda GN=FKBP46 PE=2 SV=1 | zma-miR529-5p |
| Algae_064-3_Unigene_BMK.6415 | Salivary glue protein Sgs-3 OS=Drosophila erecta GN=Sgs3 PE=2 SV=3 | aca-miR-5p-43924 |
| Algae_064-3_Unigene_BMK.6435 | Uncharacterized protein U88 OS=Human herpesvirus 6A (strain Uganda-1102) GN=U88 PE=4 SV=1 | zma-miR529-5p |
| Algae_064-3_Unigene_BMK.64526 | Pseudouridylate synthase 7 homolog-like protein OS=Danio rerio GN=pus7l PE=2 SV=1 | aca-miR-5p-43924 |
| Algae_064-3_Unigene_BMK.6457 | Putative permease MJ0326 OS=Methanocaldococcus jannaschii GN=MJ0326 PE=3 SV=1 | tae-miR159a |
| Algae_064-3_Unigene_BMK.6458 | Mucin-5B OS=Homo sapiens GN=MUC5B PE=1 SV=3 | aca-miR-3p-456915 |
| Algae_064-3_Unigene_BMK.64700 | Mucin-19 OS=Mus musculus GN=Muc19 PE=2 SV=2 | aca-miR-5p-43924 |
| Algae_064-3_Unigene_BMK.64851 | Sodium channel protein type 4 subunit alpha B OS=Tetraodon nigroviridis GN=scn4ab PE=3 SV=1 | sbi-miR169c |
| Algae_064-3_Unigene_BMK.64873 | Translation initiation factor IF-2 OS=Frankia sp. (strain EAN1pec) GN=infB PE=3 SV=1 | zma-miR529-5p |
| Algae_064-3_Unigene_BMK.65037 | Zinc finger protein 828 OS=Homo sapiens GN=ZNF828 PE=1 SV=2 | stu-miR171b-3p |
| Algae_064-3_Unigene_BMK.65241 |  | sbi-miR169c |
| Algae_064-3_Unigene_BMK.65372 | Serine/arginine repetitive matrix protein 1 OS=Pongo abelii GN=SRRM1 PE=2 SV=1 | aca-miR-5p-43924 |
| Algae_064-3_Unigene_BMK.6557 | Translation initiation factor IF-2 OS=Anaeromyxobacter dehalogenans (strain 2CP-C) GN=infB PE=3 SV=1 | tae-miR159a |
| Algae_064-3_Unigene_BMK.6558 | Ribose-phosphate pyrophosphokinase OS=Marinomonas sp. (strain MWYL1) GN=prs PE=3 SV=1 | osa-miR168a-5p |
| Algae_064-3_Unigene_BMK.65937 | Probable L-ascorbate peroxidase 3 OS=Oryza sativa subsp. japonica GN=APX3 PE=2 SV=1 | aca-miR-5p-43924 |
| Algae_064-3_Unigene_BMK.6600 | Putative protein TPRXL OS=Homo sapiens GN=TPRXL PE=5 SV=2 | rgl-miR5139_L+3 |
| Algae_064-3_Unigene_BMK.6643 | Ice-structuring glycoprotein (Fragment) OS=Notothenia coriiceps neglecta GN=afgp8 PE=1 SV=2 | sbi-miR169c |
| Algae_064-3_Unigene_BMK.66521 | 3',5'-cyclic-nucleotide phosphodiesterase regA OS=Dictyostelium discoideum GN=regA PE=1 SV=1 | sbi-miR169c |
| Algae_064-3_Unigene_BMK.66524 |  | sbi-miR169c |
| Algae_064-3_Unigene_BMK.67319 | Glyoxal reductase OS=Bacillus subtilis GN=yvgN PE=1 SV=1 | aca-miR-5p-43924 |
| Algae_064-3_Unigene_BMK.6762 | Probable serine/threonine-protein kinase fhkC OS=Dictyostelium discoideum GN=fhkC PE=3 SV=2 | bdi-miR7732-3p_L-1_1ss11GC |
| Algae_064-3_Unigene_BMK.67702 | Ribosomal RNA small subunit methyltransferase NEP1 OS=Drosophila melanogaster GN=CG3527 PE=3 SV=2 | osa-miR2876-3p_R+1 |
| Algae_064-3_Unigene_BMK.6788 |  | sbi-miR169c |
| Algae_064-3_Unigene_BMK.67910 |  | aca-miR-3p-456915 |
| Algae_064-3_Unigene_BMK.6792 | DEAD-box ATP-dependent RNA helicase 42 OS=Oryza sativa subsp. japonica GN=Os08g0159900 PE=2 SV=1 | aca-miR-3p-456915 |
| Algae_064-3_Unigene_BMK.67929 |  | bdi-miR7732-3p_L-1_1ss11GC |
| Algae_064-3_Unigene_BMK.6800 | Putative sodium-coupled neutral amino acid transporter 11 OS=Mus musculus GN=Slc38a11 PE=2 SV=2 | tae-miR159a |
| Algae_064-3_Unigene_BMK.68166 | Proline-rich protein HaeIII subfamily 1 OS=Mus musculus GN=Prh1 PE=2 SV=2 | aca-miR-5p-43924 |
| Algae_064-3_Unigene_BMK.68228 |  | aca-miR-5p-43924 |
| Algae_064-3_Unigene_BMK.6834 |  | osa-miR168a-5p |
| Algae_064-3_Unigene_BMK.6888 | Dynamin-like protein C OS=Dictyostelium discoideum GN=dlpC PE=2 SV=2 | sbi-miR169c |
| Algae_064-3_Unigene_BMK.69054 |  | zma-miR529-5p |
| Algae_064-3_Unigene_BMK.69449 | Heterogeneous nuclear ribonucleoprotein A3 homolog 1 OS=Xenopus laevis PE=2 SV=1 | osa-miR168a-5p |
| Algae_064-3_Unigene_BMK.69561 | Protein kinase kin1 OS=Schizosaccharomyces pombe GN=kin1 PE=1 SV=3 | osa-miR168a-5p |
| Algae_064-3_Unigene_BMK.7018 | Probable casein kinase I homolog ECU11_1980 OS=Encephalitozoon cuniculi GN=ECU11_1980 PE=3 SV=1 | sbi-miR169c |
| Algae_064-3_Unigene_BMK.70194 | Uncharacterized protein BHLF1 OS=Epstein-Barr virus (strain B95-8) GN=BHLF1 PE=4 SV=1 | stu-miR171b-3p |
| Algae_064-3_Unigene_BMK.70212 | Translation initiation factor IF-2 OS=Frankia alni (strain ACN14a) GN=infB PE=3 SV=1 | zma-miR529-5p |
| Algae_064-3_Unigene_BMK.7032 | Probable alpha-ketoglutarate-dependent dioxygenase ABH6 OS=Mus musculus GN=Alkbh6 PE=2 SV=2 | zma-miR529-5p |
| Algae_064-3_Unigene_BMK.70358 | Basic proline-rich protein OS=Sus scrofa PE=1 SV=2 | aca-miR-5p-43924 |
| Algae_064-3_Unigene_BMK.70367 | Eukaryotic initiation factor iso-4F subunit p82-34 OS=Triticum aestivum PE=1 SV=2 | rgl-miR5139_L+3 |
| Algae_064-3_Unigene_BMK.7042 | Protein dodo OS=Drosophila melanogaster GN=dod PE=1 SV=3 | aca-miR-5p-43924 |
| Algae_064-3_Unigene_BMK.70623 | Uncharacterized serine/threonine-rich protein PB15E9.01c OS=Schizosaccharomyces pombe GN=SPAPB15E9.01c PE=2 SV=2 | sbi-miR169c |
| Algae_064-3_Unigene_BMK.71529 | Translation initiation factor IF-2 OS=Azorhizobium caulinodans (strain ATCC 43989 / DSM 5975 / ORS 571) GN=infB PE=3 SV=1 | aca-miR-3p-456915 |
| Algae_064-3_Unigene_BMK.71855 | Serine/arginine repetitive matrix protein 2 OS=Mus musculus GN=Srrm2 PE=1 SV=2 | aca-miR-5p-43924 |
| Algae_064-3_Unigene_BMK.72067 | Argininosuccinate lyase 2 OS=Rhizobium loti GN=argH2 PE=3 SV=1 | osa-miR168a-5p |
| Algae_064-3_Unigene_BMK.7255 | Serine/threonine-protein kinase Nek1 OS=Homo sapiens GN=NEK1 PE=1 SV=2 | sbi-miR169c |
| Algae_064-3_Unigene_BMK.73008 |  | stu-miR171b-3p |
| Algae_064-3_Unigene_BMK.7305 |  | bdi-miR7732-3p_L-1_1ss11GC |
| Algae_064-3_Unigene_BMK.73268 |  | sbi-miR169c |
| Algae_064-3_Unigene_BMK.73329 | Uridine phosphorylase 1 OS=Homo sapiens GN=UPP1 PE=1 SV=1 | osa-miR168a-5p |
| Algae_064-3_Unigene_BMK.73447 | Uncharacterized 35.5 kDa protein in transposon Tn4556 OS=Streptomyces fradiae PE=4 SV=1 | aca-miR-5p-43924 |
| Algae_064-3_Unigene_BMK.73764 | Cell wall integrity and stress response component 1 OS=Schizosaccharomyces pombe GN=wsc1 PE=1 SV=3 | osa-miR168a-5p |
| Algae_064-3_Unigene_BMK.7391 | Flavohemoprotein OS=Bacillus subtilis GN=hmp PE=2 SV=1 | aca-miR-3p-456915 |
| Algae_064-3_Unigene_BMK.73965 |  | aca-miR-5p-43924 |
| Algae_064-3_Unigene_BMK.74009 | Calcium-dependent protein kinase 16 OS=Arabidopsis thaliana GN=CPK16 PE=1 SV=1 | aca-miR-5p-43924 |
| Algae_064-3_Unigene_BMK.7465 | Translation initiation factor IF-2 OS=Frankia sp. (strain CcI3) GN=infB PE=3 SV=1 | aca-miR-3p-456915 |
| Algae_064-3_Unigene_BMK.74978 | Translation initiation factor IF-2 OS=Rhodococcus opacus (strain B4) GN=infB PE=3 SV=1 | sbi-miR169c |
| Algae_064-3_Unigene_BMK.75086 | Mucin-19 OS=Mus musculus GN=Muc19 PE=2 SV=2 | sbi-miR169c |
| Algae_064-3_Unigene_BMK.75160 |  | stu-miR171b-3p |
| Algae_064-3_Unigene_BMK.75213 | Serine/threonine-protein phosphatase 6 regulatory ankyrin repeat subunit C OS=Danio rerio GN=ankrd52 PE=2 SV=1 | sbi-miR169c |
| Algae_064-3_Unigene_BMK.7525 |  | sbi-miR169c |
| Algae_064-3_Unigene_BMK.75873 |  | sbi-miR169c |
| Algae_064-3_Unigene_BMK.759 | Mucin-19 OS=Mus musculus GN=Muc19 PE=2 SV=2 | sbi-miR169c |
| Algae_064-3_Unigene_BMK.76023 | Immediate-early protein 2 OS=Human herpesvirus 6A (strain GS) GN=U90/U86 PE=1 SV=1 | sbi-miR169c |
| Algae_064-3_Unigene_BMK.7623 |  | zma-miR529-5p |
| Algae_064-3_Unigene_BMK.7637 | Serine/arginine repetitive matrix protein 1 OS=Homo sapiens GN=SRRM1 PE=1 SV=2 | osa-miR168a-5p |
| Algae_064-3_Unigene_BMK.76619 | 60S ribosomal protein L13a OS=Cyanophora paradoxa PE=2 SV=1 | zma-miR529-5p |
| Algae_064-3_Unigene_BMK.7672 | Translation initiation factor IF-2 OS=Frankia alni (strain ACN14a) GN=infB PE=3 SV=1 | sbi-miR169c |
| Algae_064-3_Unigene_BMK.76782 | Serine/arginine repetitive matrix protein 2 OS=Mus musculus GN=Srrm2 PE=1 SV=2 | zma-miR529-5p |
| Algae_064-3_Unigene_BMK.76799 | Basic juvenile hormone-suppressible protein 2 OS=Trichoplusia ni GN=BJSP-2 PE=1 SV=1 | sbi-miR169c |
| Algae_064-3_Unigene_BMK.77244 | Serine/threonine-protein kinase Nek4 OS=Mus musculus GN=Nek4 PE=2 SV=1 | sbi-miR169c |
| Algae_064-3_Unigene_BMK.77264 | Deneddylase OS=Gallid herpesvirus 2 (strain Chicken/Md5/ATCC VR-987) GN=MDV049 PE=3 SV=1 | aca-miR-5p-43924 |
| Algae_064-3_Unigene_BMK.77266 |  | osa-miR168a-5p |
| Algae_064-3_Unigene_BMK.77327 |  | stu-miR171b-3p |
| Algae_064-3_Unigene_BMK.77368 | Serine/arginine repetitive matrix protein 2 OS=Homo sapiens GN=SRRM2 PE=1 SV=2 | aca-miR-3p-456915 |
| Algae_064-3_Unigene_BMK.7737 | Translation initiation factor IF-2 OS=Frankia sp. (strain EAN1pec) GN=infB PE=3 SV=1 | zma-miR529-5p |
| Algae_064-3_Unigene_BMK.77402 | Spermidine synthase 1 OS=Aquifex aeolicus GN=speE1 PE=3 SV=1 | zma-miR529-5p |
| Algae_064-3_Unigene_BMK.77610 | Putative protein TPRXL OS=Homo sapiens GN=TPRXL PE=5 SV=2 | sbi-miR169c |
| Algae_064-3_Unigene_BMK.7769 | Diacylglycerol O-acyltransferase 2B OS=Umbelopsis ramanniana GN=DGAT2B PE=1 SV=1 | rgl-miR5139_L+3 |
| Algae_064-3_Unigene_BMK.77772 | Basic proline-rich protein OS=Sus scrofa PE=1 SV=2 | aca-miR-3p-456915 |
| Algae_064-3_Unigene_BMK.77842 | Translation initiation factor IF-2 OS=Mycobacterium sp. (strain MCS) GN=infB PE=3 SV=1 | aca-miR-5p-43924 |
| Algae_064-3_Unigene_BMK.78011 |  | zma-miR529-5p |
| Algae_064-3_Unigene_BMK.78186 | Uncharacterized RING finger protein P32A8.03c OS=Schizosaccharomyces pombe GN=SPAP32A8.03c PE=2 SV=1 | osa-miR168a-5p |
| Algae_064-3_Unigene_BMK.7829 | Uncharacterized protein LOC284861 OS=Homo sapiens PE=2 SV=1 | rgl-miR5139_L+3 |
| Algae_064-3_Unigene_BMK.78612 | Formin-like protein 5 OS=Oryza sativa subsp. japonica GN=FH5 PE=2 SV=2 | sbi-miR169c |
| Algae_064-3_Unigene_BMK.78748 |  | zma-miR529-5p |
| Algae_064-3_Unigene_BMK.7885 | Momilactone A synthase OS=Oryza sativa subsp. japonica GN=Os04g0179200 PE=2 SV=1 | stu-miR171b-3p |
| Algae_064-3_Unigene_BMK.78963 |  | zma-miR529-5p |
| Algae_064-3_Unigene_BMK.790 | Protein NLRC3 OS=Homo sapiens GN=NLRC3 PE=2 SV=2 | bdi-miR7732-3p_L-1_1ss11GC |
| Algae_064-3_Unigene_BMK.798 |  | aca-miR-5p-43924 |
| Algae_064-3_Unigene_BMK.7991 | 3-demethylubiquinone-9 3-methyltransferase OS=Methylocella silvestris (strain BL2 / DSM 15510 / NCIMB 13906) GN=ubiG PE=3 SV=1 | stu-miR171b-3p |
| Algae_064-3_Unigene_BMK.7999 |  | bdi-miR7732-3p_L-1_1ss11GC |
| Algae_064-3_Unigene_BMK.8000 | Cell wall integrity and stress response component 1 OS=Schizosaccharomyces pombe GN=wsc1 PE=1 SV=3 | sbi-miR169c |
| Algae_064-3_Unigene_BMK.80008 | Serine/arginine repetitive matrix protein 2 OS=Mus musculus GN=Srrm2 PE=1 SV=2 | rgl-miR5139_L+3 |
| Algae_064-3_Unigene_BMK.80069 | Peptidyl-prolyl cis-trans isomerase B OS=Aspergillus niger GN=cypB PE=3 SV=1 | bdi-miR7732-3p_L-1_1ss11GC |
| Algae_064-3_Unigene_BMK.80206 |  | aca-miR-5p-43924 |
| Algae_064-3_Unigene_BMK.81648 |  | osa-miR168a-5p |
| Algae_064-3_Unigene_BMK.81889 |  | aca-miR-3p-456915 |
| Algae_064-3_Unigene_BMK.81931 | Translation initiation factor IF-2 OS=Rhodococcus opacus (strain B4) GN=infB PE=3 SV=1 | zma-miR529-5p |
| Algae_064-3_Unigene_BMK.82396 | Uncharacterized serine/threonine-rich protein PB15E9.01c OS=Schizosaccharomyces pombe GN=SPAPB15E9.01c PE=2 SV=2 | sbi-miR169c |
| Algae_064-3_Unigene_BMK.82709 |  | sbi-miR169c |
| Algae_064-3_Unigene_BMK.82716 |  | osa-miR2876-3p_R+1 |
| Algae_064-3_Unigene_BMK.83003 | High mobility group protein Z OS=Drosophila melanogaster GN=HmgZ PE=1 SV=1 | aca-miR-5p-43924 |
| Algae_064-3_Unigene_BMK.83133 | Translation initiation factor IF-2 OS=Frankia alni (strain ACN14a) GN=infB PE=3 SV=1 | aca-miR-5p-43924 |
| Algae_064-3_Unigene_BMK.83160 | Cysteine synthase A OS=Escherichia coli (strain K12) GN=cysK PE=1 SV=2 | tae-miR159a |
| Algae_064-3_Unigene_BMK.83495 | Uncharacterized transporter YdhK OS=Escherichia coli (strain K12) GN=ydhK PE=3 SV=1 | stu-miR171b-3p |
| Algae_064-3_Unigene_BMK.8361 | Peroxisomal 2,4-dienoyl-CoA reductase OS=Arabidopsis thaliana GN=At3g12800 PE=2 SV=1 | rgl-miR5139_L+3 |
| Algae_064-3_Unigene_BMK.83623 | Serine/arginine repetitive matrix protein 3 OS=Homo sapiens GN=SRRM3 PE=2 SV=4 | stu-miR171b-3p |
| Algae_064-3_Unigene_BMK.83974 | Flocculation protein FLO11 OS=Saccharomyces cerevisiae GN=MUC1 PE=1 SV=2 | aca-miR-5p-43924 |
| Algae_064-3_Unigene_BMK.84129 | Serine/arginine repetitive matrix protein 2 OS=Homo sapiens GN=SRRM2 PE=1 SV=2 | osa-miR168a-5p |
| Algae_064-3_Unigene_BMK.84521 |  | aca-miR-5p-43924 |
| Algae_064-3_Unigene_BMK.8456 | Putative uncharacterized protein DDB_G0275629 OS=Dictyostelium discoideum GN=DDB_G0275629 PE=4 SV=1 | aca-miR-5p-43924 |
| Algae_064-3_Unigene_BMK.8457 | Putative COBW domain-containing protein 7 OS=Homo sapiens GN=CBWD7 PE=5 SV=3 | tae-miR159a |
| Algae_064-3_Unigene_BMK.84777 | Serine/arginine repetitive matrix protein 2 OS=Homo sapiens GN=SRRM2 PE=1 SV=2 | rgl-miR5139_L+3 |
| Algae_064-3_Unigene_BMK.84998 | Proton-coupled amino acid transporter 3 OS=Homo sapiens GN=SLC36A3 PE=1 SV=2 | sbi-miR169c |
| Algae_064-3_Unigene_BMK.85203 |  | zma-miR529-5p |
| Algae_064-3_Unigene_BMK.8548 | ADP-ribose pyrophosphatase OS=Bacillus subtilis GN=nudF PE=1 SV=1 | sbi-miR169c |
| Algae_064-3_Unigene_BMK.85654 | Mucin-19 OS=Mus musculus GN=Muc19 PE=2 SV=2 | sbi-miR169c |
| Algae_064-3_Unigene_BMK.86307 | Basic proline-rich protein OS=Sus scrofa PE=1 SV=2 | aca-miR-5p-43924 |
| Algae_064-3_Unigene_BMK.86459 |  | osa-miR168a-5p |
| Algae_064-3_Unigene_BMK.8647 | DNA mismatch repair protein Msh6-1 OS=Arabidopsis thaliana GN=MSH6-1 PE=2 SV=2 | aca-miR-5p-43924 |
| Algae_064-3_Unigene_BMK.865 |  | tae-miR159a |
| Algae_064-3_Unigene_BMK.86654 |  | osa-miR168a-5p |
| Algae_064-3_Unigene_BMK.87260 |  | aca-miR-5p-43924 |
| Algae_064-3_Unigene_BMK.87324 |  | stu-miR171b-3p |
| Algae_064-3_Unigene_BMK.87427 | Translation initiation factor IF-2 OS=Kineococcus radiotolerans (strain ATCC BAA-149 / DSM 14245 / SRS30216) GN=infB PE=3 SV=1 | rgl-miR5139_L+3 |
| Algae_064-3_Unigene_BMK.8750 |  | tae-miR159a |
| Algae_064-3_Unigene_BMK.88112 |  | aca-miR-5p-43924 |
| Algae_064-3_Unigene_BMK.883 |  | zma-miR529-5p |
| Algae_064-3_Unigene_BMK.88383 |  | bdi-miR7732-3p_L-1_1ss11GC |
| Algae_064-3_Unigene_BMK.884 |  | osa-miR168a-5p |
| Algae_064-3_Unigene_BMK.8843 |  | stu-miR171b-3p |
| Algae_064-3_Unigene_BMK.88764 | Chaperone protein dnaJ 2 OS=Arabidopsis thaliana GN=ATJ2 PE=1 SV=2 | aca-miR-5p-43924 |
| Algae_064-3_Unigene_BMK.88966 | NudC domain-containing protein 2 OS=Rattus norvegicus GN=Nudcd2 PE=2 SV=1 | stu-miR171b-3p |
| Algae_064-3_Unigene_BMK.89145 | 40S ribosomal protein S19-B OS=Schizosaccharomyces pombe GN=rps19b PE=2 SV=2 | sbi-miR169c |
| Algae_064-3_Unigene_BMK.89170 |  | aca-miR-5p-43924 |
| Algae_064-3_Unigene_BMK.89346 | Transmembrane 9 superfamily member 3 OS=Mus musculus GN=Tm9sf3 PE=1 SV=1 | sbi-miR169c |
| Algae_064-3_Unigene_BMK.89560 |  | rgl-miR5139_L+3 |
| Algae_064-3_Unigene_BMK.89633 | Translation initiation factor IF-2 OS=Frankia sp. (strain CcI3) GN=infB PE=3 SV=1 | sbi-miR169c |
| Algae_064-3_Unigene_BMK.90236 | Basic proline-rich protein OS=Sus scrofa PE=1 SV=2 | osa-miR168a-5p |
| Algae_064-3_Unigene_BMK.90296 |  | sbi-miR169c |
| Algae_064-3_Unigene_BMK.9044 |  | bdi-miR7732-3p_L-1_1ss11GC |
| Algae_064-3_Unigene_BMK.91092 | Serine/arginine repetitive matrix protein 1 OS=Gallus gallus GN=SRRM1 PE=2 SV=1 | sbi-miR169c |
| Algae_064-3_Unigene_BMK.9151 | SNF1-related protein kinase catalytic subunit alpha KIN10 OS=Arabidopsis thaliana GN=KIN10 PE=1 SV=2 | aca-miR-5p-43924 |
| Algae_064-3_Unigene_BMK.91666 | Probable DNA repair protein STH1798 OS=Symbiobacterium thermophilum GN=STH1798 PE=3 SV=1 | aca-miR-5p-43924 |
| Algae_064-3_Unigene_BMK.9173 | Uncharacterized protein LOC284861 OS=Homo sapiens PE=2 SV=1 | bdi-miR7732-3p_L-1_1ss11GC |
| Algae_064-3_Unigene_BMK.91985 | Carnosine synthase 1 OS=Gallus gallus GN=CARNS1 PE=1 SV=1 | bdi-miR7732-3p_L-1_1ss11GC |
| Algae_064-3_Unigene_BMK.9206 | Serine/arginine repetitive matrix protein 1 OS=Mus musculus GN=Srrm1 PE=1 SV=1 | osa-miR168a-5p |
| Algae_064-3_Unigene_BMK.92627 | Bifunctional protein aas OS=Pectobacterium carotovorum subsp. carotovorum (strain PC1) GN=aas PE=3 SV=1 | aca-miR-3p-456915 |
| Algae_064-3_Unigene_BMK.92638 | Flocculation protein FLO11 OS=Saccharomyces cerevisiae GN=MUC1 PE=1 SV=2 | osa-miR2876-3p_R+1 |
| Algae_064-3_Unigene_BMK.92639 | Sucrose transport protein SUC2 OS=Arabidopsis thaliana GN=SUC2 PE=1 SV=2 | sbi-miR169c |
| Algae_064-3_Unigene_BMK.92722 | Sodium/proline symporter OS=Salmonella typhimurium GN=putP PE=3 SV=3 | sbi-miR169c |
| Algae_064-3_Unigene_BMK.92867 | Pepsin A OS=Bos taurus GN=PGA PE=1 SV=2 | osa-miR168a-5p |
| Algae_064-3_Unigene_BMK.92894 | Atherin OS=Oryctolagus cuniculus GN=SAMD1 PE=2 SV=1 | osa-miR168a-5p |
| Algae_064-3_Unigene_BMK.93012 | Probable aminopeptidase NPEPL1 OS=Mus musculus GN=Npepl1 PE=2 SV=1 | sbi-miR169c |
| Algae_064-3_Unigene_BMK.93028 | Translation initiation factor IF-2 OS=Synechococcus sp. (strain RCC307) GN=infB PE=3 SV=1 | tae-miR159a |
| Algae_064-3_Unigene_BMK.9329 | Uncharacterized protein DDB_G0271670 OS=Dictyostelium discoideum GN=DDB_G0271670 PE=4 SV=1 | tae-miR159a |
| Algae_064-3_Unigene_BMK.9378 | KDEL-tailed cysteine endopeptidase CEP1 OS=Arabidopsis thaliana GN=CEP1 PE=2 SV=1 | sbi-miR169c |
| Algae_064-3_Unigene_BMK.94 | Calmodulin OS=Triticum aestivum PE=1 SV=3 | sbi-miR169c |
| Algae_064-3_Unigene_BMK.940 | Aminopeptidase N OS=Escherichia coli (strain K12) GN=pepN PE=1 SV=2 | bdi-miR7732-3p_L-1_1ss11GC |
| Algae_064-3_Unigene_BMK.9442 | Glycoprotein gp2 OS=Equine herpesvirus 1 (strain V592) GN=71 PE=3 SV=1 | rgl-miR5139_L+3 |
| Algae_064-3_Unigene_BMK.9667 | Uncharacterized protein DDB_G0271670 OS=Dictyostelium discoideum GN=DDB_G0271670 PE=4 SV=1 | sbi-miR169c |
| Algae_064-3_Unigene_BMK.9988 | Serine/arginine repetitive matrix protein 2 OS=Mus musculus GN=Srrm2 PE=1 SV=2 | osa-miR168a-5p |
| The analysis results were obtained by TargetScan and miRanda. | |  |
